# High-dimensional mapping of human CEACAM1 expression on immune cells and association with melanoma drug resistance

**DOI:** 10.1038/s43856-024-00525-8

**Published:** 2024-07-02

**Authors:** Yu-Hwa Huang, Charles H. Yoon, Amit Gandhi, Thomas Hanley, Carlos Castrillon, Yasuyuki Kondo, Xi Lin, Walter Kim, Chao Yang, Amine Driouchi, Michael Carroll, Scott D. Gray-Owen, Duane R. Wesemann, Charles G. Drake, Monica M. Bertagnolli, Nicole Beauchemin, Richard S. Blumberg

**Affiliations:** 1 Department of Medicine, Brigham and Women’s Hospital, Harvard Medical School, Boston, MA USA; 2 Department of Surgery, Brigham and Women’s Hospital, Harvard Medical School, Boston, MA USA; 3 Program in Cellular and Molecular Medicine, Children’s Hospital Medical Center, Harvard Medical School, Boston, MA USA; 4 Institute of Biomedical Engineering, University of Toronto, Toronto, ON Canada; 5 Department of Molecular Genetics, University of Toronto, Toronto, ON Canada; 6 Division of Allergy and Immunology, Division of Genetics, Brigham and Women’s Hospital and Ragon Institute of MGH, MIT and Harvard, Boston, MA USA; 7 Herbert Irving Comprehensive Cancer Center, Columbia University School of Medicine, New York, NY USA; 8 Rosalind and Morris Goodman Cancer Institute, McGill University, Montreal, QC Canada; 9 Present Address: Department of Internal Medicine, Graduate School of Medicine, Kobe University, Kobe, Japan; 10 Present Address: Janssen R&D, Springhouse, PA USA; 11 Present Address: National Institutes of Health, Bethesda, MD USA

**Keywords:** Immunology, Tumour immunology

## Abstract

**Background:**

Human carcinoembryonic antigen cell adhesion molecule 1 (CEACAM1) is an inhibitory cell surface protein that functions through homophilic and heterophilic ligand binding. Its expression on immune cells in human tumors is poorly understood.

**Methods:**

An antibody that distinguishes human CEACAM1 from other highly related CEACAM family members was labeled with ^159^Tb and inserted into a panel of antibodies that included specificity for programmed cell death protein 1 (PD1) and PD-L1, which are targets of immunotherapy, to gain a data-driven immune cell atlas using cytometry by time-of-flight (CyTOF). A detailed inventory of CEACAM1, PD1, and PD-L1 expression on immune cells in metastatic lesions to lymph node or soft tissues and peripheral blood samples from patients with treatment-naive and -resistant melanoma as well as peripheral blood samples from healthy controls was performed.

**Results:**

CEACAM1 is absent or at low levels on healthy circulating immune cells but is increased on immune cells in peripheral blood and tumors of melanoma patients. The majority of circulating PD1-positive NK cells, innate T cells, B cells, monocytic cells, dendritic cells, and CD4^+^ T cells in the peripheral circulation of treatment-resistant disease co-express CEACAM1 and are demonstrable as discrete populations. CEACAM1 is present on distinct types of cells that are unique to the tumor microenvironment and exhibit expression levels that are highest in treatment resistance; this includes tumor-infiltrating CD8^+^ T cells.

**Conclusions:**

To the best of our knowledge, this work represents the first comprehensive atlas of CEACAM1 expression on immune cells in a human tumor and reveals an important correlation with treatment-resistant disease. These studies suggest that agents targeting CEACAM1 may represent appropriate partners for PD1-related pathway therapies.

## Plain language summary

Some proteins, such as programmed cell death protein 1 (PD1), can stop the immune system from attacking cancer cells, allowing cancers to grow. Therapies targeting these proteins can be highly effective, but tumors can become resistant. It is important to identify factors involved in this resistance to develop improved cancer therapies. Human carcinoembryonic antigen cell adhesion molecule 1 (CEACAM1) is a protein that inhibits an immune response and its levels have been associated with poor patient outcomes. We applied a method that allows for the detection of proteins on a single cell to uncover CEACAM1 patterns in melanoma. We found that increased CEACAM1 expression levels on multiple different immune cell types was associated with tumors that were resistant to therapy. These findings may help us to understand the role of CEACAM1 in cancer and to develop better cancer therapies.

## Abstract (web summary)

Huang, Yoon et al. provide a comprehensive single cell protein atlas of CEACAM1 expression on immune cells in human melanoma samples. Their analysis identifies an association between increased CEACAM1 expression and treatment-resistant disease.

## Introduction

Immune checkpoint blockade (ICB) directed at programmed death ligand 1 (PD1) and its ligand (PD-L1) represent major advances in tumor therapy^1–3^. Nonetheless, a significant proportion of patients harbor tumors that are resistant to these therapies prompting interest in understanding responsible factors and identifying potential new targets for therapy^4^. In addition to PD-L1 and PD-L2^5^, it has recently been recognized that human carcinoembryonic antigen cell adhesion molecule 1 (CEACAM1) may bind to PD1^6^, in addition to its role as a homophilic ligand and receptor for a variety of microbes^7^ and T cell immunoglobulin and mucin domain-containing protein 3 (TIM-3) to mediate inhibitory function^8–12^. Human CEACAM1 is a heavily glycosylated transmembrane protein with 9 potential cell surface isoforms generated by alternate splicing that all contain a membrane-distal ligand-binding immunoglobulin-variable (IgV)-like, N-domain which is linked to a long (L) cytoplasmic tail with two immunoreceptor, tyrosine-based inhibitory motifs (ITIM) or a short (S) cytoplasmic tail that lacks ITIMs^7,13^. The L:S ratio of CEACAM1 potentially determines inhibition associated with CEACAM1 expression^13^. However, very few phenotypic details exist concerning CEACAM1 expression on tumor-associated immune cells despite the recognition that its display in many tumors including melanoma is associated with a poor prognosis^14–16^. A challenge in analyzing these relationships is the high degree of homology between CEACAM1 and other human CEACAM family members such as CEACAM3, CEACAM5, CEACAM6, and CEACAM8 in their IgV-like, membrane-distal N-domain^7,17^ that are also expressed on immune and non-immune cells in the tumor microenvironment (TME)^13,16^. Specifically, the N-domain of CEACAM1 that serves as the primary location of ligand binding exhibits up to 90% similarity to these other CEACAM family members. In this report, we used a CEACAM1-specific antibody together with mass cytometry to provide global insights into the potential cellular basis for CEACAM1’s immune role in melanoma, its association with the state of treatment, and its expression relative to PD1 and PD-L1. Moreover, we sought to uncover CEACAM1 expression patterns that were specific for the TME and/or peripheral blood which could identify potential biomarkers of disease state and outcome. Our studies show that CEACAM1 expression on distinct subsets of B cells, monocytic cells, dendritic cells, and T cells in the TME is associated with treatment-resistant disease.

## Methods

### Patients and clinical samples

All tissue samples were obtained from surgical specimens with informed consent and approval from the Institutional Review Board (IRB) of the Brigham and Women’s Hospital and the Dana-Farber Cancer Institute under protocol #17-000. The surgeon (C.H.Y.) identified operative patients who had generated excess fresh tissue sample during curative or palliative metastasectomies. The surgeon allocated tissue samples at the end of the operation from regions most likely to harbor viable tumor without interfering with diagnosis or clinical staging. The samples were placed in serum-free DMEM media with penicillin/streptomycin antibiotic and transported directly to the laboratory for processing. Per IRB protocol, the tissue samples were de-identified. The patient samples are summarized in Supplementary Table 1 and consisted of treatment-naive and -resistant melanoma patients and in many cases included their paired-peripheral blood samples from which peripheral blood mononuclear cells (PBMC) were obtained by standard methods. These were analyzed by two different antibody panels (Supplementary Table 2) using mass cytometry as indicated. The reagents used are described in Supplementary Table 3. These panels included one that assessed immune cell phenotypes and another the function of T cells. Healthy PBMC were purified from buffy coats obtained from the blood bank from anonymous donors under a discarded specimen protocol approved by the Institutional Review Board of the BWH. The phenotypic panel assessment included healthy donor PBMC (*n* = 5), treatment-naive PBMC (*n* = 7), treatment-resistant PBMC (*n* = 3), treatment-naive tumors (*n* = 9), and treatment-resistant tumors (*n* = 10). For the functional assessment of T cells, the samples included treatment-naive tumors (*n* = 9) and treatment-resistant tumors (*n* = 5) as summarized in Supplementary Table 1.

### Flow cytometry

The 26H7 monoclonal antibody is a mouse IgG1 anti-human CEACAM1-specific antibody previously described. T84.1 (a kind gift of Dr. John E. Shively, City of Hope, Duarte, CA), Col-1 (ThermoFisher Scientific), and 9A6 (ThermoFisher Scientific) antibodies were used as controls. These antibodies or their isotype controls were used to stain Hela cells transfected with vector control (neo), human (h) CEACAM1, hCEACAM3, hCEACAM5, hCEACAM6, and hCEACAM8 (Supplementary Table 3). 1 × 10^6^ Hela transfectant cell lines were incubated with 0.5 μg/ml of each antibody indicated above for 30 min in room temperature followed by washes with FACS buffer and further incubated with secondary FITC conjugated mouse IgG1 or IgG2a antibodies for another 30 min at room temperature. Cells were washed and acquired by a Beckman Coulter cytometer. Data were analyzed by FlowJo and normalized by the emission in FITC fluorescence intensity of CEACAM1, CEACAM3, CEACAM6 or CEACAM8 and calculated, presented as a relative ratio.

### Mutagenesis of human CEACAM1

Point mutations were introduced by PCR-based mutagenesis, using the QuikChange II Site-Directed Mutagenesis Kit (Agilent Technologies). Previously described vectors containing the human CEACAM1-3L variant^8^ in the pDisplay vector (Invitrogen) were used as the template for all mutations. PCR reactions for single amino acid mutations were run for 16 cycles of 30 s at 95 °C and 1 min at 55 °C, followed by 6 min at 68 °C. The resulting mutant plasmids were verified by Sanger DNA sequencing. The CEACAM1 amino acid residues were numbered according to National Center for Biotechnology Information database. The primers used are described as follows. Y34A (5′-actttttggctacagctgggccaaaggggaaagagtggat-3′); Y34A_antisense (5′-atccactctttcccctttggcccagctgtagccaaaaagt-3′); Q44L (5′-gagtggatggcaaccgtctaattgtaggatatgcaata-3′); Q44L_antisense (5′-tattgcatatcctacaattagacggttgccatccactc-3′); Q89A (5′-tgacacaggattctacaccctagcagtcataaagtcagatcttg-3′); Q89A_antisense (5′-caagatctgactttatgactgctagggtgtagaatcctgtgtca-3′); V39A (5′-gtacaaaggggaaagagcggatggcaaccgtcaaa-3′); V39A_antisense (5′-tttgacggttgccatccgctctttcccctttgtac-3′); G41A (5′-aggggaaagagtggatgccaaccgtcaaattgtag-3′); G41A_antisense (5′-ctacaatttgacggttggcatccactctttcccct-3′); G47A (5′-ggcaaccgtcaaattgtagcatatgcaataggaactcaa-3′); G47A_antisense (5′-ttgagttcctattgcatatgctacaatttgacggttgcc-3′); S93A (5′-tacaccctacaagtcataaaggcagatcttgtgaatgaagaag-3′); S93A_antisense (5′-cttcttcattcacaagatctgcctttatgacttgtagggtgta-3′); D94A (5′-ccctacaagtcataaagtcagctcttgtgaatgaagaagcaac-3′); D94A_antisense (5′-gttgcttcttcattcacaagagctgactttatgacttgtaggg-3′); V96A (5′-agtcataaagtcagatcttgcgaatgaagaagcaactggac-3′); V96A_antisense (5′-gtccagttgcttcttcattcgcaagatctgactttatgact-3′).

### Immunoblotting

As previously reported^8^, human embryonic kidney-293T (HEK293T) cells were transfected with the 1200 ng of Flag-tagged human CEACAM1 wild-type or mutant vectors or 1200 ng of vector controls and HEK293T cells transfected for 48 h. Transfected cells were washed once with cold PBS and lysed on ice with 0.5 ml of immunoprecipitation buffer containing 20 mM Tris-HCl, 0.15 M sodium chloride, pH 7.6, with protease inhibitor cocktail tablets (Roche) and 1.0% digitonin (Sigma). After 60 min, the cell lysates were spun at 14,000 r.p.m. for 30 min at 4 °C. The lysate was subsequently washed with immunoprecipitation buffer and re-suspended in 30 μl of Laemmli sample buffer without reducing agents. After boiling for 5 min, the proteins were resolved by SDS–PAGE in regular Tris-glycine buffer on a 4–20% Tris-Glycine Gel (Novex). The proteins were electrically transferred to a PVDF (polyvinylidene difluoride) membrane. After blocking with 5% skim milk in 0.05% PBS-Tween (PBS-T), the membranes were incubated for 12 h at 4 °C with the 26H7 monoclonal antibody (1 μg/ml) or anti-FLAG antibody produced in rabbit (Sigma) at the same concentration. The membranes were further incubated with corresponding mouse IgG1 (26H7) or rabbit (anti-FLAG) secondary antibodies for 1 h at room temperature and visualized by Amersham ECL Western Blotting Detection Reagents (GE Healthcare). Unsaturated films were digitally scanned.

### Protein purification, modification, and surface plasmon resonance binding

Recombinant human (h) CEACAM1 IgV-cys was purified and modified with a C-terminal biotin tag as previously described^8,9^. In order to position hCEACAM1 IgV in a binding receptive orientation, hCEACAM1 IgV-cys-biotin was flowed at monomeric concentrations (50 nM) over a neutravidin coupled CM5 surface to a final bound level of 103 RU as described^8,9^. Anti-hCEACAM1 antibody 26H7 (500 nM) was injected at a flow rate of 30 ml/min in running buffer (10 mM HEPES, 150 mM NaCl, 10 mM CaCl2, pH 7.4) with a 3 s contact time and 300 s dissociation time over the hCEACAM1-containing flow cell and negative control (neutravidin only) flow cell. Determination of 26H7 binding specificity was determined by subtraction of the control binding sensorgram from the experimental flow cell in the Biacore T200 Evaluation Software (GE Healthcare).

### Isolation of peripheral blood mononuclear cells and tumor-dissociated cells

Tumor biopsies were subjected to a commercial mechanical/enzymatic dissociation system (GentleMACS dissociator, Miltenyi Biotec). The enzymatic digest was based upon methodology previously established for the generation of melanoma tumor-infiltrating lymphocytes (TIL)^18^. Briefly, the tumor was cut into small fragments about 2–3 mm in length and put in a C-tube (Miltenyi Biotech) with RPMI 1640 (Lonza, Slough, UK) and solutions 1, 2 and 3 (all from Miltenyi Biotec) according to the manufacturer’s recommendations. The digest mix containing the tumor was then subjected to mechanical disaggregation steps in the GentleMACS dissociator interspersed by two 30-min incubations at 37 °C performed after the first and the second disaggregation steps, respectively. After disaggregation, tumor-associated cells from the enzymatic digest and the GentleMACS dissociation were passed through 100-μm strainers for further analyses. Peripheral blood mononuclear cells (PBMC) were isolated by Ficoll-Hypaque using standard methods.

### Design of two panels that interrogate the complexity of disease-associated immune signatures

An immune-phenotyping Panel consisted of a standard panel from Fluidigm for immune profiling (Maxpar® Direct™ Immune Profiling Assay™) that consisted of 30 cellular parameters (Catalog Number SKU201325) together with ^159^Tb-CEACAM1, ^169^Tm-PD1 (clone number J116, Bio X Cell) and ^175^Lu-PD-L1 (29E.2A3, Bio X Cell) that allowed for an assessment of the expression of these latter three markers on specific immune subsets within PBMC and tumor (Supplementary Table 2). The 26H7, J116, and 29E.2A3 antibodies were conjugated by the Brigham and Women’s Hospital mass cytometry core. Here, an appropriate quantity of carrier-free antibodies was coupled to metal-labeled X8 polymer according to the manufacturer’s instructions (Fluidigm). A second panel consisting of 34 cellular parameters was designed by us to investigate the functional characteristics of immune cell subsets and included T cell exhaustion markers such as TIM-3, LAG3, OX40, ICOS, and 4-1BB as well as intracellular markers that define the functional activities of the T cells such as FOXP3, granzyme-B, IL-5, IL-6, IL-10, and TGF-β in coordination with ^159^Tb-CEACAM1, ^169^Tm-PD1 and ^175^Lu-PD-L1 (Supplementary Table 2).

### Sample preparation procedures for mass cytometry

Following mechanical digestion and disaggregation of tumors into a single-cell suspension and/or paired individual PBMC samples, 5 × 10^5^ freshly isolated cells per sample were transferred to FACS falcon tubes, washed once with CyFACS (metal-free PBS + 4% FCS + 2 mM EDTA) and blocked with 1 μg/ml anti-human CD16/CD32 antibody (BD Biosciences) for 15 min in a final volume of 5 mL CyFACS. To minimize run-to-run variation and facilitate the comparison of cellular profiles from different cell subsets and individuals, a master mix of titrated amounts of metal-labeled antibodies aliquoted and prepared as previously described by others^19^ was added in a 5 mL volume and incubated at 37 °C for 5 min followed by a 45-min incubation at room temperature, with gentle mixing at 15-min intervals. Cells were then washed twice in CyFACS. For intracellular protein detection in the functional cohort study, after an additional wash in CyFACS buffer, cells were incubated in Cytofix (BD Biosciences) for 30 min on ice and subsequently washed in permeabilization buffer (BD Biosciences) prior to intracellular staining with a titrated master mix of metal-labeled antibodies at room temperature for 45 min. The cells were then washed and fixed a second time in 4% paraformaldehyde in PBS at 4 °C containing 62.5 nM iridium nucleic acid intercalator (Fluidigm) for 18–36 h. The cells were then washed once with PBS, once with de-ionized water, and then diluted in de-ionized water containing 10% EQ Calibration Beads (Fluidigm) at 1 million cells per mL before signal acquisition on a CyTOF Helios™ system mass cytometer (Fluidigm) assisted by specialists at the Jimmy Fund Flow Cytometry Core Facility (Dana-Farber Cancer Institute).

### Sample handling for data acquisition

Before data acquisition, the cells were centrifuged, pelleted, and carefully overlaid with a volume of 100 μL nucleic acid Ir-Intercalator (MAXPAR, catalog number 201192B) in 2% PFA/PBS (1:2000) at room temperature for 30 min without disturbing the cell pellets. Subsequently, the cells were washed twice with CyFACS buffer and twice with de-ionized water before a final re-suspension in de-ionized water. Cells were counted and diluted to a concentration of 0.5 × 10^6^ cells/mL. EQ Four Element Calibration Beads (DVS Science, Fluidigm) were added at a 1% concentration prior to acquisition. Cell data were acquired and analyzed using a CyTOF Helios™ system mass cytometer (Fluidigm).

The mass cytometry data were randomized with the Fluidigm acquisition software and normalized with the FlowJo normalizer and deconvolved using the Boolean gating algorithm within FlowJo. The data were exported in a conventional flow cytometry file (.fcs) format. Individual samples were manually gated using Cytobank to exclude normalization beads, cell debris, dead cells, and doublets for the identification of CD45^+^ live cells for further downstream analyses. The identification of cell populations was achieved by “manual gating,” which constituted distinct cell populations on a series of bi-axial plots (dot plots showing the expression of two proteins for all cells) based on prior knowledge and literature validation (Supplementary Table 4).

### Multiplexed single-cell mass cytometry data analysis

For the first phenotype analysis cohort, the mass cytometry files from peripheral blood mononuclear cells (PBMC) from 5 healthy individuals, PBMC from 7 treatment-naive and PBMC from 3 treatment-resistant patients were concatenated into randomly sampled cells using (R script below) for each group. Analogously, samples from the tumors from 9 treatment-naive and 10 treatment-resistant patients were processed similarly. A similar approach was also used for the study patients in the functional panel that consisted of 9 treatment-naive and 5 treatment-resistant tumor samples.

For dimensionality reduction analyses, the viSNE tool was used to apply the Barnes-Hut implementation of the t-Distribution Stochastic Neighbor Embedding (tSNE) algorithm that makes a pairwise comparison of cellular phenotypes to optimally plot similar cells close to each other and reduces multiple parameters into two dimensions (tSNE1 and tSNE2). Each point in the visualized (vi)SNE plot represents a single event (e.g., cell) detected by the mass cytometer and colored according to the cell population identified. The abundance of cellular subsets was shown after the acquisition of equal cell numbers for our global data structure assessment. The observed regional differences in cell densities corresponding to differences in the relative abundance of the major immune cell lineages were highlighted. For the initial global immune architecture analysis, to define the cellular composition, we anchored our data on equal events for each sample among the five concatenated files associated with the different types of study patients and associated samples. Channel selection was consistent throughout the entire data set regardless of the populations. For each immune subset, separate viSNE plots were generated via re-clustering for each immune subset studied. For the internal viSNE parameter settings, we first examined a training set of treatment-naive (*n* = 7) and -resistant (*n* = 13) tumor samples to establish the parameters for the settings. From this analysis, we chose 3000 iterations, perplexity of 30, and theta of 0.5 for cells more than 1 million and 1000 iterations, perplexity of 30, and theta of 0.5 for cells less than 1 million. After the data global structures were visualized using viSNE and their structure dissected into distinct immune populations by re-embedding the manual gates that define them, markers of interest (such as CEACAM1, PD1, and PD-L1) were further exported from the specific immune populations which then were interrogated with appropriate statistical approaches to validate the data variance and significance between groups.

Further, the data were then integrated with high-dimensional analysis algorithms. Algorithms and software kits were applied using Cytobank as a platform to facilitate analysis of CyTOF datasets, including analyses such as FlowSOM, Citrus, and SPADE, all of which were used to identify cell populations by automatically partitioning cells according to the data structure, regardless of prior knowledge, using the embedded algorithms.

For biomarker discovery of which immune population or markers were significantly different between the five groups of this study, we performed analysis to stratify signatures from clustered data features that explain differences between the 5 clinical sample groups by Citrus (cluster identification, characterization, and regression) analysis. The FCS files were normalized, followed by a quality examination and segregation of live cells from each patient of each group. The input was assessed by Significance Analysis of Microarrays (SAM) as a correlative model to identify features that correlate with an endpoint at different false discovery rates. In brief, collected single-cell events were pooled and hierarchically clustered based upon the similarity of expression of subsets of the measured markers/channels and presented as a radial spinning tree. The clusters were then exported, contextualized according to the associated markers and differences between the clinical groups assessed.

To capture the inner structure of the CyTOF data, we used the FlowSOM algorithm to generate a minimum spanning tree (MST) which allowed us to assess the data using pre-selected numbers of metaclusters and clusters. The FlowSOM output as represented by an MST was also contextualized as a hybrid presentation that merged the manual gating and data-driven automatic cellular clustering among the five clinical groups in this study. To investigate the CD4^+^ T cells in individual patients we used the SPADE clustering algorithm to pinpoint the correlations between FoxP3 and CEACAM1 expression.

### R Code for data file processing

devtools::install_github(“r-lib/devtools”)

install.packa: ges(“XML”)

library(“devtools”)

#install.packages(‘rstudioapi’)

#install.packages(“devtools”)

devtools::install_github(“rstudio/rstudioapi”)

#install flowCore

if (!requireNamespace(“BiocManager”, quietly = TRUE))

install.packages(“BiocManager”)

BiocManager::install(“flowCore”)

#install the cytofCore package

#install_github(“nolanlab/cytofCore”)

library(“flowCore”)

library(“cytofCore”)

library(‘rstudioapi’)

directory <- rstudioapi::selectDirectory()

cytofCore::cytofCore.concatenateDirectoryFiles(directory)

### Immunofluorescence confocal microscopy

Fresh tonsil tissues and melanoma patient tissues were embedded in OCT, frozen in dry ice-cold ethanol, and stored at −80 °C. Before cutting, blocks were equilibrated at sectioning temperature (−20 °C). 10–16 μm thick sections were cut on a cryostat and mounted on Super Up-Rite slides (Thermo Scientific). Sections were fixed with 4% PFA for 10 min at RT, rinsed 3× with PBS, and incubated with PBS, 0.2% Triton X-100 for 3–5 min at RT. Slides were washed 3× with PBS, 0.05% Tween-20 and incubated with blocking buffer (PBS, 0.05% Tween-20, 3% BSA, 5% FCS) for 1 h at RT, followed by incubation with primary antibodies specific for human CEACAM1 (26H7 monoclonal antibody, labeled with phycoerythrin using PE Custom Conjugation Kit, BioLegend), Brilliant Violet 421™ anti-human CD19 (clone H1B19, BioLegend) and FITC conjugated, anti-human CD3 (clone BW264/56, Miltenyi Biotec) prepared in blocking buffer and incubated overnight at 4 °C. When 2-step staining was required, slides were washed 3× with PBS, 0.05% Tween-20 before incubation with a secondary mix in blocking buffer for 1 h at RT. At the end of staining sections were washed 3× with PBS, 0.05% Tween-20. Slides were slightly dried and mounted in Fluoro-Gel (Electron Microscopy Sciences) and sealed with coverslips. Imaging was done with a Fluoview FV3000R resonant scanning confocal microscope equipped with 4 laser lines (405, 488, 514, and 633 nm).

### Statistical and reproducibility

A non-parametric test, the Kruskal–Wallis test, was used to compare the specific human immune populations as well as phenotypical assessments among all five independent groups containing healthy donor PBMC (*n* = 5), treatment-naive PBMC (*n* = 7), treatment-resistant PBMC (*n* = 3), treatment-naive tumors (*n* = 9) and treatment-resistant tumors (*n* = 10). All were followed by *post hoc* pairwise Dunn’s multiple comparison test. In the case of CEACAM1 expression on the B cell types and their proportions relative to total CD45^+^ cells, the differences were determined by using the one-sample Wilcoxon signed rank test as the data did not follow a normal distribution. Two-way analysis of variance (ANOVA) was used to analyze differences in CEACAM1 expression on CD4^+^ regulatory T cells among the independent groups. When focusing on the tumor specimens, a two-tailed paired *t*-test was used for parametric data or Wilcoxon matched-paired signed rank test for data points that were not normally distributed (non-parametric data). Differences were defined as statistically significant when adjusted *p* < 0.05. All statistical tests and graphs were performed using GraphPad Prism 9.00 for Mac (GraphPad Software, La Jolla).

### Reporting summary

Further information on research design is available in the Nature Portfolio Reporting Summary linked to this article.

## Results

### Establishment of a mass cytometry pipeline for defining human CEACAM1 expression in the tumor microenvironment

**Fig. 1:**
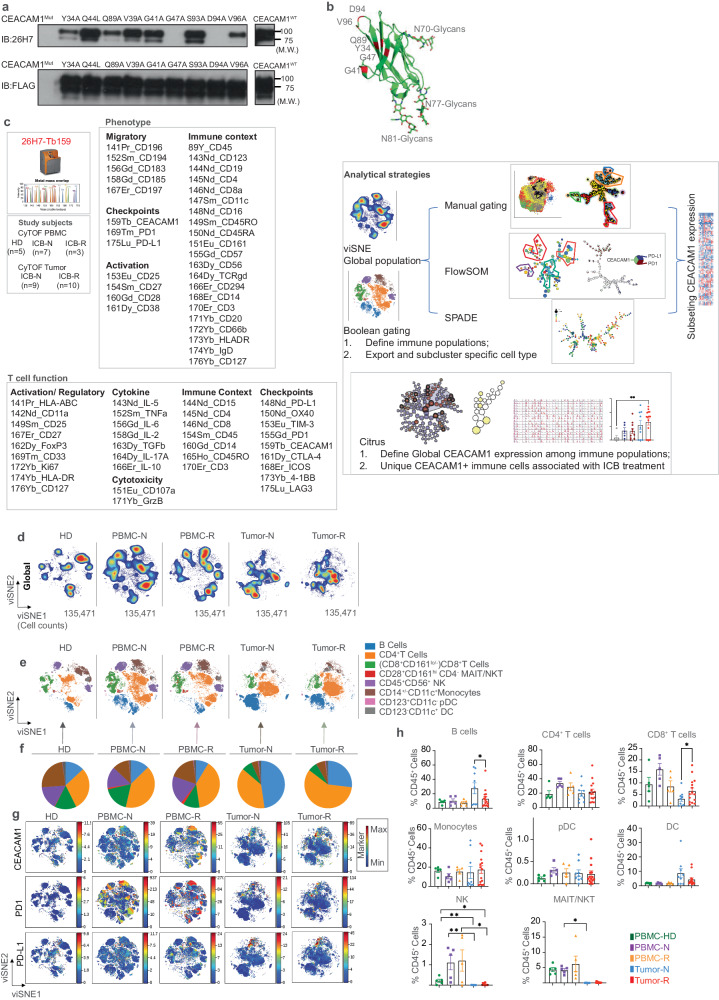
Global characterization of the melanoma samples and their expression of CEACAM1. **a** Immunoblot of human (h)CEACAM1 wild-type (WT) and mutants of amino acids within the IgV-domain face as indicated (Y34A, Q44L, Q89A, V39A, G41A, G47A, S93A, D94A, V96A) expressed as FLAG-tagged proteins in transfected HEK293T cells and immunoblotted (IB) with the 26H7 mAb or a FLAG-tag specific antibody. Molecular weights (M.W.) are indicated on the right of each blot; **b** X-ray crystal structure of human CEACAM1 (PDB 7RPP) with modeled sugar molecules showing CEACAM1 GFCC’ face residues Y34, G41, G47, Q89, D94 and V96 in red which mediate binding to the 26H7 antibody and are located away from the N70, N77 and N81-linked glycans in the ABED face. Glycans are shown by stick representation; **c** Study patients include peripheral blood mononuclear cells (PBMC) from healthy donors (HD, *n* = 5), tumor-associated PBMC from immune checkpoint blockade (ICB) treatment-naive- (N, *n* = 7) and -resistant (R, *n* = 3) and metastatic (to the lymph nodes) tumor nodules from ICB treatment-naive (*n* = 9) and -resistant (*n* = 10) melanoma patients. Thirty-three markers for phenotype assessment and thirty-four markers for functional assessment were included and classified according to their immunological characteristics. Analytic strategies are briefly depicted: To classify CEACAM1 expression in defined immune populations, we first used the global segregation of the immune cells by visualizing and clustering with viSNE (visualized t-distributed stochastic neighbor embedding) and Citrus (cluster identification, characterization, and regression), followed by application of manual (Boolean) gating, FlowSOM (self-organizing map) and/or SPADE (Spanning-tree Progression Analysis of Density-normalized Events) to permit the identification of CEACAM1 within the subsets of the immune cells of the groups. Citrus stratified the disease association of the unique immune subsets that CEACAM1 marks. ICB, immune checkpoint blockade; **d** viSNE visualization of the cellular clustering of peripheral blood mononuclear cells (PBMC) of healthy donors (HD, *n* = 5), treatment-naive (PBMC-N) melanoma patients (*n* = 7) and treatment-resistant (PBMC-R) melanoma patients (*n* = 3) and dissociated tumor cells from treatment-naive (Tumor-N, *n* = 9) and treatment-resistant (Tumor-R, *n* = 10) melanoma patients were stained with an immune-phenotyping panel. All files within each treatment group were concatenated. Single live CD45^+^ cells for each sample and equal numbers of cells were exported (135,471 cells from each sample); **e** Cell types visualized within viSNE space are colored based upon assignments established by manual gating in the types of clinical samples as in (**d**); **f** Pie charts showing the proportions of immune cells identified in (**e**) from an equal quantity of cells in each clinical sample type. Innate T cells including MAIT mucosa-associated invariant T cells, and NKT natural-killer T cells. NK natural-killer cells. DC total dendritic cells. pDC plasmacytoid DC; **g** The location in viSNE space of median CEACAM1 (median value of ^159^Tb) expression levels compared to PD1 (median value of ^169^Tm) and PD-L1 (median value of ^175^Lu) in the global population in the types of clinical samples as in (**d**). The color-coded scale bar for each clinical sample type is shown on the right with minimum (blue) and maximum (red); **h** Distribution as percent (%) of total CD45^+^ cells of each cell type as in (**e**) and in each clinical category as in (**d**). *p < 0.05; **p < 0.01 using a paired *t*-test with two tails, except for NK and MAIT/NKT cells that were evaluated by the Kruskal–Wallis test, followed by Dunn’s multiple comparison test. Error bars on graphs were plotted with the standard error of the mean acquisition.

The 26H7 monoclonal antibody (mAb) is a previously described mouse anti-human CEACAM1 IgV-domain specific mAb^20,21^. Flow cytometry analysis of human CEACAM1, CEACAM3, CEACAM5, CEACAM6, and CEACAM8 transfected Hela cells confirmed 26H7 mAb specificity for human CEACAM1 (Supplementary Fig. 1a–d). Further, immunoblots of human embryonic kidney-293T cells transfected with wild-type or mutated CEACAM1 provided biochemical confirmation for its interaction with the IgV-domain. Mutation of specific amino acid residues within the human CEACAM1 GFCC’ face of this domain diminished binding (Fig. 1a, Supplementary Fig. 1e, f). The GFCC’ face that is involved in homophilic and heterophilic interactions^8^ is devoid of glycosylation predicting that the 26H7 antibody will bind all glycoforms and splice variants of CEACAM1 which all contain the IgV-domain (Fig. 1b)^7^. Indeed, the 26H7 antibody can bind to a bacterially expressed IgV-domain that lacks carbohydrate modifications (Supplementary Fig. 1g)^8,9^. We therefore labeled the 26H7 mAb with ^159^Tb and inserted it into a panel of markers that included PD1 and PD-L1 and can distinguish immune cell subsets^22^ (Supplementary Table 2), using cytometry by time-of-flight (CyTOF) (Fig. 1c). We used these to gain a data-driven, high-dimensional CEACAM1 profile in patients with melanoma relative to healthy donor controls. This immunophenotyping panel was used to characterize the cell types that expressed CEACAM1 on peripheral blood mononuclear cells (PBMC) of 5 healthy donors (HD), PBMC from 7 treatment-naive (PBMC-N), and 3 treatment-resistant (PBMC-R) melanoma patients and cells contained within metastatic lesions to lymph node or soft tissues from 9 treatment-naive (Tumor-N) and 10 treatment-resistant (Tumor-R) samples (Supplementary Table 1). The inclusion of healthy and melanoma-associated PBMC samples that were matched to the tumor samples and stratified upon treatment-naive and -resistant disease status provided an opportunity to define phenotypic traits unique to the TME compared to those present in the circulation.

**Fig. 2:**
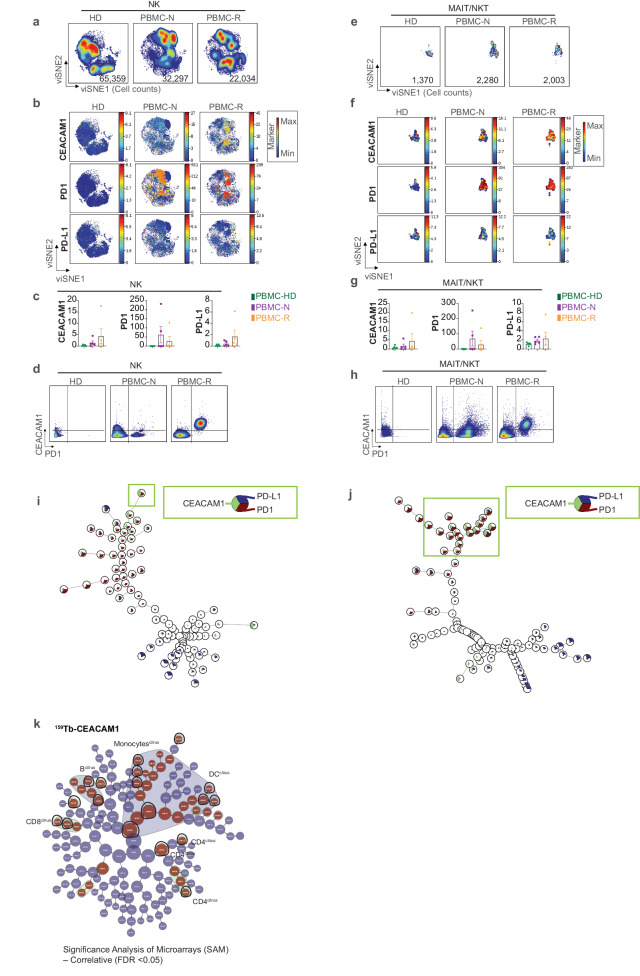
Association of CEACAM1 with disease status. **a** viSNE visualization of proportional numbers of exported natural-killer (NK) cells from healthy donors (HD, *n* = 5), peripheral blood mononuclear cells (PBMC) from treatment-naive (N, *n* = 7) and -resistant (R, *n* = 3) samples. The cell numbers associated with each type of clinical sample are indicated in the bottom of the representative viSNE plot associated with each group; **b** Median expression levels of CEACAM1, PD1, and PD-L1 are visualized on the viSNE maps of NK cells from HD, treatment-naïve and -resistant PBMC samples as in (**a**). The color-coded scale bar for each cell type is shown on the right (blue, minimum; red, maximum); **c** Quantitation of CEACAM1, PD1, and PD-L1 expression levels on NK cells in HD, PBMC-N and PBMC-R samples, error bar on graphs were plotted with standard error of the mean acquisition; **d** Bivariant, dual marker density plots of CEACAM1 and PD1 expression on NK cells in PBMC of HD, PBMC-N and PBMC-R patients; **e** viSNE visualization of proportional numbers of exported mucosal-associated invariant and natural-killer T cells (NKT/MAIT). The cell numbers associated with each type of clinical sample are indicated in the bottom of the representative viSNE plot associated with each group; **f** Median expression levels of CEACAM1, PD1, and PD-L1 is visualized on the viSNE maps of MAIT/NKT cells from HD (*n* = 5), PBMC-N (*n* = 7) and PBMC-R (*n* = 3) samples. The color-coded scale bar for each cell type is shown on the right (blue, minimum; red, maximum); **g** Quantitation of CEACAM1, PD1, and PD-L1 expression levels on MAIT/NKT cells in HD, PBMC-N, and PBMC-R samples as in (**f**), error bar on graphs were plotted with standard error of the mean acquisition; **h** Bivariant, dual marker density plots of CEACAM1 and PD1 expression on MAIT/NKT cells in PBMC of HD, PBMC-N, and PBMC-R samples; **i** Pie-chart depicting CEACAM1, PD1 and PD-L1 expression levels on NK cells in a minimal spanning tree (MST)-associated with FlowSOM analysis of the merged NK cells; green bracket indicating metacluster M5 as in (Supplementary Fig. 2a–c); **j** Pie-chart depicting CEACAM1, PD1, and PD-L1 expression levels on innate T cells in MST; green bracket indicating metacluster M1 as in (Supplementary Fig. 2e–g); **k** Citrus-associated, SAM (Significance analysis of microarray) modeling at false discovery rates (FDR) of <0.05 is shown for defining the association between CEACAM1 expression defined by ^159^Tb-labeled antibody staining and the global populations contained within the 5 clinical subtypes including PBMC from healthy donors (*n* = 5), treatment-naive (*n* = 7) and treatment-resistant (*n* = 3) samples and the tumor cells dissociated from metastatic lesions from treatment-naive (*n* = 9) and treatment-resistant (*n* = 10) melanoma patients. These analyses identified regionalized clusters within the associated limbs of the radial hierarchical trees consistent with B cells, monocytes, dendritic cells, CD4^+^ and CD8^+^ T cells based upon the marker expression in the Citrus clusters as in (Supplementary Fig. 3). This modeling at an FDR < 0.05 was used for investigations as described in the text unless otherwise stated. Nodes examined for disease association are encircled in black.

We characterized the global architecture of each clinical sample type by a visualization tool for high-dimensional single-cell data based upon the t-distributed stochastic neighbor embedding (viSNE) algorithm^23^ (Fig. 1d). Sequential gating allowed the assignment of the cell types within the viSNE map of the 5 clinical subsets (Fig. 1e, f) as defined in Supplementary Table 4. CEACAM1, PD1, and PD-L1 expression were observed at variable levels on all the major immune cell types detected in the tumor-associated PBMC and/or tumor tissue (Fig. 1g, Supplementary Fig. 1h–j). Quantitation of the major cell types contained in the tumor samples also showed that the relative levels of B cells were increased in the treatment-naive relative to treatment-resistant tumors; the relative levels of CD8^+^ T cells were on the other hand increased in the treatment-resistant relative to treatment-naive tumors (Fig. 1h). There was, however, a notable paucity of natural-killer (NK) and innate T cells in the tumor samples (Fig. 1e, f, h), potentially due to down-regulation of cell surface marker expression such that they eluded detection by the panel of antibodies used^24^. Nonetheless, the circulating levels of NK and innate T cells were significantly increased in PBMC of the tumor patients relative to the associated tumors (Fig. 1h). After re-clustering (Fig. 2a–c), bivariant, dual marker density plots showed that all the PD1-bearing NK cells co-expressed CEACAM1 in the setting of treatment resistance (Fig. 2d). Similarly, after re-clustering (Fig. 2e–g), all PD1-bearing innate T cells in the peripheral blood of patients with treatment resistance were also shown to co-express CEACAM1 (Fig. 2h).

To further investigate the nature of the NK and innate T cell subsets that express CEACAM1 and PD1 in the peripheral blood, we next performed FlowSOM analysis. FlowSOM conducts a self-organizing map (SOM) to cluster cells based upon the expression of distinct features and provides structure to the cellular relationships in the form of nodes (metaclusters) in association with a minimal spanning tree (MST)^25^. Using this algorithm, we structured the concatenated NK cells from the peripheral blood of all three clinical samples (HD, PBMC-N, PBMC-R) into an MST (Supplementary Fig. 2a). This showed CEACAM1 marked a metacluster (M5), and to a lesser extent M3 (Supplementary Fig. 2b), that co-expressed PD1 (Fig. 2i, Supplementary Fig. 2c) and was annotated as CD16^+^CD57^+^CD38^+^CD11c^+^CD56^+^CD45RA^+^ in the treatment-naive and -resistant samples (Supplementary Fig. 2d). A similar analysis of the innate T cells showed CEACAM1 marked a metacluster (M1) (Supplementary Fig. 2e, f) that co-expressed PD1 (Fig. 2j) in the circulation of treatment-naive and -resistant patients (Supplementary Fig. 2g) and was annotated as CCR6^+^CD8a^+^CD16^+^CD45RO^+^CD45RA^+^CD161^+^CD27^+^CD28^+^CD3^+^CD127^+^ (Supplementary Fig. 2h). These studies suggest that CEACAM1 expression marks distinct types of circulating innate cells in association with treatment resistance.

To determine whether the expression of CEACAM1 was also significantly different between the cell types in the tumors, we used cluster identification, characterization, and regression (Citrus)^26^ analysis of the global populations together with an assessment by Significance Analysis of Microarrays (SAM) set at different false discovery rates (FDR, 0.01–0.1). The radial hierarchical trees produced by this analysis aligned with each other (Fig. 2k, Supplementary Fig. 3a, b). They showed that significant differences in CEACAM1 expression levels could also be detected on subsets of B cells, monocytes, dendritic cells (DC), CD4 T cells, and CD8 T cells suggesting an association between CEACAM1 on multiple cell types and the clinical phenotypes tested (Fig. 2k, Supplementary Fig. 3a–c). We therefore next focused on a more detailed examination of the specific characteristics of the CEACAM1-expressing immune cells in the tumors and their association with therapeutic exposure.

### Multiple stages of tumor-associated differentiated B cells express increased levels of CEACAM1

**Fig. 3:**
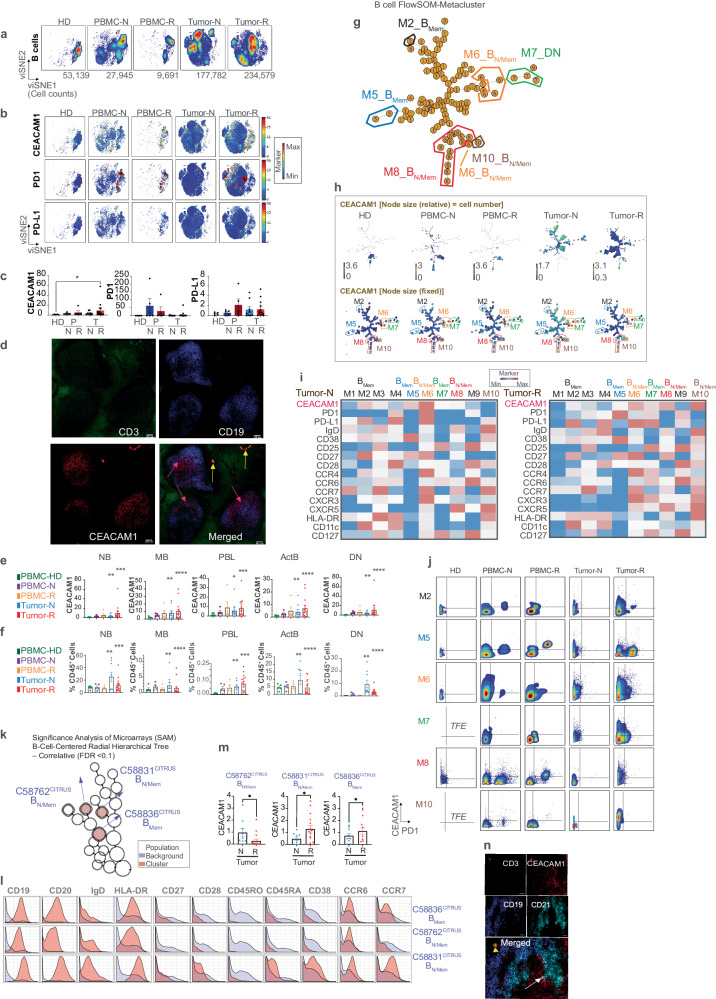
Characterization of CEACAM1 expression on B cells. **a** viSNE visualization of the cellular re-clustering of proportionally exported B cells as indicated for each clinical sample. The cell numbers associated with each type of clinical sample are indicated in the bottom of the representative viSNE plot associated with each group. Peripheral blood mononuclear cells (PBMC) of healthy donors (HD, *n* = 5), PBMC from treatment-naive melanoma patients (PBMC-N, *n* = 7) and treatment-resistant (PBMC-R, *n* = 3) melanoma patients and dissociated tumor cells from treatment-naive (Tumor-N, *n* = 9) and treatment-resistant (Tumor-R, *n* = 10) melanoma patients; **b** The location in viSNE space of median CEACAM1 (median value of ^159^Tb) expression levels compared to PD1 (median value of ^169^Tm) and PD-L1 (median value of ^175^Lu) across all re-clustered B cells as in (**a**) is shown. The color-coded scale bar showing minimum (blue) and maximum (red) is on right. **c** Bar graphs showing CEACAM1, PD1, and PD-L1 expression levels as in (**b**). *p < 0.05 significance by Kruskal–Wallis test, followed by Dunn’s multiple comparison test, was used to compare expression levels of markers on single cells in five unmatched groups; HD, healthy donor. PBMC (P) and tumor (T) from naive (N) and resistant (R) samples as in (**a**), error bar on graphs were plotted with standard error of the mean acquisition; **d** Confocal microscopy staining with CD3 (green), CD19 (blue) or CEACAM1 using 26H7 monoclonal antibody (red) of human tonsil. Merged image is shown on the right with co-expression of CEACAM1 on B (pink arrows) and T (yellow arrows) cells. Size bars = 100 µm; **e** Quantitation of CEACAM1 expression in manually gated B cell sub-populations. NB naive B cell. MB memory B cell. PBL plasmablast. Act activated B cell. DN double-negative. *p < 0.05; **p < 0.01; ***p < 0.001; ****p < 0.0001 significance using a one-sample Wilcoxon signed rank test, error bar on graphs were plotted with standard error of the mean acquisition; **f**. Quantitation of B cell subsets as in (**e**), as proportion of CD45^+^ cells. **p < 0.01; ***p < 0.001; ****p < 0.0001 significance using a one-sample Wilcoxon signed rank test, error bar on graphs were plotted with standard error of the mean acquisition; **g** Minimal spanning tree (MST) of FlowSOM analysis of merged B cell samples. The location and phenotype of the CEACAM1-expressing metaclusters (M) are highlighted by a colored border as characterized in (**h** and Supplementary Fig. 4c). N/Mem, naive-memory, DN, double-negative; **h** Median CEACAM1 expression levels within the MST (**g**) shown as relative proportions in each node (top) or as fixed cluster sizing (bottom) in samples as defined in (**a**). The color-coded scale depicting the median levels of CEACAM1 expression for each sample type is indicated with minimum (blue) and maximum (red); **i** Heatmap of relative expression levels of specific markers in the treatment-naive (N) and -resistant (R) tumors of metaclusters (meta). Scale bar of expression (blue, minimum; red, maximum) is shown; **j** Bivariant, dual marker density plots displaying CEACAM1 and PD1 expression in metaclusters (M) associated with the clinical subtypes as in (**a**). TFE, Too Few Events. **k** CEACAM- expressing nodes identified by Citrus using significance analysis of microarrays (SAM) modeling annotated as different types of B cells extracted from Supplementary Fig. 3a at a False Discovery Rate (FDR) < 0.1. The CEACAM1-expressing nodes are filled red and their Citrus (C) annotation indicated; **l** Expression of selected markers as histograms of Citrus (C) metaclusters C58836, C58762, and C58831 and their annotation, based upon this expression, is indicated on the right; **m** Quantitation of CEACAM1 expression levels in the naive (N) and resistant (R) tumor samples of Citrus nodes in (**k**) and their differences determined by Kruskal–Wallis test, followed by the Dunn’s multiple comparison test for metacluster C58762 or a two-tailed paired *t*-test for metaclusters C58831 and C58836. *p < 0.05, error bars on graphs were plotted with standard error of the mean acquisition; **n** Confocal microscopy of metastatic melanoma lesion stained with CD3 (green), CD19 (blue), human (h)CEACAM1 (red) and CD21 (cyan) with a merged image showing overlap of hCEACAM1 and CD3 (yellow arrowhead) or CD19, CD21, and hCEACAM1 (white arrow). Size bars = 50 μm. Representative of 2 patients.

We initially investigated B cells which were a proportionally dominant group of cells in the tumors and increased in the treatment-naive relative to treatment-resistant samples (Fig. 1e, f, h). To understand the B cell complexity within each clinical sample, we concatenated the CD45^+^CD19^+^ B cells from each study participant in the 5 groups of patients and proportionally exported and re-clustered a total of 503,136 B cells from which a viSNE map was generated (Fig. 3a). A global assessment of CEACAM1, PD1, and PD-L1 was conducted on the exported and re-clustered B cells which identified cell populations that expressed these markers in diseased (tumor and PBMC)-associated samples of both treatment-naive and -resistant patients but not healthy donors (Fig. 3b). We quantified this expression and compared the 5 types of clinical samples. This showed that the highest CEACAM1 levels were observed on B cells in the treatment-resistant tumors (Fig. 3c). Although CEACAM1 was not detected at significant levels in the healthy donor PBMC (Fig. 3b, c), expression was observed on healthy follicle-associated B and T cells in tonsil by confocal microscopy (Fig. 3d), suggesting that CEACAM1 is associated with antigen-experienced B cells.

To define the B cell phenotypes that expressed elevated CEACAM1 levels in relation to tumor-associated disease progression, we first used manual gating strategies to parse the viSNE coordinates into naive (NB; CD19^+^IgD^+^CD27^−^), memory (MB; CD19^+^CD27^+^), plasmablastic (PBL; CD27^+^CD38^+^CD20^−^), activated (Act; IgD^+^CD38^+^) and double-negative (DN; IgD^−^CD27^−^) subsets and identified these populations within the viSNE coordinates (Supplementary Fig. 4a). This showed that the DN, activated and memory populations overlapped with the naive-like, perhaps naive-memory, pool of cells (Supplementary Fig. 4a). CEACAM1 expression was detected at increased levels on all types of B cells with the highest levels in treatment-resistant disease (Fig. 3e, Supplementary Fig. 4b). In comparison, the relative proportions of these same B cell subsets were significantly decreased in the treatment-resistant relative to treatment-naive tumor samples (Fig. 3f), as observed for the global B cell populations (Fig. 1h). These studies suggest that CEACAM1 expression may inversely correlate with the levels of these B cell subtypes in treatment-resistant disease.

To further investigate the B cell subsets expressing CEACAM1, we next employed FlowSOM analysis. Using this algorithm, we structured the concatenated B cells into an MST (Fig. 3g). This showed that CEACAM1 in the tumor samples was associated with metaclusters which were annotated as memory (M2, M5), naive-memory (M6, M8, M10; CD19^+^IgD^+^CD27^+^) and DN (M7) B cells in the treatment-naive and -resistant samples based upon the channel-colored marker levels in the MST (Fig. 3h, Supplementary Fig. 4c), the scaled marker expression in a heatmap (Fig. 3i) and an overlay of the MST and manually gated output (Supplementary Fig. 4d). We also confirmed that the CEACAM1^+^, FlowSOM-associated metaclusters mapped to the CEACAM1-expressing viSNE coordinates (Supplementary Fig. 4e). Bivariant, dual marker density plots showed that many of these B cell metaclusters in the tumors were predominantly characterized by CEACAM1 expression alone (Fig. 3j). However, several of the memory- (M5) and naive/memory-like (M8) metacluster-associated B cells were characterized by CEACAM1 and PD1, but not PD-L1, co-expression in the circulation of the treatment-resistant samples (Fig. 3j, Supplementary Fig. 4f).

Our studies suggested CEACAM1 is expressed at multiple stages of B cell differentiation in the TME and the associated circulating cells. We next sought to determine whether CEACAM1 expression was associated with disease progression as suggested by manual gating and FlowSOM analysis. Our Citrus analysis (Fig. 2k, Supplementary Fig. 3) detected a family of 3 CEACAM1^+^ B cell metaclusters (C58762, C58831, C58836) (Fig. 3k) that represented different types of CD19^+^CD20^+^HLA-DR^+^CCR6^+^CD27^−^ memory-like cells that expressed variable levels of IgD, CD45RA, CD38 and CCR7 (Fig. 3l)^27^. Further, we exported and analyzed the median CEACAM1 expression levels on these three metaclusters and found the correlations detected by SAM were due to significantly increased CEACAM1 expression in the treatment-resistant tumor samples in the cases of C58831 and C58836 or treatment-naive samples in the case of C58762 (Fig. 3m). Confocal microscopy confirmed CEACAM1 expression on B cells from metastatic melanoma lesions in conjunction with CD21 that is expressed by memory B cells (Fig. 3n). These studies show both treatment-naive and -resistant conditions are associated with CEACAM1 expression on memory-related B cells in the tumors and peripheral blood.

### CEACAM1 is expressed on distinct monocytic cell types in the peripheral blood and TME of melanoma

**Fig. 4:**
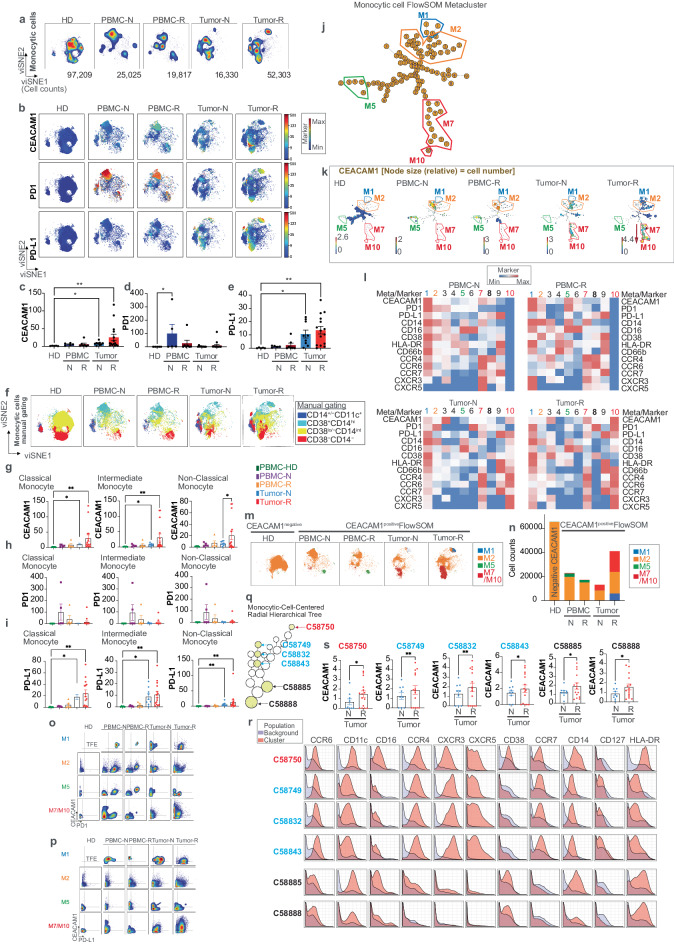
Characterization of CEACAM1 expression on monocytic cells. **a** viSNE visualization of the cellular re-clustering of proportionally exported monocytic cells from peripheral blood mononuclear cells (PBMC) of healthy donors (HD, *n* = 5), treatment-naive (PBMC-N, *n* = 7) and treatment-resistant (PBMC-R, *n* = 3) or treatment-naive tumors (Tumor-N, *n* = 9) and treatment-resistant tumors (Tumor-R, *n* = 10). The cell numbers associated with each type of clinical sample are indicated at the bottom of the representative viSNE plots associated with each group; **b** The location in viSNE space of median CEACAM1 expression (median value of ^159^Tb) compared to PD1 (median value of ^169^Tm) and PD-L1 (median value of ^175^Lu) across all re-clustered monocytic cells are shown. The color-coded scale bar showing minimum (blue) and maximum (red) is shown on right; **c**–**e** Bar graphs showing CEACAM1 (**c**), PD1 (**d**), and PD-L1 (**e**) expression levels as in (**b**). *p < 0.05; **p < 0.01 significance by Kruskal–Wallis test, followed by the Dunn’s multiple comparison test, error bar on graphs were plotted with standard error of the mean acquisition; **f** Location of manually gated populations of classical (CD38^+^CD14^hi^), transitional/intermediate (CD38^lo/^^−^CD14^hi^), non-classical (CD38^−^CD14^−^) and non-classified (CD14^+/^^−^CD11c^+^) monocytic cells within the viSNE space of clinical samples as defined in (**a**); **g**–**i** Bar graphs showing CEACAM1 (**g**), PD1 (**h**), and PD-L1 (**i**) expression levels in manually gated subsets of monocytic cells as in (**f**) associated with sample types as in (**a**). *p < 0.05, **p < 0.01 significance by Kruskal–Wallis test, followed by the Dunn’s multiple comparison test, except for CEACAM1 expression in non-classical monocytes in which the differences between the treatment-naive and -resistant tumor samples were determined by using a paired *t*-test with two tails. Error bars on graphs were plotted with standard error of the mean acquisition; **j** Minimal spanning tree (MST) of FlowSOM analysis of merged monocytic cell samples. The location and phenotype of the CEACAM1-expressing metaclusters are highlighted as characterized in (**k**); **k** Median CEACAM1 expression within the MST (**j**) with the node size relative to the cell number in samples as defined in (**a**). The color-coded scale depicting the median CEACAM1 expression levels for each sample type is indicated with minimum (blue) and maximum (red). The CEACAM1-expressing metaclusters (M) are indicated. Arrow indicates the proposed direction of cell differentiation; **l** Heatmap showing scaled expression of selected markers associated with metaclusters (meta) in treatment-naive (N) and -resistant (R) PBMC or tumor samples. Each metacluster node is color-coded according to (**j**, **k**). The minimum (blue) and maximum (red) color-coded scale is indicated; **m** Visualization of FlowSOM metaclusters that express CEACAM1 as in (**k**) within the viSNE coordinates of the clinical samples as in (**a**) is shown; **n** Absolute cell counts of FlowSOM metaclusters as in (**m**) in each clinical sample type as in (**a**); **o**, **p** Bivariant, dual marker density plots of CEACAM1 and PD1 (**o**) or CEACAM1 and PD-L1 (**p**) expression in monocytic metaclusters M1, M2, M5, and M7/M10 in the clinical samples as in (**a**); **q** CEACAM1-expressing nodes identified by Citrus using significance analysis of microarrays (SAM) modeling annotated as different types of monocytic cells extracted from Fig. 2k. The CEACAM1-expressing nodes are filled yellow. The two types of parent-daughter node relationships are indicated by blue or red; **r** Expression of selected markers as histograms on Citrus metaclusters identified in (**q**); **s** Quantitation of the levels of CEACAM1 expression in the naive (N, *n* = 9) and resistant (R, *n* = 10) tumor samples of Citrus nodes in (**q**) associated with Citrus metaclusters indicated whose differences were determined by a two-tailed paired *t*-test. *p < 0.05 and **p < 0.01 significance, error bar on graphs were plotted with standard error of the mean acquisition.

A similar analysis of 210,684 CD45^+^CD14^+^ monocytic cells was next performed (Fig. 4a). Although CEACAM1 was not detected in the healthy donors, it was observed in the tumor-associated PBMC, in conjunction with the display of PD1, and in the tumors where it localized to viSNE islands that also exhibited PD1 and PD-L1 expression (Fig. 4b). Further, the highest CEACAM1 and PD-L1 levels were observed in the treatment-resistant tumors; CEACAM1 and PD-L1 levels were also elevated on the treatment-naive tumor-associated monocytic cells relative to the healthy donors (Fig. 4c, e). PD1 was most significantly increased on the monocytic cells in the treatment-naive tumor-associated PBMC (Fig. 4d). We used manual gating to parse the monocytic cells into classical (CD38^+^CD14^hi^), transitional/intermediate (CD38^lo/^^−^CD14^int^) and non-classical (CD38^−^CD14^−^) cell types (Supplementary Fig. 5a) and found that the tumor-associated islands mapped to distinct viSNE coordinates (Fig. 4f). Further, all three classes of monocytic cells contributed to the high levels of CEACAM1 and PD-L1 expression observed in the treatment-naive and -resistant TME (Fig. 4g–i). Together, these studies reveal overlapping CEACAM1, PD1, and PD-L1 expression in the tumor-associated samples.

To understand the nature of the monocytic cells that express CEACAM1 within the TME, we performed FlowSOM analysis and generated an MST of this output (Fig. 4j). An examination of the channel-colored marker expression in the MST (Fig. 4k) and heatmap of the scaled marker expression (Fig. 4l), showed the highest CEACAM1 levels were detected on a family of nodes present on a limb of the MST that coincided with two sequential metaclusters (M7, M10) (Fig. 4k). M7/M10 mapped to viSNE coordinates distinct to the TME (Fig. 4m, n). Overlay of the manual gating and the MST outputs showed that whereas the proximal nodes of the M7/M10-associated limb were primarily characterized as classical cells, the distal limb-associated nodes were mostly derived from intermediate and non-classical monocytic cell types (Supplementary Fig. 5b). Examination of the channel-colored levels of marker expression within the MST along the proximal-to-distal trajectory of the M7/M10-associated MST limb showed a progressive decrease in expression of CD14, CD16, CD38, and CD127 expression, consistent with the transition from classical to non-classical cell types. An increase of CXCR3, CXCR5, CCR7, PD-L1 and PD1 suggested increasing dysfunction (Supplementary Fig. 5c)^28–30^. Bivariant, dual marker density plots confirmed metacluster M7/M10 consisted of CEACAM1^+^PD1^+^ (Fig. 4o) and CEACAM1^+^PD-L1^+^ (Fig. 4p) cells in the tumors. FlowSOM also detected a small collection of cells (metacluster M1) that was also unique to the TME especially within the treatment-resistant samples (Fig. 4m, n), consisted of mostly classical monocytic cells (Supplementary Fig. 5b) and expressed CEACAM1 together with PD1 and PD-L1 (Fig. 4l, o, p, Supplementary Fig. 5c). These properties suggest that metacluster M1 represents another collection of cells that are unique to the TME and marked by CEACAM1.

FlowSOM output also focused our attention on two metaclusters (M2, M5) that mapped to distinct viSNE coordinates in the tumor-associated PBMC relative to the healthy donors or tumor samples (Fig. 4m, n) and represented classical (M2) or intermediate/non-classical (M5) monocytic cells (Supplementary Fig. 5b) which were marked by CEACAM1 and PD1 co-expression even in the treatment-naive samples (Fig. 4l, o). This suggested that the elevated levels of PD1 observed on circulating tumor-associated PBMC (Fig. 4d) may reflect expression on metaclusters M2- and M5-associated cells and in association with CEACAM1. These studies suggest CEACAM1 marks distinct monocytic cell states that are enriched within the blood or tumor of melanoma patients and possess phenotypic evidence of immunoregulatory activity.

The portrait obtained from Citrus using SAM modeling of the global cell content also identified CEACAM1-bearing nodes consistent with monocytic cells within the radial hierarchical tree (Figs. 2k and 4q, Supplementary Fig. 3). This analysis identified two CEACAM1^+^ parent nodes and associated branches based upon their marker expression (Fig. 4q, r). This included Citrus parent metacluster C58888 (CD14^lo^CD38^lo^CD16^lo^) and phenotypically similar daughter metaclusters (C58843, C58832, C58749) that upregulated CCR6, CCR4, CXCR3, CXCR5 and CCR7 or a parent metacluster C58885 (CD14^+^CD38^+^CD16^+^). A phenotypically similar daughter node (C58750) likewise upregulated these same chemokine receptors. This suggested that CEACAM1 expression on classical/intermediate (C58885) and intermediate/non-classical (C58888) monocytic cells is associated with cellular dysfunction^31–34^. Interestingly, these phenotypic characteristics identified by Citrus were like the tumor-associated metaclusters (M1, M7, M10) defined by FlowSOM which were enriched within the TME (Fig. 4l–n, Supplementary Fig. 5c). Further, the median CEACAM1 expression levels on all these Citrus metaclusters were significantly increased in the treatment-resistant, relative to treatment-naive, tumor samples (Fig. 4s). Citrus analysis thus confirmed CEACAM1 expression in treatment-naive and -resistant tumor tissue with the highest levels in the latter.

### CEACAM1 expression is associated with distinct types of dendritic cells in the TME

**Fig. 5:**
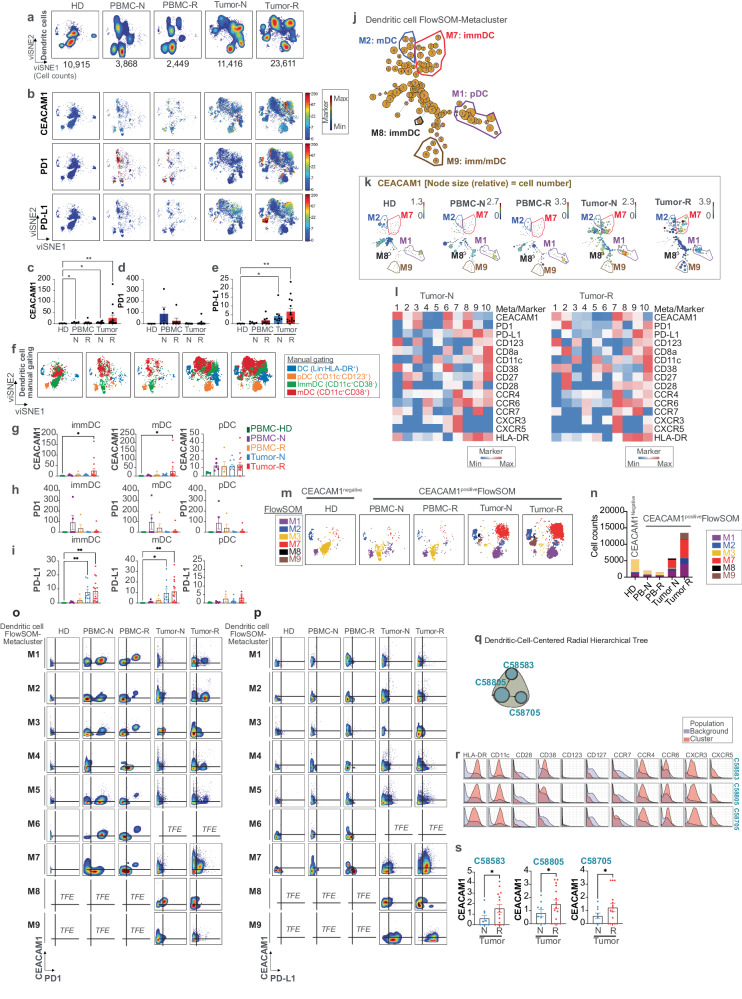
Characterization of CEACAM1 expression on dendritic cells. **a** viSNE visualization of the cellular re-clustering of proportionally exported dendritic cells (DC) from peripheral blood mononuclear cells (PBMC) of healthy donors (HD, *n* = 5), treatment-naive (PBMC-N, *n* = 7) and treatment-resistant (PBMC-R, *n* = 3) or treatment-naive tumors (Tumor-N, *n* = 9) and treatment-resistant tumors (Tumor-R, *n* = 10). The cell numbers associated with each type of clinical sample are indicated in the bottom of the representative viSNE plot associated with each group; **b** The location in viSNE space of median levels of CEACAM1 expression (median value of ^159^Tb) compared to PD1 (median value of ^169^Tm) and PD-L1 (median value of ^175^Lu) across all re-clustered DC is shown. The color-coded scale bar and range is shown (blue, minimum; red, maximum); **c**–**e** Bar graphs showing quantification of CEACAM1 (**c**), PD1 (**d**), and PD-L1 (**e**) expression as in (**b**). *p < 0.05; **p < 0.01 significance by Kruskal–Wallis test, followed by the Dunn’s multiple comparison test, error bar on graphs were plotted with standard error of the mean acquisition; **f** The re-clustered cells were defined by manual gating within the landscape of the viSNE coordinates as shown by the legend on the right (DC non-assigned dendritic cell, pDC plasmacytoid DC, immDC immature DC, mDC mature DC); **g**–**i** Quantification of CEACAM1 (**g**), PD1 (**h**), and PD-L1 (**i**) expression on manually gated immature (imm), mature (m), and plasmacytoid (p) dendritic cells (DC) as defined in (**f**) and in the 5 clinical types of samples as in (**a**). *p < 0.05 and **p < 0.01 significance by Kruskal–Wallis test, followed by the Dunn’s multiple comparison test, error bar on graphs were plotted with standard error of the mean acquisition; **j** Minimal spanning tree (MST) of FlowSOM analysis of DC in merged cell samples. The location and phenotype of the CEACAM1-expressing metaclusters (M) are highlighted as characterized in (**k**); **k** Median CEACAM1 expression within the MST (**j**) shown as scaled numbers in each node with a size relative to cell number. The color-coded scale depicting the levels of CEACAM1 expression for each sample type as in (**a**) is indicated as blue (minimum) or red (maximum); **l** Heatmap showing scaled expression of selected markers associated with metaclusters (meta) as in (**j**) in treatment-naive (N) and -resistant (R) tumor samples. The minimum (blue) and maximum (red) color-coded scale is indicated; **m** Location of each metacluster identified in (**j**, **k**) within the viSNE space and samples as in (**a**). The annotation of each metacluster is indicated by the colored code as in (**j**); **n** Absolute cell counts of FlowSOM metaclusters as in (**m**) in each clinical sample type as in (**a**); **o**, **p** Bivariant, dual marker density plots of CEACAM1 and PD1 (**o**) or CEACAM1 and PD-L1 (**p**) expression in DC-associated metaclusters (M) as in (**j**). TFE, too few events; **q** CEACAM1-expressing nodes identified as DC (blue-filled circles) by significance analysis of microarrays modeling contained in the Citrus-associated radial hierarchical tree extracted from Fig. 2k; **r** Expression of selected markers as histograms associated with Citrus (C) metaclusters identified in (**q**); **s** Quantitation of the levels of CEACAM1 expression in the naive (N, *n* = 9) and resistant (R, *n* = 10) tumor samples of Citrus nodes in (**q**, **r**) associated with Citrus metacluster C58583, C58805, and C58705, and their differences determined by a two-tailed paired t-test. *p < 0.05 significance, error bar on graphs were plotted with standard error of the mean acquisition.

In a similar manner, we exported and scrutinized 52,259 Lin^−^HLA-DR^+^ dendritic cells (DC). This showed that the re-clustered cells distributed to distinct locations within the viSNE coordinates within the TME compared to those contained in PBMC (Fig. 5a). We overlaid CEACAM1, PD1, and PD-L1 expression on the DC-associated viSNE coordinates and found that these were expressed and mapped together in several of the distinct tumor-associated islands (Fig. 5b). These three markers were absent from the healthy donor controls but expressed at low levels on the tumor-associated PBMC (Fig. 5b). CEACAM1 and PD-L1 levels were increased in the treatment-naive and -resistant tumor samples relative to that observed in PBMC (Fig. 5c, e); the levels of PD1 expression were also increased in the tumor-associated PBMC, although not significantly (Fig. 5d), as was CEACAM1 (Fig. 5c). Manual gating was used to parse the DC into immature (imm) (CD11c^+^CD38^−^), mature (m) (CD11c^+^CD38^+^) and plasmacytoid (p) (CD11c^−^CD123^+^) fractions. The relative levels of these DC subsets were not significantly different between the 5 types of clinical samples (Supplementary Fig. 6a). Although all three DC subsets contributed to the tumor-associated islands, they mapped to distinct locations within the viSNE space relative to the locations observed in the PBMC samples suggesting they were in unique states of differentiation (Fig. 5f). Consistent with this, the levels of CEACAM1 (Fig. 5g) were significantly increased on the immDC and mDC in the treatment-resistant tumors; PD-L1 was increased on both treatment-naive and -resistant tumor groups (Fig. 5i). PD1 levels on these DC subsets were, however, elevated although not significantly in the tumor-associated PBMCs (Fig. 5h). Thus, CEACAM1 and PD-L1 were upregulated on tumor-associated DC with the highest levels found in treatment resistance.

Fine mapping using FlowSOM and its associated MST was used to more precisely define the DC subsets that expressed CEACAM1 (Fig. 5j). The channel-colored expression levels (Fig. 5k), heatmap of the scaled expression (Fig. 5l) and visualization of the FlowSOM-generated metaclusters on the viSNE coordinates (Fig. 5m) identified a CEACAM1-expressing metacluster (M7) as being distinctively increased and a significant contributor to the DC in the TME (Fig. 5n). An overlay of the manual gating on the MST output further showed that metacluster M7 represented a collection of immature and mature DC (Supplementary Fig. 6b) which expressed PD1, PD-L1 and a variety of chemokine receptors including CCR7, CXCR3, CXCR5, CCR4, and CCR6 together suggesting they were DC possessing immunoregulatory properties (Fig. 5l)^35–38^. Bivariant, dual marker density plots confirmed CEACAM1, PD1, and PD-L1 co-expression on cells associated with metacluster M7 in the treatment-resistant tumors and together with PD1 in the treatment-resistant PBMC (Fig. 5o, p). Deep clustering of the FlowSOM output showed that approximately 50% of the M7-associated clusters (C61, C62, C71, C73, C81, C82, C84, C91, C92, C93) were triple-positive cells in the tumors (Supplementary Fig. 6c, d). FlowSOM also detected 2 minor metaclusters (M8, M9) of mostly immature DC (Supplementary Fig. 6b) that expressed CEACAM1, PD1 and/or PD-L1 (Fig. 5l, o, p) which were restricted to the TME (Fig. 5m, n). Thus, CEACAM1 marked collections of DC with phenotypic characteristics of immunoregulatory cells based upon their co-expression of PD1 and/or PD-L1 which were unique to the TME.

Citrus modeling (Fig. 2k, Supplementary Fig. 3) also identified three metaclusters (C58583, C58805, C58705) that congregated together within a distinct limb of the radial hierarchical tree (Fig. 5q), which represented a group of HLA-DR^+^CD11c^+^CCR4^+^CCR6^+^CXCR3^+^CXCR5^+^ cells that were CD38^−^ (C58705), CD38^lo^ (C58805) or CD38^+^ (C58583), consistent with immature or mature, monocyte-derived DC (Fig. 5r). CEACAM1 expression in each of these metaclusters was assessed for each clinical phenotype. This showed CEACAM1 expression in the treatment-naive and -resistant samples with the highest levels in the latter in each node identified by Citrus (Fig. 5s). CEACAM1 thus marked unique types of immature and mature DC that were expanded within the tumor tissues and overlapped with PD1 and PD-L1 expression; the highest levels of CEACAM1 expression were present in treatment-resistant disease.

### CEACAM1 is expressed on follicular helper CD4^+^ T cells and regulatory CD4^+^ T cells in the TME

**Fig. 6:**
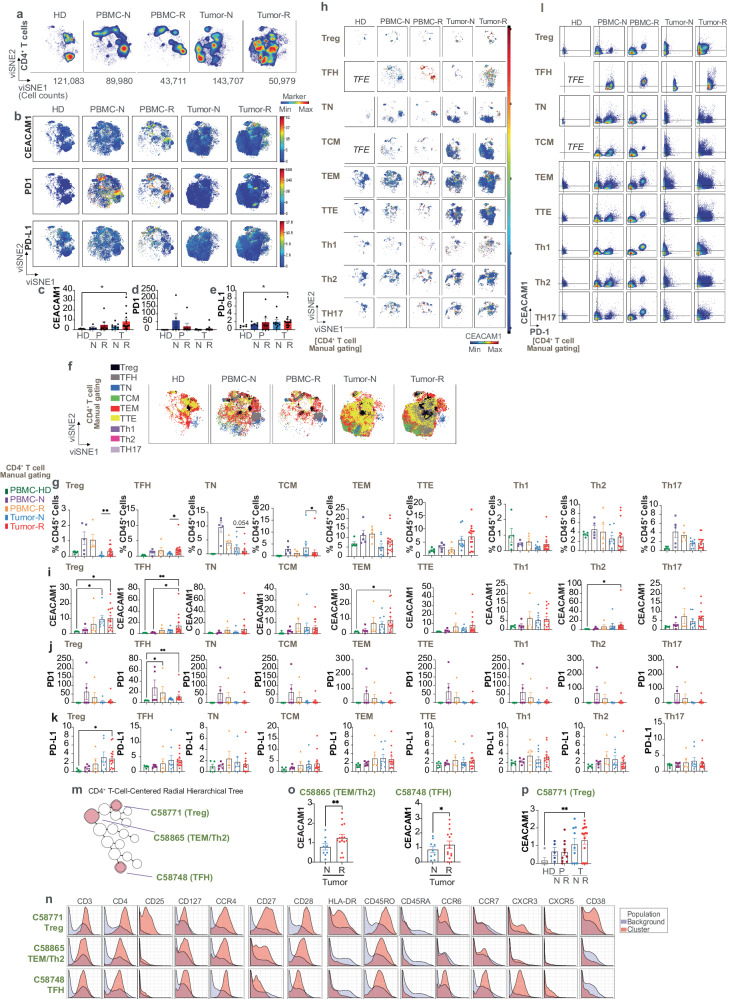
Phenotypic analysis of CEACAM1 on CD4^+^ T cells. **a** viSNE visualization of the cellular re-clustering of proportionally exported CD4^+^ T cells from peripheral blood mononuclear cells (PBMC) of healthy donors (HD, *n* = 5), treatment-naive (PBMC-N, *n* = 7) and treatment-resistant (PBMC-R, *n* = 3) or treatment-naive tumors (Tumor-N, *n* = 9) and treatment-resistant tumors (Tumor-R, *n* = 10). The cell numbers associated with each type of clinical sample are indicated in the bottom of the representative viSNE plot associated with each group; **b** The location in viSNE space of scaled median CEACAM1 expression (median value of ^159^Tb) compared to PD1 (median value of ^169^Tm) and PD-L1 (median value of ^175^Lu) across all re-clustered CD4^+^ T cells are shown for each clinical group as in (**a**). The color-coded scale bar and range is shown (blue, minimum; red, maximum); **c**–**e** Bar graphs showing CEACAM1 (**c**), PD1 (**d**), and PD-L1 (**e**) expression levels. HD healthy donor, P PBMC, T tumor, N naive, R resistant as in (**a**). *p < 0.05 significance by Kruskal–Wallis test, followed by Dunn’s multiple comparison test, error bar on graphs were plotted with standard error of the mean acquisition; **f** The re-clustered CD4^+^ T cells were defined as either regulatory T cells (Treg; CCR4^+^CD25^++^CD127^+/^^−^), follicular helper T cells (TFH; CXCR5^+^PD1^+^), naive T cells (TN; CD45RA^+^CD45RO^−^CCR7^+^), central memory T cells (TCM; CD45RA^−^CD45RO^+^CCR7^hi^), effector-memory T cells (TEM; CD45RA^−^CD45RO^+^CCR7^lo/^^−^CD27^+^), terminal-effector T cells (TTE; CD45RA^−^CD45RO^+^CCR7^−^CD27^−^), Type 1 T helper cells (CD45RO^+^CXCR5^−^CCR4^−^CXCR3^+^CCR6^−^), Type 2 T helper cells (CD45RO^+^CXCR5^−^CCR4^+^CCR6^−^CXCR3^−^) and Th17 cells (CD45RO^+^CXCR5^−^CCR4^+^CCR6^+^CXCR3^−^) by manual gating according to the color-coded legend on the left. Samples defined as in (**a**); **g** CD4^+^ T cell subtype as in (**f**) shown as a relative proportion of the total CD45^+^ cells contained within each clinical type of sample as in (**a**). *p < 0.05; **p < 0.01 significance by using a two-tailed paired *t*-test. Error bars on graphs were plotted with the standard error of the mean acquisition. **h** The location in viSNE space of median CEACAM1 expression (median value of ^159^Tb) of each subset of CD4^+^ T cell as in (**f**) and clinical sample type as defined in (**a**). The scale bar and range of CEACAM1 expression are shown (blue, minimum; red, maximum). TFE Too Few Events; **i**–**k** Bar graphs showing quantitation of CEACAM1 (**i**), PD1 (**j**), and PD-L1 (**k**) expression on manually gated CD4^+^ T cell subsets in the 5 clinical sample types as in (**a**). *p < 0.05; **p < 0.01 significance by Kruskal–Wallis test, followed by the Dunn’s multiple comparison test, error bar on graphs were plotted with standard error of the mean acquisition; **l** Bivariant, dual marker density plots of CEACAM1 and PD1 expression in each subtype of CD4^+^ T cell as in (**f**) and clinical sample as in (**a**); **m** CEACAM1-expressing nodes identified as CD4^+^ T cells by Citrus analysis using significance analysis of microarrays modeling is shown by the red filled circles within the radial hierarchical tree shown extracted from Fig. 2k with their Citrus annotation as indicated; **n** Expression of selected markers in CD4^+^ Citrus metaclusters identified in (**m**) and their identity as indicated; **o** Quantitation of the levels of CEACAM1 expression in the naive (N, *n* = 9) and resistant (R, *n* = 10) tumor samples of Citrus nodes in (**m**) associated with Citrus metacluster C58865 and C58748, and their differences were determined by a two-tailed paired *t*-test. *p < 0.05; **p < 0.01 significance, error bar on graphs were plotted with standard error of the mean acquisition; **p** Quantitation of median CEACAM1 expression levels of cells associated with Citrus metacluster C58771 in the 5 clinical sample types. HD healthy donor, P peripheral blood mononuclear cells, T tumor, N naive, R resistant as in (**a**). The differences were determined by two-way analysis of variance (ANOVA). **p < 0.01. Error bars on graphs were plotted with the standard error of the mean acquisition.

We exported and visualized 903,560 CD4^+^ T cells using viSNE which revealed a distinct localization and expansion of the CD4^+^ T cell compartment in the treatment-naive and -resistant tumors relative to that observed in PBMC (Fig. 6a). An overlay of the scaled expression of CEACAM1 on the viSNE map (Fig. 6b) and its quantification showed significantly increased CEACAM1 levels in the treatment-resistant tumor samples relative to expression in healthy donor controls (Fig. 6c). PD1 and PD-L1 were interestingly increased on the circulating PBMC in the tumor-bearing patients (Fig. 6d) and treatment-resistant tumors (Fig. 6e), respectively. To understand the origin of the elevated CEACAM1 expression in the CD4^+^ T cells within the tumors, we assigned the cells within the islands as naive (TN; CD45RA^+^CD45RO^−^CCR7^+^), central memory (TCM; CD45RA^−^CD45RO^+^CCR7^hi^), effector memory (TEM; CD45RA^−^CD45RO^+^CCR7^lo/^^−^CD27^+^), terminal effector (TTE; CD45RA^−^CD45RO^+^CCR7^−^CD27^−^), follicular helper (TFH; CXCR5^+^PD1^+^), regulatory (Treg; CCR4^+^CD25^++^CD127^+/^^−^), type 1 T helper (Th)1 (CD45RO^+^CXCR5^−^CCR4^−^CXCR3^+^CCR6^−^), Th2 (CD45RO^+^CXCR5^−^CCR4^+^CCR6^−^CXCR3^−^) and Th17 (CD45RO^+^CXCR5^−^CCR4^+^CCR6^+^CXCR3^−^) cells by manual gating. This showed that all manually gated subsets of CD4^+^ T cells contributed to the cellular complexity observed in the tumor samples (Fig. 6f), with a significant relative increase of Treg and TFH in the treatment-resistant relative to treatment-naive tumor samples (Fig. 6g); TCM cells were in contrast increased in the treatment-naive samples (Fig. 6g). To define the localization of CEACAM1 expression amongst the CD4^+^ T cell subsets assigned in this manner, we overlaid CEACAM1 expression on the manually gated cell subsets viewed as separate coordinates within the viSNE map which showed variable CEACAM1 expression levels on each type of CD4^+^ T cell within the 5 clinical phenotypes (Fig. 6h). Further, we quantified CEACAM1 expression in each of the manually gated subsets within the 5 types of clinical samples. This showed CEACAM1 was expressed at significantly increased levels on TFH, TEM, and Th2 cells in the treatment-resistant samples and on Treg cells in both treatment-naive and -resistant tumor samples relative to healthy donor (HD) controls (Fig. 6i). We also quantified the levels of PD1 and PD-L1 on these manually gated subsets and interestingly observed increased PD1 on circulating and tumor-associated TFH cells (Fig. 6j) and PD-L1 on tumor-associated Treg cells (Fig. 6k) relative to the healthy donor controls. Notably, bivariant, dual marker density plots also showed that a discrete collection of CEACAM1^+^PD1^+^ cells could be detected in all manually gated subsets of CD4^+^ T cells within the treatment-resistant PBMC and tumor samples (Fig. 6l). These studies show CEACAM1 expression is increased on CD4^+^ Treg, TFH, TEM and Th2 cells in treatment-naive and/or -resistant disease and can be detected with PD1 on a variety of circulating CD4 T cell subsets.

As Citrus modeling with SAM identified nodes of CD4^+^ T cells which expressed significantly increased levels of CEACAM1 (Fig. 2k, Supplementary Fig. 3), we investigated the output of this analysis to pinpoint the potential disease associations. SAM identified 3 CEACAM1^+^CD4^+^ T cell metaclusters in our Citrus modeling (Fig. 6m). Metacluster C58865 was consistent with CD4^+^ Th2-like, TEM cells (CD45RO^+^CCR4^+^CD27^+^CCR7^−^) (Fig. 6n); C58865 expressed significantly increased CEACAM1 levels in the treatment-resistant, relative to treatment-naive, subset (Fig. 6o). Metacluster C58748 represented CD4^+^CD45RO^+^ T cells that expressed CCR6, CCR7, CXCR3 and CXCR5 suggesting TFH cells in melanoma (Fig. 6n) which exhibit elevated CEACAM1 levels in the context of treatment-resistant compared to treatment-naive disease (Fig. 6o). Finally, metacluster C58771 was distinctively CD25^+^CD38^+^CCR4^+^ consistent with CD4^+^ Treg (Fig. 6n). Quantitation of the median levels of CEACAM1 on cells associated with metacluster C58771 revealed significantly elevated levels in the treatment-resistant tumor samples compared to the healthy donor control samples (Fig. 6p). Together, these studies support a role for CEACAM1 in the function of CD4^+^ Treg, TFH and Th2-related cells within the melanoma TME.

**Fig. 7:**
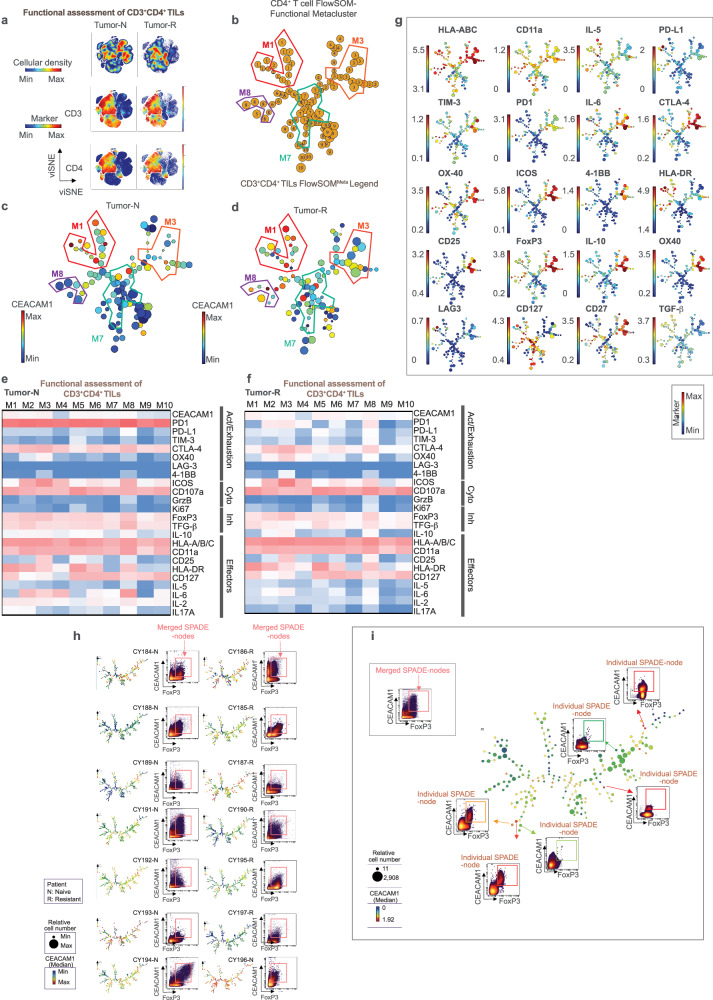
Characterization of CEACAM1 expression relative to functional markers on CD4^+^ T cells. **a** viSNE map of tumor-dissociated cells from treatment-naive (Tumor-N, *n* = 9) and -resistant (Tumor-R, *n* = 5) samples after staining with functional panel of markers. Global population is shown on top. Location of CD3- (middle) and CD4-expressing cells (bottom) are indicated. Scale bars indicating the quantity based upon cellular density and level of marker expression are indicated by the scale bars (blue, minimum; red, maximum). TILs tumor-infiltrating lymphocytes; **b** Legend for minimal spanning tree (MST) from FlowSOM analysis using functional marker panel of exported CD4^+^ T cells from merged groups of Tumor-N and Tumor-R samples as in (**a**). The location of CEACAM1-expressing metaclusters are indicated; **c**–**d** Channel-colored median CEACAM1 expression for treatment-naive (Tumor-N, *N* = 9) (**c**) and treatment-resistant (Tumor-R, *n* = 5) (**d**) samples as in (**b**) shown with fixed cluster sizing. The color-coded scale (blue, minimum; red, maximum) and the location of CEACAM1-expressing metaclusters (M) are indicated; **e, f** Heatmap showed scaled expression of selected markers in metaclusters (M) as defined in (**b**–**d**) in treatment-naive (N) (**e**) and -resistant (R) (**f**) tumors. Act, activation. Cyto, cytotoxic. Inh, inhibitory; **g** Scaled expression of each marker within the channel-colored node whose size is shown as relative proportions which are associated with the MST (**b**) used in the annotation of each CEACAM1-expressing metacluster in (**c**, **d**) with color-coded scale bar indicated for each marker; **h** SPADE analysis of individual patients as indicated associated with treatment-naive (N) and -resistant (R) clinical samples. The scaled channel-colored CEACAM1 expression is indicated for each node (Blue, minimum; Red, maximum). The node sizes are proportional to the cell number as indicated by the scale. Bivariant, dual marker density plots of the global CEACAM1 and FoxP3 expression of the merged SPADE nodes associated with each clinical sample are shown; **i** SPADE analysis of CD4^+^ T cells from a treatment-naive sample (CY191-N) as in (**h**). Bivariant, dual marker density plots showing FoxP3 expression in CEACAM1^high^ (red arrow), CEACAM1^medium^ (orange arrow), and CEACAM1^low^ (green arrow) nodes of SPADE map. The merged expression of CEACAM1 and FoxP3 in the concatenated nodes is indicated. The color-coded scale of CEACAM1 expression (green, minimum; red, maximum). The node sizes are proportional to the cell number as indicated.

Of significant interest was our observation that CEACAM1 marked Treg cells in the TME based upon expression of CD25. To confirm and extend this finding, we also applied an alternative panel of antibodies (panel 2) that allowed for an inventory of other functional cell surface and intracellular markers (Supplementary Table 2) in treatment-naive (*n* = 9) and -resistant (*n* = 5) patients (Supplementary Table 1). We located 7814 CD3^+^CD4^+^-expressing T cells in the viSNE coordinates from those associated with the treatment-naive and -resistant tumors (Fig. 7a). These were exported and examined by FlowSOM to define the cellular complexity. The MST associated with this analysis (Fig. 7b) was examined for CEACAM1 levels as defined by channel-colored levels in each node of the treatment-naive (Fig. 7c) and -resistant (Fig. 7d) tumors. We focused on four CEACAM1^+^ metaclusters (M1, M3, M7, M8) in the treatment-naive and -resistant samples and examined the scaled expression of the markers displayed as heatmaps (Fig. 7e, f) or as channel-colored marker levels associated with the MST nodes (Fig. 7g). These metaclusters exhibited evidence of cell surface (PD1, PD-L1, TIM-3, CTLA-4, ICOS and/or CD25) and intracellular (FoxP3, TGF-β and/or IL-10) marker expression consistent with regulatory activity. Further, we assessed the correlation between CEACAM1 and FoxP3 expression using Spanning-tree Progression Analysis of Density-normalized Events (SPADE) analysis^39^. A merged view of the nodes associated with the SPADE maps of each clinical sample confirmed a correlation between CEACAM1 and FoxP3 expression (Fig. 7h). On closer inspection, we further observed that the CEACAM1^hi^ (red-yellow) nodes were coordinately FoxP3^+^, and the CEACAM1^lo^ (green-blue) nodes FoxP3^−^ (Fig. 7i). These data confirm CEACAM1 expression on FoxP3^+^ T cells, consistent with Treg cells in treatment-naive and -resistant disease.

### CEACAM1 expression on CD8^+^ T cells correlates with resistant disease

**Fig. 8:**
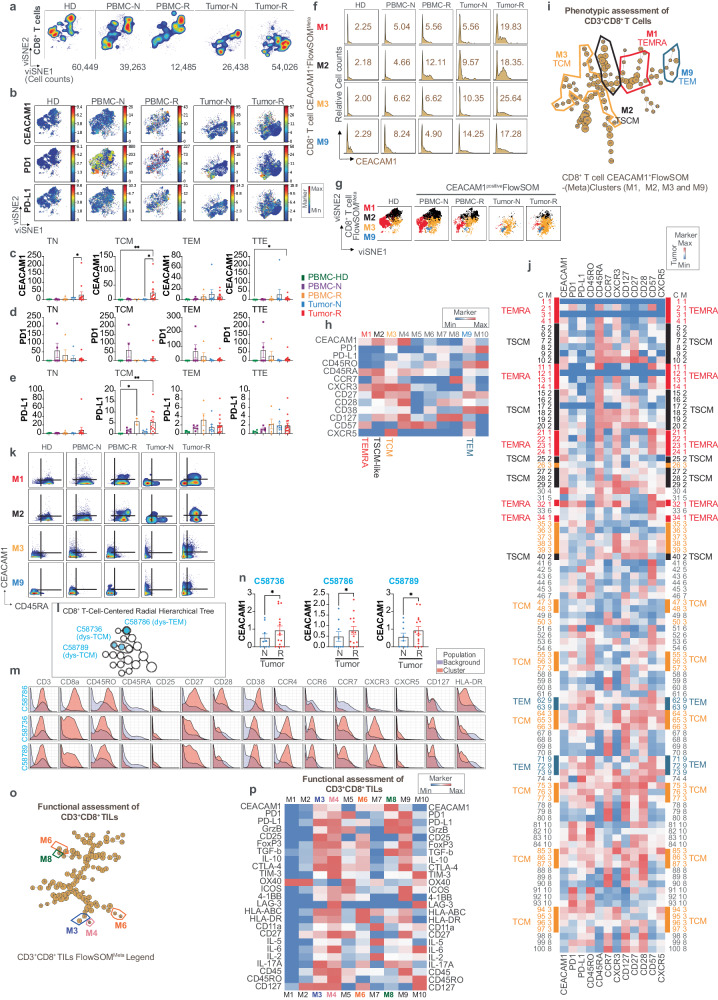
Phenotypic and functional analysis of CEACAM1 on CD8^+^ T cells. **a** viSNE visualization of the cellular re-clustering of proportionally exported CD8^+^ T cells from peripheral blood mononuclear cells (PBMC) of healthy donors (HD, *n* = 5), treatment-naive (PBMC-N, *n* = 7) and treatment-resistant (PBMC-R, *n* = 3) or treatment-naive tumors (Tumor-N, *n* = 9) and treatment-resistant tumors (Tumor-R, *n* = 10). The cell numbers associated with each type of clinical sample are indicated in the bottom of the representative viSNE plot associated with each group; **b** The location in viSNE space of median CEACAM1 expression (median value of ^159^Tb) compared to PD1 (median value of ^169^Tm) and PD-L1 (median value of ^175^Lu) across all re-clustered CD8^+^ T cells are shown for each clinical group as in (**a**). The color-coded scale bar and range are shown (blue, minimum; red, maximum); **c**–**e** The re-clustered CD8^+^ T cells were defined as either naive T cells (TN), central memory (TCM), terminal effector (TTE) or effector memory (TEM) cells by manual gating and quantification of CEACAM1 (**c**), PD1 (**d**) and PD-L1 (**e**) expression is shown according to the clinical sample type as in (**a**) according to the color-coded legend. *p < 0.05; **p < 0.01 significance by Kruskal–Wallis test, followed by the Dunn’s multiple comparison test, error bar on graphs were plotted with standard error of the mean acquisition; **f** Histograms showing CEACAM1 expression in FlowSOM metaclusters associated with each clinical sample type as in (**a**). The median levels of CEACAM1 expression associated with each histogram are indicated; **g** Visualization of FlowSOM metaclusters as in (**f**) on the viSNE coordinates of the clinical samples from (**a**) is shown. The location of each metacluster is indicated by the color-coded legend; **h** Heatmap of scaled expression (blue, minimum; red, maximum) of each marker as indicated on the left margin for each FlowSOM metacluster as indicated on top in the treatment-resistant tumor samples. TEMRA T effector-memory expressing CD45RA, TSCM T stem cell memory, TCM T central memory, TEM T effector memory; **i** Minimal spanning tree for FlowSOM analysis of CD8^+^ T cells from treatment-resistant tumors displayed as individual clusters with the location of CEACAM1-expressing metaclusters (M1, M2, M3, M9) and their annotation indicated. **j** Heatmap showing scaled expression of markers (top) in treatment-resistant samples (Tumor-R) in association with each cluster (C, 1-100) and within each identified metacluster (M, 1-10) of CD8^+^ T cells. The location of relevant clusters is indicated and annotated according to the color code as in (**i**). The expression levels are color-coded as indicated by the scale bar (blue, minimum; red, maximum). C cluster, M metacluster; **k** Bivariant, dual marker density plots showing CEACAM1 and CD45RA expression in association with each metacluster and within each clinical sample as in (**a**); **l** CEACAM1-expressing nodes identified as CD8^+^ T cells by Citrus analysis using significance analysis of microarrays modeling is shown by the blue-filled circles within the radial hierarchical tree shown extracted from Fig. 2k with their Citrus annotation as indicated; **m** Expression of selected markers associated with CD8^+^ T cell metaclusters identified in (**l**); **n** Quantitation of the levels of CEACAM1 expression in the naive (N, *n* = 9) and resistant (R, *n* = 10) tumor samples of Citrus nodes in (**l**, **m**) associated with Citrus metacluster C58736, C58786 and C58789, and their differences were determined by a two-tailed paired *t*-test; *p < 0.05 significance, error bar on graphs were plotted with standard error of the mean acquisition. **o** Legend for the minimal spanning tree from FlowSOM analysis of functional marker panel of exported CD8^+^ T cells of the merged samples from treatment-naive (*n* = 9) and -resistant (*n* = 5) tumors. The location of CEACAM1-positive metaclusters is indicated. TILs tumor-infiltrating lymphocytes; **p** Heatmap of scaled expression (blue, minimum; red, maximum) of functional markers in the FlowSOM-associated metaclusters as in (**o**) in merged treatment-naive and -resistant samples.

We finally exported and re-clustered 192,661 CD8^+^ T cells and observed that naive and resistant tumor-associated tumor cells mapped to distinct viSNE coordinates relative to that observed in the PBMC samples (Fig. 8a), suggesting that those in the tumors possessed unique characteristics. Overlay of the scaled expression of CEACAM1, PD1, and PD-L1 on the viSNE maps of the 5 types of clinical samples showed their expression among the PBMC and tumor, but not healthy donor, samples (Fig. 8b). We performed manual gating and observed significantly elevated CEACAM1 levels in the treatment-resistant relative to treatment-naive tumors in association with CD8^+^ T cells that were phenotypically naive (TN), T central memory (TCM) and terminal-effector (TTE)-related subsets (Fig. 8c). In comparison, PD1 levels on the CD8^+^ T cell subtypes were increased on the circulating cells (Fig. 8d) and PD-L1 levels were increased on the treatment-resistant CD8^+^ TCM-like cells in the tumor and PBMC relative to the healthy donor controls (Fig. 8e). The increased CEACAM1 expression on treatment-resistant, CD8^+^ TN cells was notable, as CEACAM1 is considered an activation antigen on T cells^13,21^.

To more precisely identity the CEACAM1-expressing cells, we applied FlowSOM analysis to the exported CD8-expressing T cells. The channel-colored scaling of CEACAM1 expression showed that CEACAM1 was associated with four metaclusters (M1, M2, M3, M9) (Supplementary Fig. 7a, b). Histograms displaying the median CEACAM1 expression levels confirmed this and showed that the highest levels were present in the treatment-resistant tumor samples (Fig. 8f). Further, the 4 CEACAM1^+^ metaclusters mapped to distinct locations within the viSNE coordinates (Fig. 8g) and represented approximately 22–24% of the tumor-associated CD8^+^ T cells that were mostly derived from metaclusters M3 and M9 with fewer contributions from M1 and M2 (Supplementary Fig. 7c). The nature of the metaclusters was determined by examination of the channel-colored marker expression of the MST-associated nodes (Supplementary Fig. 7a, b) and the heatmap of the scaled marker expression associated with the metaclusters in the treatment-resistant population (Fig. 8h). The major metaclusters were TCM-like (M3; CD45RO^+^PD1^+^CCR7^+^CXCR3^+^CD27^+^CD28^+^CD38^lo^CD127^+^CXCR5^+^CD57^−^) and TEM-like (M9; CD45RO^+^PD1^+^CCR7^−^CXCR3^−^CD27^+^CD28^+^CD38^+^CD127^−^CD57^+^CXCR5^+^) cells that were phenotypically dysfunctional based upon PD1 and CD38 expression^40^. Deep clustering showed that TCM-associated metacluster M3 also expressed PD-L1 (clusters C35, C57, C64, C65, C66, C75, C76, C85, C86, C87, C94, C95, C96) (Fig. 8i, j). In addition, the minor CEACAM1-expressing metaclusters M1 and M2 exhibited CD45RA (Fig. 8h, k, Supplementary Fig. 7a, b). M1 was consistent with TEM-like cells expressing CD45RA (or TEMRA) based upon the presence of CXCR3 and CD57, but the absence of CD45RO, CD28, CCR7, and CD27 (Fig. 8h, Supplementary Fig. 7a, b); TEMRA cells are considered to be terminally differentiated^41^. Deep sub-clustering of the M1-associated TEMRA cells further showed PD1 co-expression with CEACAM1 in a subset (clusters C2, C21, C23, C32) suggestive of terminal exhaustion^41^ (Fig. 8i, j). M2-associated cells were CD45RO^−^CD45RA^+^CCR7^+^CXCR3^+^CD27^+^CD127^+^ consistent with T stem cell-like memory cells, or TSCM^42^ (Fig. 8h Supplementary Fig. 7a, b). Deep clustering of this metacluster found evidence of triple-positive (CEACAM1^+^PD1^+^CXCR5^+^) M2-associated clusters (clusters C7, C10, C15) suggesting precursors of exhausted T cells, or TPEX, within the TSCM pool (Fig. 8i, j).

Citrus modeling of the global population (Fig. 2k, Supplementary Fig. 3) also identified several CEACAM1^+^CD8^+^ T cell metaclusters that were associated with the treatment type (Fig. 8l). This included a single metacluster (C58786), similar to FlowSOM metacluster M9, consistent with dysfunctional TEM-like cells (CD45RO^+^CD27^+^CD28^+^CD38^+^CCR7^−^CXCR3^−^CD127^−^)^43^ (Fig. 8m); the median CEACAM1 expression levels on C58786-associated cells were significantly increased in treatment-resistant disease (Fig. 8n). Similarly, Citrus identified 2 metaclusters (C58736, C58759) (Fig. 8l), like FlowSOM metacluster M3, that were consistent with dysfunctional CD8^+^ TCM-like cells (CD45RO^+^CD27^+^CD28^lo^CD38^+^CCR4^+^CCR6^+^CCR7^+^CXCR3^+^CXCR5^lo^CD127^+^) (Fig. 8m) which also exhibited significantly elevated CEACAM1 levels in the treatment-resistant, relative to treatment-naive samples (Fig. 8n). These results suggest that CEACAM1 expression on CD8^+^ TCM-like and TEM-like cells is highest in treatment-resistant disease.

We also used our panel of functional markers (Supplementary Table 2) on treatment-naive (*n* = 9) and -resistant (*n* = 5) samples (Supplementary Table 1) and followed a similar strategy used for the CD4^+^ T cells. In this way, 180,029 CD8^+^ T cells were identified in the viSNE coordinates (Supplementary Fig. 7d), exported, and parsed into metaclusters using FlowSOM. The MST (Fig. 8o) and accompanying heatmap showing the scaled expression of the markers expressed by these metaclusters in the merged (treatment-naive and -resistant) samples (Fig. 8p) identified several CEACAM1^+^ nodes (M3, M4, M6, M7, M8). Metaclusters M3 and M4 were PD1^+^PD-L1^+^granzyme-B (GrzB)^+^CD25^+^FoxP3^+^TGF-β^+^IL-10^+^CTLA-4^+^TIM-3^+^ICOS^+^4-1BB^+^LAG-3^−^^/+^CD11a^+^CD45RO^+^, consistent with highly activated and dysfunctional, transitory-like cytotoxic T lymphocytes (CTL)^44^. We also observed three CD45RO-negative metaclusters (M6, M7, M8). Metacluster M6 was CD127^+^TIM-3^−^GrzB^−^, but expressed PD-L1, CD25, FoxP3, TGF-β, IL-10, and CTLA-4 suggesting it had characteristics of a dysfunctional precursor-like cell. On the other hand, metacluster M7 was CD127^−^CD27^−^GrzB^−^CD11a^+^, consistent with terminally differentiated cells; M8 was also CD127^−^ and lacked expression of co-stimulatory molecules (OX40, 4-1BB, CD27) but was PD1^+^PD-L1^+^GrzB^+^TIM-3^+^TGF-β^+^, suggestive of terminally exhausted CTL. These studies support CEACAM1 expression on CD8^+^ T cells that have characteristics of precursor, transitory, and terminally differentiated CTL^45,46^.

## Discussion

Here, we used a highly specific antibody that is predicted to detect all glycoforms and splice variants of human CEACAM1 to provide a comprehensive assessment of human CEACAM1 expression on tumor-infiltrating immune cells through an analysis of melanoma. Further, through our inclusion of healthy donors and paired-peripheral blood and tumor samples according to treatment status we provide detailed insight into the tumor-specific states and association with treatment that characterize this expression. As such, we show that CEACAM1 is largely absent or at low levels on healthy circulating immune cells but increased on immune cells in the peripheral blood and tumors of patients with melanoma. Notably, virtually all circulating PD1^Hi^ NK cells, innate T cells, B cells, monocytic cells, dendritic cells, and CD4^+^ T cells in the peripheral blood of treatment-resistant disease were observed to co-express CEACAM1 and demonstrable as discrete populations implicating this trait as a tractable biomarker with potential therapeutic implications. Further, we show CEACAM1 is present on distinct types of cells that are unique to the TME and exhibit expression levels that are highest in treatment resistance and potentially disease progression. Together with our detailed analyses of the individual cell types by other approaches, our studies allow for the broad conclusion that CEACAM1 expression is associated with treatment-resistant disease and likely operates through cell-specific mechanisms; understanding these specific mechanisms will be a major focus in future studies as discussed further below.

In the case of B cells, we showed that CEACAM1 is observed on multiple stages of B cell differentiation within the tumors and circulation of melanoma patients. This includes naive-memory, memory, activated, and double-negative (DN) B cells. In contrast, we observed limited CEACAM1 expression on circulating healthy human B cells which is consistent with another report^47^. We have also identified that the tumors from patients with treatment-resistant disease possessed lower relative proportions of DN B cells, activated B cells, and a highly heterogeneous group of phenotypically naive cells in association with significantly increased CEACAM1 expression on the same cell subsets relative to that observed in treatment-naive melanoma patients; this suggests that the decreased cell numbers may inversely be related to CEACAM1 expression. Further, although B cell expression of CEACAM1 was observed in treatment-naive patients, the highest levels of expression occurred in treatment-resistant disease. In addition to naive- and memory-like B cells, increased CEACAM1 expression in treatment resistance was observed on activated and DN B cells which in the latter case are considered memory-like^48^. These may represent a distinct route of B cell differentiation to antibody-producing B cells in association with extrafollicular pathways^49^. DN B cell responses are notable as they are involved in vaccine responses^50^ and in promoting autoimmunity or preventing infectious diseases^49^. Our studies together suggest that CEACAM1 expression is predominantly associated with and may suppress memory-related B cell responses in the tumor microenvironment.

In humans, CEACAM1 has been suggested to be an inhibitory co-receptor based upon studies in the Daudi B cell line which show that B cell receptor (BCR) activation leads to CEACAM1 phosphorylation; this is known to be the first step in the recruitment of Src-homology phosphatases such as SHP1 and inhibition of signaling^13,47^. The latter would be predicted to include Syk and immunoreceptor tyrosine-based activation motif-bearing receptors such CD79a and CD79b which are potential targets of SHP1 and play an important role in BCR signaling^51^. *CEACAM1* and *CD79A* interestingly map as adjacent genes on chromosome 19 suggesting they may be co-regulated^52^. CEACAM1-expressing DN and memory-related B cells were also characterized by CXCR5 expression which is responsive to the potent B cell chemoattractant CXCL13 and critical for BCR-triggered B cell activation in secondary lymphoid structures; this suggests CEACAM1 may regulate BCR responses to this critical chemokine ligand^53^. Similarly, these CEACAM1-bearing B cell subsets expressed CXCR3 which may mark plasma cell precursors^54^. These studies together suggest CEACAM1 may restrain BCR responses to local antigenic cues at multiple stages of B cell differentiation but especially those associated with memory B cell responses and their transition to productive effector cells. As recent studies support an important role for B cells in determining responses to immunotherapy^55^, including those associated with the activity of ectopic lymphoid follicles^56,57^, our studies imply CEACAM1 function in these tumor-associated B cell responses.

There is very little information on CEACAM1 expression or function in human monocytic cells or DC. Transcriptional evidence supporting CEACAM1 expression has previously been shown for human DC^58^ and data in cultured primary human monocytes or monocyte-derived DC from peripheral blood have suggested CEACAM1 expression may promote their survival^59^ and inhibit their functionality^60^. We now show that CEACAM1 is largely restricted from being expressed on monocytic cells or DCs under homeostatic conditions, but neo-expressed on both types of cells in the setting of melanoma. There are several common themes that emerged from these studies with respect to monocytic and dendritic cells. The first is that in both cases CEACAM1 expression was increased in the tumor-associated cells of the treatment-naive and -resistant patients. However, the highest levels were observed in and correlated with treatment resistance. Secondly, we found that CEACAM1 expression marked types and states of monocytic cells and DCs that were uniquely associated with the tumors or shared with those in the circulation. Finally, we observed that whereas monocytic cells and DC in the tumors often co-expressed CEACAM1, PD1, and/or PD-L1, those within the circulation only co-expressed CEACAM1 and PD1; this was not observed in the healthy donors. This suggests that CEACAM1 is involved in the immunoregulatory functions of these cells^61,62^. Further, PD-L1 expression is associated with dysfunctional monocytic cells and dendritic cells in association with a poor prognosis^63–65^. PD-L1 functions in cis by restricting T cell access to CD80 or to trans-ligate PD1 and thereby contributes significantly to local immune suppression^66,67^. This raises the possibility that cis ligands of CEACAM1 also exist on myeloid cells such as TIM-3^8^ or alternatively homophilic or heterophilic CEACAM1 engagement with other cell types may inhibit their function. Our studies overall suggest that, like its expression on B cells, CEACAM1 may be involved in disease progression through its display on monocytic cells and DC in association with treatment resistance.

CEACAM1 is well-studied in human T cells relative to other immune cells and considered an activation-associated marker that inhibits T cell receptor (TCR) signaling^21,68,69^. This occurs through the ability of CEACAM1 to associate with the TCR/CD3 complex and recruit Src-homology phosphatases to its two cytoplasmic tail-associated immunoreceptor tyrosine-based inhibitory motifs for the dephosphorylation of CD3-ζ and ZAP70^68^. In the case of CD4^+^ T cells, previous studies have reported low CEACAM1 levels within the peripheral blood of healthy patients^70^ and expression in the placenta^71^ and diseased tissues. The latter include human autoimmune diseases, such as celiac disease^21^ and multiple sclerosis^72^, infections, such as human immunodeficiency virus^8^, and tumors, such as colorectal cancer^73^, glioblastoma^74^, melanoma^75^ and head and neck tumors^76–79^ often in association with markers indicative of dysfunctional T cells such as TIM-3; this is consistent with mouse studies showing CEACAM1 is involved in and necessary for the development of T cell tolerance^8^. Here, we significantly extend these studies by specifically showing increased CEACAM1 expression on antigen-experienced CD4^+^ effector-memory T cells (TEM) and TFH cells, which in the latter case has also been observed in mouse models^80^. We also show that elevated CEACAM1 expression on TFH cells is observed together with increased PD1 expression; as extrinsic ligation of PD1 on TFH by other PD-L1 bearing cells may lead to TFH dysfunction, elevated CEACAM1 expression on TFH cells may serve a similar purpose^81,82^. Further, we observed that CEACAM1 expression was increased on cells phenotypically consistent with CD4^+^ Th2 T cells. Activation of CEACAM1 in mouse models results in inhibition of Th1 responses in association with decreased T-bet expression^83^. This indirectly suggests CEACAM1 expression may foster Th2 responses in the tumor. We also reveal evidence for CEACAM1 expression on CD4^+^ T cells that are phenotypically consistent with FoxP3^+^ regulatory T cells whose presence correlates with the melanoma-associated TME using multiple approaches. Increased CEACAM1 expression on CD4^+^ Treg was observed in treatment-naive and -resistant disease with the highest levels in the latter context. Interestingly, *CEACAM1* expression could be identified in regulatory T cells from 16/20 cancer types associated with a pan-cancer single-cell landscape analysis of tumor-infiltrating T cells suggesting these findings will extend to other cancer types^84^. It is also noteworthy that CEACAM4, an activating CEACAM family member, may also mark regulatory T cells^85^ suggesting that CEACAM1 and CEACAM4 may have opposing functions within this cell type. Moreover, CEACAM1 and CEACAM4 have been recently identified as potential ligands for PD1 and PD-L2, respectively^6^ which deserves further study, particularly in the context of Treg cell function in the TME.

We also showed that CEACAM1 is expressed on multiple stages of CD8^+^ T cell differentiation and that this expression is notably present in treatment resistance. In the first case, we observed increased CEACAM1 expression on phenotypically, naive CD45RA^+^ cells which was surprising as CEACAM1 is considered an activation antigen^13,21^. This finding led us to discover that CEACAM1 is expressed on CD45RA^+^CD8^+^ T cells phenotypically consistent with TSCM cells, terminally differentiated CD8^+^ T cells that express naive T cell and memory markers (so-called TEMRA cells), and terminally exhausted CD8^+^ T cells^41,42,86,87^. Further, we found that the CEACAM1^+^ terminally exhausted cells described here were highly dysfunctional, as evidenced by expression of FoxP3 as well as inhibitory cytokines (TGF-β, IL-10) and cell surface proteins (PD1, PD-L1, TIM-3) together with granzyme-B expression. Although CEACAM1 was co-expressed with TIM-3 on these terminally exhausted CD8^+^ T cells as previously shown in mouse models^8^, we found that CEACAM1 expression on TSCM-like cells was not associated with TIM-3 expression. This is consistent with the segregation of TIM-3 expression away from precursor cell populations and on highly exhausted CD8^+^ T cells^88^. The expression of CEACAM1 on TSCM-like cells included a subset that are PD1^+^CXCR5^+^ which distinctively may represent the precursors of exhausted CD8^+^ T cells (TPEX)^86,89^. Notably, the CEACAM1 cytoplasmic tail has homology to T cell transcription factors (TCF3-4) and can interact with β-catenin^90,91^; this is relevant as WNT signaling promotes TSCM cell differentiation^92^. As TPEX can be the precursors of exhausted cells, it is of further interest that, in addition to CEACAM1 expression on terminally exhausted CD8^+^ T cells, we observed CEACAM1 expression on dysfunctional transitory-like CTL in the tumors which may be part of a precursor-derived continuum^93^.

Finally, we observed an expansion of NK cells in the peripheral blood of the treatment-naive and -resistant patients which in the case of the treatment-resistant patients co-expressed CEACAM1 and PD1. CEACAM1 is known to be expressed on activated NK cells where it serves an inhibitory function by abrogating natural-killer group 2, member D (NKG2D)-signaling in response to NKG2D ligands expressed by tumor cells^94,95^. Interestingly, like PD1^96^, CEACAM1 has been shown to provide inhibitory function to NK cells in the absence of MHC class I on a target cell^97^. Their expression together on peripheral blood NK cells in the context of treatment resistance suggests that these two inhibitory proteins may synergize with each other and represent an accessible biomarker for this condition as we also observed for circulating monocytic cells, dendritic cells, innate T cells, and CD4^+^ T cells. This suggests that CEACAM1 expression on several types of immune cell types should be considered for longitudinal monitoring during therapeutic interventions in the future.

In summary, we used a highly specific antibody coupled with an unbiased set of detection platforms enabled by mass cytometry to parse out CEACAM1 expression in fine detail on human immune cells in healthy donor controls and melanoma and in relation to other immune checkpoint markers. These studies show that, like T cells which largely express CEACAM1 after activation either through cognate or non-cognate mechanisms^13^, CEACAM1 is likely regulated in a similar manner on NK cells, innate T cells, B cells, monocytic cells, and dendritic cells. Furthermore, its expression in the tumor environment is often associated with distinct differentiation states that are not observed in the peripheral blood. This is especially the case for monocytic cells and DC. In addition, we reveal that CEACAM1 is expressed on human CD4^+^ T cells including TEM-like cells with phenotypic evidence of deviation to Th2 cells as well as TFH and Treg cells. Its expression on CD8^+^ T cells also marks those with stem cell-like precursor properties as well as exhausted and terminally differentiated cells suggesting that CEACAM1 may be functionally associated with this developmental lineage. We also observed that the highest levels of CEACAM1 expression are observed in treatment-resistant tumors and provide evidence that such expression on subsets of B cells, monocytic cells, dendritic cells, and T cells is associated with this condition implicating a role for CEACAM1 in regulating disease progression through its activities on these cell types. These studies suggest CEACAM1 may contribute to melanoma pathogenesis by affecting multiple immune subtypes. It will be important in future studies to define the functional correlates of these findings for each cell type and the potential role played by distinct CEACAM1 isoforms that are characterized by either a long or short cytoplasmic tail. Moreover, our studies may assist in the therapeutic monitoring of melanoma and lay the groundwork for the evaluation of CEACAM1 in other disorders.

### Supplementary information

Supplementary Information

Description of Additional Supplementary Files

Supplementary Data 1

Supplementary Data 2

Supplementary Data 3

Supplementary Data 4

Supplementary Data 5

Supplementary Data 6

Supplementary Data 7

Supplementary Data 8

reporting summary

## Data Availability

The high-dimensional mass cytometry data are available through Cytobank at premium.cytobank.org/cytobank/experiments#public and upon request. Source data for the figures are available In Supplementary Information as Supplementary Data 1–8 and the legends of these are contained within the Description of Additional Supplementary Data Files.

## References

[1] Topalian SL (2012). Safety, activity, and immune correlates of anti-PD-1 antibody in cancer. N. Engl. J. Med..

[2] Brahmer JR (2012). Safety and activity of anti-PD-L1 antibody in patients with advanced cancer. N. Engl. J. Med..

[3] Ribas A, Wolchok JD (2018). Cancer immunotherapy using checkpoint blockade. Science.

[4] Topalian SL, Drake CG, Pardoll DM (2015). Immune checkpoint blockade: a common denominator approach to cancer therapy. Cancer Cell.

[5] Okazaki T, Honjo T (2007). PD-1 and PD-1 ligands: from discovery to clinical application. Int. Immunol..

[6] Wojtowicz WM (2020). A human IgSF cell-surface interactome reveals a complex network of protein-protein interactions. Cell.

[7] Kim WM, Huang YH, Gandhi A, Blumberg RS (2019). CEACAM1 structure and function in immunity and its therapeutic implications. Semin. Immunol..

[8] Huang YH (2015). CEACAM1 regulates TIM-3-mediated tolerance and exhaustion. Nature.

[9] Huang YH (2016). Corrigendum: CEACAM1 regulates TIM-3-mediated tolerance and exhaustion. Nature.

[10] Lake CM (2021). TIM-3 drives temporal differences in restimulation-induced cell death sensitivity in effector CD8(+) T cells in conjunction with CEACAM1. Cell Death Dis..

[11] Zhang D (2020). Identification and characterization of M6903, an antagonistic anti-TIM-3 monoclonal antibody. Oncoimmunology.

[12] Gandhi AK (2018). High resolution X-ray and NMR structural study of human T-cell immunoglobulin and mucin domain containing protein-3. Sci. Rep..

[13] Gray-Owen SD, Blumberg RS (2006). CEACAM1: contact-dependent control of immunity. Nat. Rev. Immunol..

[14] Nichita L (2019). Comparative analysis of CEACAM1 expression in thin melanomas with and without regression. Oncol. Lett..

[15] Sivan S (2012). Serum CEACAM1 correlates with disease progression and survival in malignant melanoma patients. Clin. Dev. Immunol..

[16] Dankner M, Gray-Owen SD, Huang YH, Blumberg RS, Beauchemin N (2017). CEACAM1 as a multi-purpose target for cancer immunotherapy. Oncoimmunology.

[17] Gandhi AK (2021). Structural basis of the dynamic human CEACAM1 monomer-dimer equilibrium. Commun. Biol..

[18] Dudley ME (2010). CD8+ enriched “Young” tumor infiltrating lymphocytes can mediate regression of metastatic melanoma. Clin. Cancer Res..

[19] Schulz AR (2019). Stabilizing antibody cocktails for mass cytometry. Cytom. A.

[20] Watt SM (2001). Homophilic adhesion of human CEACAM1 involves N-terminal domain interactions: structural analysis of the binding site. Blood.

[21] Morales VM (1999). Regulation of human intestinal intraepithelial lymphocyte cytolytic function by biliary glycoprotein (CD66a). J. Immunol..

[22] Maecker HT, McCoy JP, Nussenblatt R (2012). Standardizing immunophenotyping for the Human Immunology Project. Nat. Rev. Immunol..

[23] Amir el AD (2013). viSNE enables visualization of high dimensional single-cell data and reveals phenotypic heterogeneity of leukemia. Nat. Biotechnol..

[24] Mamessier E (2011). Human breast cancer cells enhance self tolerance by promoting evasion from NK cell antitumor immunity. J. Clin. Invest..

[25] Van Gassen S (2015). FlowSOM: Using self-organizing maps for visualization and interpretation of cytometry data. Cytom. A.

[26] Bruggner RV, Bodenmiller B, Dill DL, Tibshirani RJ, Nolan GP (2014). Automated identification of stratifying signatures in cellular subpopulations. Proc. Natl Acad. Sci. USA.

[27] Weisel NM (2022). Surface phenotypes of naive and memory B cells in mouse and human tissues. Nat. Immunol..

[28] Petty, A. J. et al. Hedgehog-induced PD-L1 on tumor-associated macrophages is critical for suppression of tumor-infiltrating CD8+ T cell function. *JCI Insight*10.1172/jci.insight.146707 (2021). 10.1172/jci.insight.146707 PMC8026184 33749663

[29] Gordon SR (2017). PD-1 expression by tumour-associated macrophages inhibits phagocytosis and tumour immunity. Nature.

[30] Cai J (2019). The role of PD-1/PD-L1 axis and macrophage in the progression and treatment of cancer. J. Cancer Res. Clin. Oncol..

[31] Butler KL, Clancy-Thompson E, Mullins DW (2017). CXCR3+ monocytes/macrophages are required for establishment of pulmonary metastases. Sci. Rep..

[32] Maolake A (2017). Tumor-associated macrophages promote prostate cancer migration through activation of the CCL22-CCR4 axis. Oncotarget.

[33] Berlato C (2017). A CCR4 antagonist reverses the tumor-promoting microenvironment of renal cancer. J. Clin. Investig..

[34] Kadomoto, S. et al. Tumor-associated macrophages induce migration of renal cell carcinoma cells via activation of the CCL20-CCR6 axis. *Cancers*10.3390/cancers12010089 (2019). 10.3390/cancers12010089 PMC7017081 31905918

[35] Park SJ (2014). Negative role of inducible PD-1 on survival of activated dendritic cells. J. Leukoc. Biol..

[36] Karyampudi L (2016). PD-1 blunts the function of ovarian tumor-infiltrating dendritic cells by inactivating NF-κB. Cancer Res.

[37] Krempski J (2011). Tumor-infiltrating programmed death receptor-1+ dendritic cells mediate immune suppression in ovarian cancer. J. Immunol..

[38] Peng Q (2020). PD-L1 on dendritic cells attenuates T cell activation and regulates response to immune checkpoint blockade. Nat. Commun..

[39] Qiu X (2017). Reversed graph embedding resolves complex single-cell trajectories. Nat. Methods.

[40] Kamphorst AO (2017). Proliferation of PD-1+ CD8 T cells in peripheral blood after PD-1-targeted therapy in lung cancer patients. Proc. Natl Acad. Sci. USA.

[41] Yang D-H (2019). Genomic profiles and subset characterization of CD8+terminally differentiated effector memory (TEMRA) cells from cancer patients. Blood.

[42] Gattinoni L, Speiser DE, Lichterfeld M, Bonini C (2017). T memory stem cells in health and disease. Nat. Med..

[43] Katsuyama E (2020). The CD38/NAD/SIRTUIN1/EZH2 axis mitigates cytotoxic CD8 T cell function and identifies patients with SLE prone to infections. Cell Rep..

[44] Hudson WH (2019). Proliferating transitory T cells with an effector-like transcriptional signature emerge from PD-1+ stem-like CD8+ T cells during chronic infection. Immunity.

[45] Im SJ (2016). Defining CD8+ T cells that provide the proliferative burst after PD-1 therapy. Nature.

[46] Lugli E, Galletti G, Boi SK, Youngblood BA (2020). Stem, effector, and hybrid states of memory CD8(+) T cells. Trends Immunol..

[47] Lobo EO, Zhang Z, Shively JE (2009). Pivotal advance: CEACAM1 is a negative coreceptor for the B cell receptor and promotes CD19-mediated adhesion of B cells in a PI3K-dependent manner. J. Leukoc. Biol..

[48] Golinski ML (2020). CD11c(+) B cells are mainly memory cells, precursors of antibody secreting cells in healthy donors. Front. Immunol..

[49] Sanz, I. et al. Challenges and opportunities for consistent classification of human B cell and plasma cell populations. *Front. Immunol.*10.3389/fimmu.2019.02458 (2019). 10.3389/fimmu.2019.02458 PMC6813733 31681331

[50] Ruschil, C. et al. Specific induction of double negative B cells during protective and pathogenic immune responses. *Front. Immunol.*10.3389/fimmu.2020.606338 (2020). 10.3389/fimmu.2020.606338 PMC7775384 33391273

[51] Cyster JG, Allen CDC (2019). B cell responses: cell interaction dynamics and decisions. Cell.

[52] Kammerer R, Zimmermann W (2010). Coevolution of activating and inhibitory receptors within mammalian carcinoembryonic antigen families. BMC Biol..

[53] Sáez de Guinoa J, Barrio L, Mellado M, Carrasco YR (2011). CXCL13/CXCR5 signaling enhances BCR-triggered B-cell activation by shaping cell dynamics. Blood.

[54] Muehlinghaus G (2005). Regulation of CXCR3 and CXCR4 expression during terminal differentiation of memory B cells into plasma cells. Blood.

[55] Fässler M (2019). Antibodies as biomarker candidates for response and survival to checkpoint inhibitors in melanoma patients. J. Immunother. Cancer.

[56] Cabrita R (2020). Tertiary lymphoid structures improve immunotherapy and survival in melanoma. Nature.

[57] Helmink BA (2020). B cells and tertiary lymphoid structures promote immunotherapy response. Nature.

[58] Kammerer R, Stober D, Singer BB, Obrink B, Reimann J (2001). Carcinoembryonic antigen-related cell adhesion molecule 1 on murine dendritic cells is a potent regulator of T cell stimulation. J. Immunol..

[59] Yu Q (2006). CEACAM1 (CD66a) promotes human monocyte survival via a phosphatidylinositol 3-kinase- and AKT-dependent pathway. J. Biol. Chem..

[60] Yu Q (2013). Association of Neisseria gonorrhoeae Opa(CEA) with dendritic cells suppresses their ability to elicit an HIV-1-specific T cell memory response. PLoS ONE.

[61] Haderk, F. et al. Tumor-derived exosomes modulate PD-L1 expression in monocytes. *Sci. Immunol.*10.1126/sciimmunol.aah5509 (2017). 10.1126/sciimmunol.aah5509 28754746

[62] Zhang X (2017). PD-L1 induced by IFN-γ from tumor-associated macrophages via the JAK/STAT3 and PI3K/AKT signaling pathways promoted progression of lung cancer. Int J. Clin. Oncol..

[63] Yasuoka H (2020). Increased both PD–L1 and PD–L2 expressions on monocytes of patients with hepatocellular carcinoma was associated with a poor prognosis. Sci. Rep..

[64] Bianchini M (2019). PD-L1 expression on nonclassical monocytes reveals their origin and immunoregulatory function. Sci. Immunol..

[65] Ando K (2021). A high number of PD-L1+ CD14+ monocytes in peripheral blood is correlated with shorter survival in patients receiving immune checkpoint inhibitors. Cancer Immunol., Immunother..

[66] Zhao Y (2019). PD-L1:CD80 Cis-heterodimer triggers the co-stimulatory receptor CD28 while repressing the inhibitory PD-1 and CTLA-4 pathways. Immunity.

[67] Sugiura D (2019). Restriction of PD-1 function by cis-PD-L1/CD80 interactions is required for optimal T cell responses. Science.

[68] Chen Z, Chen L, Qiao SW, Nagaishi T, Blumberg RS (2008). Carcinoembryonic antigen-related cell adhesion molecule 1 inhibits proximal TCR signaling by targeting ZAP-70. J. Immunol..

[69] Nagaishi T (2006). SHP1 phosphatase-dependent T cell inhibition by CEACAM1 adhesion molecule isoforms. Immunity.

[70] Moller MJ, Kammerer R, Grunert F, von Kleist S (1996). Biliary glycoprotein (BGP) expression on T cells and on a natural-killer-cell sub-population. Int J. Cancer.

[71] Markel G (2002). Pivotal role of CEACAM1 protein in the inhibition of activated decidual lymphocyte functions. J. Clin. Invest..

[72] Piancone F (2019). A deficit of CEACAM-1-expressing T lymphocytes supports inflammation in primary progressive multiple sclerosis. J. Immunol..

[73] Zhang Y (2017). Co-expression of TIM-3 and CEACAM1 promotes T cell exhaustion in colorectal cancer patients. Int Immunopharmacol..

[74] Li J (2018). Abnormal expression of circulating and tumor-infiltrating carcinoembryonic antigen-related cell adhesion molecule 1 in patients with glioma. Oncol. Lett..

[75] Markel G (2010). Systemic dysregulation of CEACAM1 in melanoma patients. Cancer Immunol. Immunother..

[76] Yang F, Zeng Z, Li J, Ren X, Wei F (2021). TIM-3 and CEACAM1 are prognostic factors in head and neck squamous cell carcinoma. Front. Mol. Biosci..

[77] Weng CY, Hu XY, Wang YJ (2020). Integrated analysis of gene expression, alteration and clinical significance of carcinoembryonic antigen-related cell adhesion molecule 1 in cancer. 3 Biotech.

[78] Tam K (2018). Assessing the impact of targeting CEACAM1 in head and neck squamous cell carcinoma. Otolaryngol. Head. Neck Surg..

[79] Mattox, A. K. et al. Myeloid cells are enriched in tonsillar crypts, providing insight into the host tropism of human papillomavirus. *Am. J. Pathol.* **191**, 1774–1786 (2021). 10.1016/j.ajpath.2021.06.012 PMC8491092 34303699

[80] Chen L (2012). The short isoform of the CEACAM1 receptor in intestinal T cells regulates mucosal immunity and homeostasis via Tfh cell induction. Immunity.

[81] Shi J (2018). PD-1 controls follicular T helper cell positioning and function. Immunity.

[82] Zhou ZQ (2016). Follicular helper T cell exhaustion induced by PD-L1 expression in hepatocellular carcinoma results in impaired cytokine expression and B cell help, and is associated with advanced tumor stages. Am. J. Transl. Res..

[83] Iijima H (2004). Specific regulation of T helper cell 1-mediated murine colitis by CEACAM1. J. Exp. Med..

[84] Zheng L (2021). Pan-cancer single-cell landscape of tumor-infiltrating T cells. Science.

[85] Bhairavabhotla R (2016). Transcriptome profiling of human FoxP3+ regulatory T cells. Hum. Immunol..

[86] Kallies A, Zehn D, Utzschneider DT (2020). Precursor exhausted T cells: key to successful immunotherapy?. Nat. Rev. Immunol..

[87] McLane LM, Abdel-Hakeem MS, Wherry EJ (2019). CD8 T cell exhaustion during chronic viral infection and cancer. Annu Rev. Immunol..

[88] Miller BC (2019). Subsets of exhausted CD8(+) T cells differentially mediate tumor control and respond to checkpoint blockade. Nat. Immunol..

[89] Brummelman J (2018). High-dimensional single cell analysis identifies stem-like cytotoxic CD8(+) T cells infiltrating human tumors. J. Exp. Med..

[90] Li Y, Shively JE (2013). CEACAM1 regulates Fas-mediated apoptosis in Jurkat T-cells via its interaction with β-catenin. Exp. Cell Res..

[91] Jin L (2008). Direct interaction of tumor suppressor CEACAM1 with beta catenin: identification of key residues in the long cytoplasmic domain. Exp. Biol. Med..

[92] Gattinoni L (2009). Wnt signaling arrests effector T cell differentiation and generates CD8+ memory stem cells. Nat. Med..

[93] Galletti G (2020). Two subsets of stem-like CD8(+) memory T cell progenitors with distinct fate commitments in humans. Nat. Immunol..

[94] Hosomi S (2013). CEACAM1 on activated NK cells inhibits NKG2D-mediated cytolytic function and signaling. Eur. J. Immunol..

[95] Markel G (2004). The critical role of residues 43R and 44Q of carcinoembryonic antigen cell adhesion molecules-1 in the protection from killing by human NK cells. J. Immunol..

[96] Quatrini L (2020). The immune checkpoint PD-1 in natural killer cells: expression, function and targeting in tumour immunotherapy. Cancers.

[97] Markel G (2004). The mechanisms controlling NK cell autoreactivity in TAP2-deficient patients. Blood.

