# Supplementary material for: High-dimensional mapping of human CEACAM1 expression on immune cells and association with melanoma drug resistance
Source: Commun Med (Lond). 2024 Jul 2;4:128. doi: 10.1038/s43856-024-00525-8 (PMC11219841; doi:10.1038/s43856-024-00525-8)
Supplement: Supplementary file 1 — Supplementary Information [file 43856_2024_525_MOESM1_ESM.pdf]

Supplementary Information:

High-dimensional mapping of human CEACAM1 expression on immune cells and association with melanoma drug resistance

Yu-Hwa Huang[1,9,10], Charles H. Yoon[2,10], Amit Gandhi[1], Thomas Hanley[1], Carlos Castrillon[3], Yasuyuki Kondo[1,12], Xi Lin[1], Walter Kim[1], Chao Yang[1], Amine Driouchi[4], Michael Carroll[3], Scott D. Gray-Owen[5], Duane R. Wesemann[1,6], Charles G. Drake[7,13], Monica M. Bertagnolli[2,11,14], Nicole Beauchemin[8,11], Richard S. Blumberg[1,9,11]

[1]Department of Medicine, Brigham and Women's Hospital, Harvard Medical School, Boston, MA, USA
[2]Department of Surgery, Brigham and Women's Hospital, Harvard Medical School, Boston, MA, USA
[3]Program in Cellular and Molecular Medicine, Children's Hospital Medical Center, Harvard Medical School, Boston, MA, USA
[4]Institute of Biomedical Engineering, University of Toronto, Toronto, Canada
[5]Department of Molecular Genetics, University of Toronto, Toronto, Canada
[6]Division of Allergy and Immunology, Division of Genetics, Brigham and Women's Hospital and Ragon Institute of MGH, MIT and Harvard, Boston, MA, USA
[7]Herbert Irving Comprehensive Cancer Center, Columbia University School of Medicine, New York, NY, USA
[8]Rosalind and Morris Goodman Cancer Institute, McGill University, Montreal, Canada

[9]To whom correspondence should be addressed: yhuang17@bwh.harvard.edu and rblumberg@bwh.harvard.edu

[10]These authors contributed equally
[11]These authors jointly supervised this work
Current address: [12]Department of Internal Medicine, Graduate School of Medicine, Kobe, University, Kobe, Japan
[13]Current Address: Janssen R&D, Springhouse, PA, USA
[14]Current Address: National Institutes of Health, Bethesda, MD, USA

Supplementary Figure 1

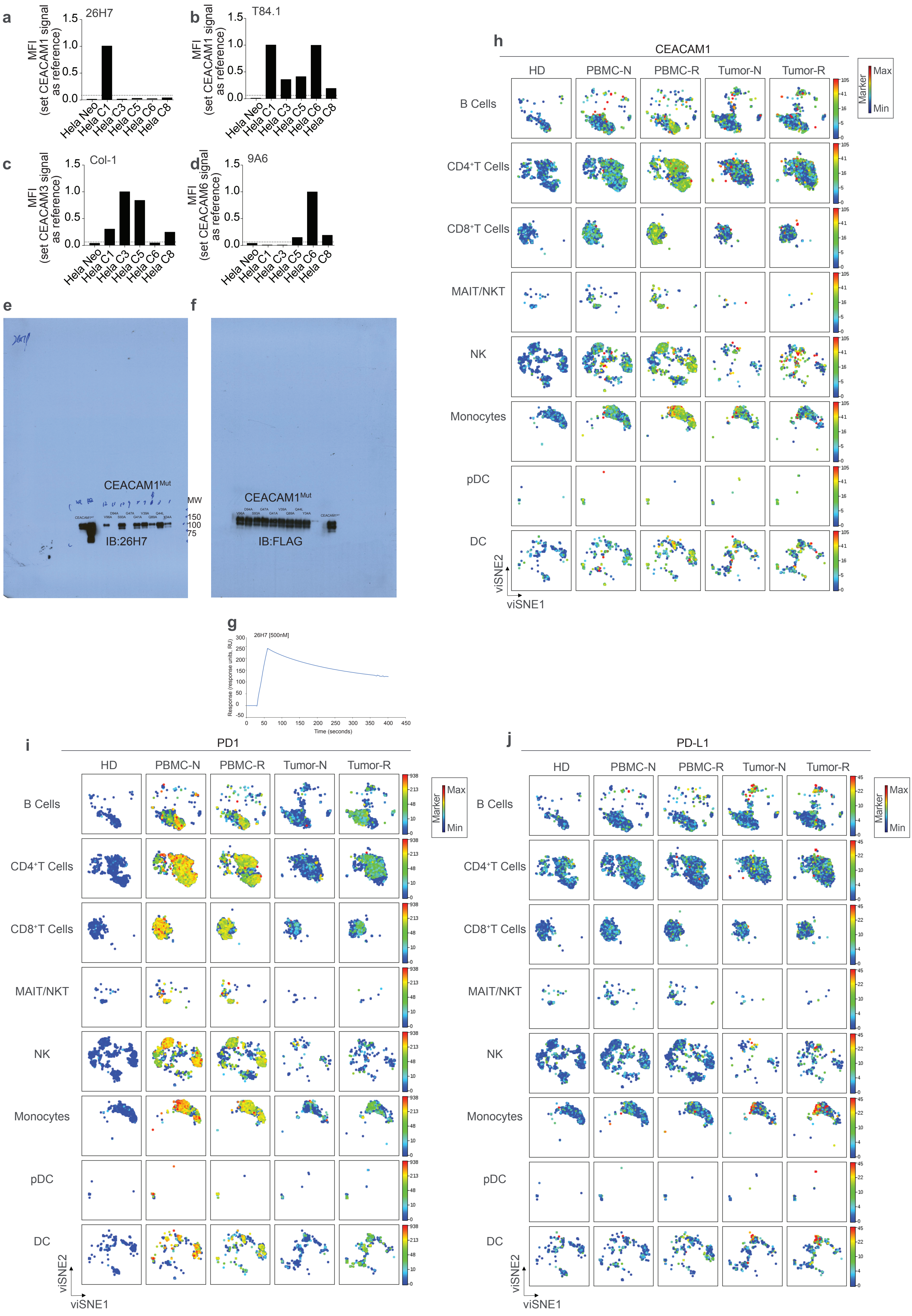

Supplementary Figure 1. Characterization of an anti-CEACAM1 antibody for mass cytometry

a-d. Flow cytometry staining of Hela cell line transfected with vector control (Hela Neo), hCEACAM1 (Hela C1), hCEACAM3 (Hela C3), hCEACAM5 (Hela C5), hCEACAM6 (Hela C6) or hCEACAM8 (Hela C8) stained with the 26H7 monoclonal antibody or mouse IgG1 isotype control antibody. The average background staining by the isotype control was set as 0 (dotted line) for each antibody and the levels of antibody staining of the transfected or vector control cells shown as histograms. In the case of the 26H7 (**a**) and T84.1 (**b**) antibodies, the level of Hela-hCEACAM1 transfected cells stained by the monoclonal antibody is shown as a relative level of 1. In the case of the Col-1 antibody (**c**), the level of Hela-hCEACAM3 transfected cells stained by the monoclonal antibody is shown as a relative level of 1. In the case of the 26H7 and T84.1 antibodies, the level of HelahCEACAM1 transfected cells stained by the monoclonal antibody is shown as a relative level of 1. In the case of the 9A6 antibody (**d**), the level of Hela-hCEACAM6 transfected cells stained by the monoclonal antibody is shown as a relative level of 1. These results are an example of 3 independent experiments;
e-f. Full uncropped immunoblots of hCEACAM1 wild-type (WT) and mutants of amino acids within the IgV domain face as indicated (Y34A, Q44L, Q89A, V39A, G41A, G47A, S93A, D94A, V96A) expressed as FLAG-tagged proteins in transfected HEK-293T cells and immunoblotted (IB) with the 26H7 mAb (**e**) or a FLAG-tag specific antibody (**f**). Molecular weights (MW) are indicated;
g. Surface plasmon resonance (SPR) binding of the 26H7 antibody with non-glycosylated CEACAM1 IgV-domain protein based on a previously published protocol[8,9], where the hCEACAM1 IgV-domain protein was captured using cys-biotin conjugation and attached over the neutravidin surface with 26H7 monoclonal antibody (500 nM) in the eluate; **h-j.** viSNE visualization of the cell types in PBMC of healthy donors (HD, n=5), treatmentnaive (PBMC-N) (n=7) and treatment-resistant (PBMC-R) melanoma patients (n=3) and dissociated tumor cells from treatment-naive (Tumor-N, n=9) and treatment-resistant (Tumor-R, n=10) melanoma patients overlaid with scaled median expression of levels of CEACAM1 (**h**; median value of ^{159}Tb), PD1 (**i**; median value of ^{169}Tm) and PD-L1 (**j**; median value of ^{175}Lu). The color-coded scale bars for each cell type are shown on the right (blue, minimum; red, maximum). MAIT/NKT, mucosal associated invariant T cells/natural killer T cells; NK, natural killer cells; pDC, plasmacytoid dendritic cells.

Supplementary Figure 2

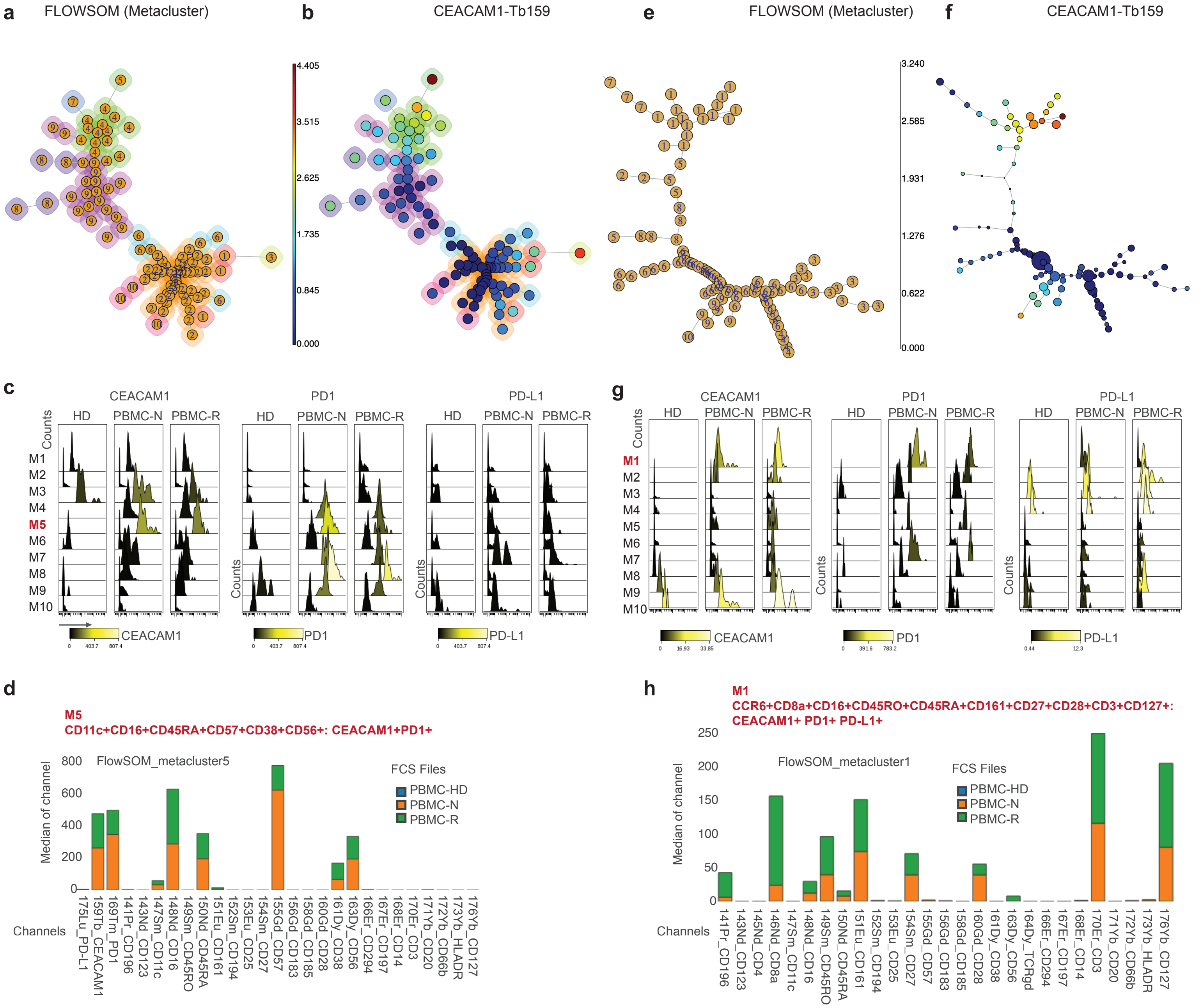

Supplementary Figure 2. Characterization of CEACAM1^{+}PD1^{+} NK and innate T cells by FlowSOM analysis

a. Map of the FlowSOM metacluster level analysis of the exported, concatenated NK cells from the peripheral blood mononuclear cells from healthy donors and treatment-naive and -resistant patients shown as a minimum spanning tree (MST) with the location of the metaclusters indicated and color-coded;
b. Level of CEACAM1 expression as shown in the FlowSOM analysis (**a**);
c. Histogram plots showing the levels of CEACAM1, PD1 and PD-L1 expression in metaclusters (M) from FlowSOM analysis as defined in (**a**). Note that FlowSOM M5-associated NK cells express CEACAM1 and PD1 and FlowSOM M3-associated NK cells express CEACAM1 but not PD1;
d. Bar graph depicting the levels of markers within the M5-associated NK cells;
e. Map of the FlowSOM metacluster level analysis of the exported, concatenated innate T cells from the peripheral blood mononuclear cells of healthy donors and treatment-naive and -resistant patients shown as an MST with the location of the metaclusters indicated and color-coded;
f. Level of CEACAM1 expression as shown in the FlowSOM analysis (**e**);
g. Histogram plots showing the levels of CEACAM1, PD1 and PD-L1 expression in metaclusters (M) from the FlowSOM analysis defined in (**e**). Note that FlowSOM M1-associated innate T cells express CEACAM1 and PD1;
h. Bar graph depicting the levels of markers on the M1-associated innate T cells.

Supplementary Figure 3

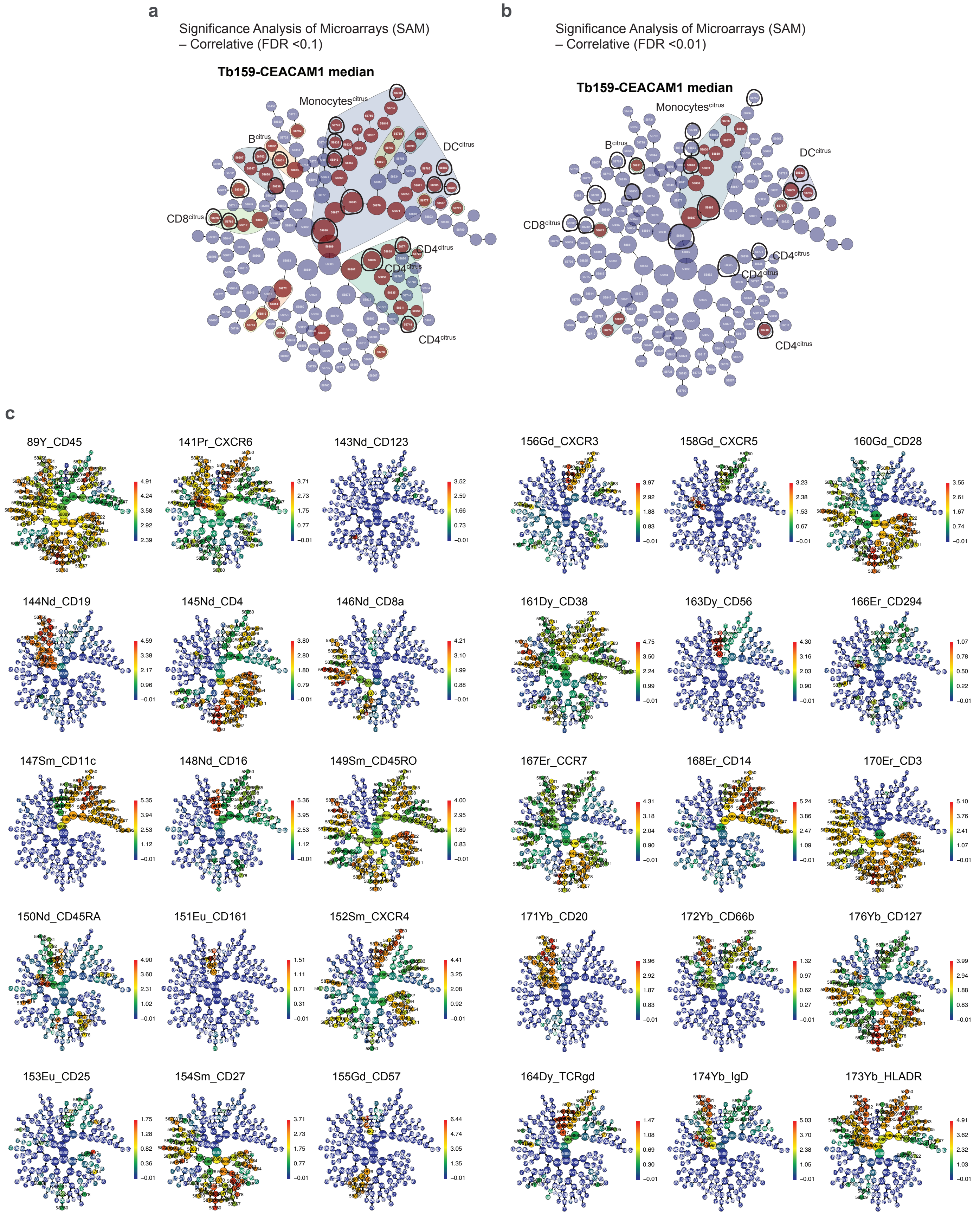

Supplementary Figure 3. Citrus models of metacluster associations with clinical phenotypes

a-b. Significance analysis of microarray (SAM) modeling at false discovery rates (FDR) of <0.1 (**a**), and <0.01 (**b**) is shown for defining the association between CEACAM1 expression and the global populations contained within the 5 clinical subtypes including PBMC from healthy donors (n=5), treatment-naive (n=7) and treatment-resistant (n=3) samples and the tumor cells dissociated from metastatic lesions from treatment-naive (n=9) and treatment-resistant (n=10) melanoma patients. These analyses concurred with each other and identified regionalized clusters within associated limbs of the radial hierarchical tree consistent with B cells, monocytes, dendritic cells, $CD4^+$ and $CD8^+$ T cells based upon the marker expression in the radial hierarchical tree as in **(c)**. Nodes examined for disease association are encircled in black;
c. Color-coded expression of each marker in the nodes associated with the radial hierarchical tree derived from Significance analysis of microarray (SAM) modeling. The levels of expression are indicated by the associated scale bar for each marker (blue, minimum; red, maximum).

Supplementary Figure 4

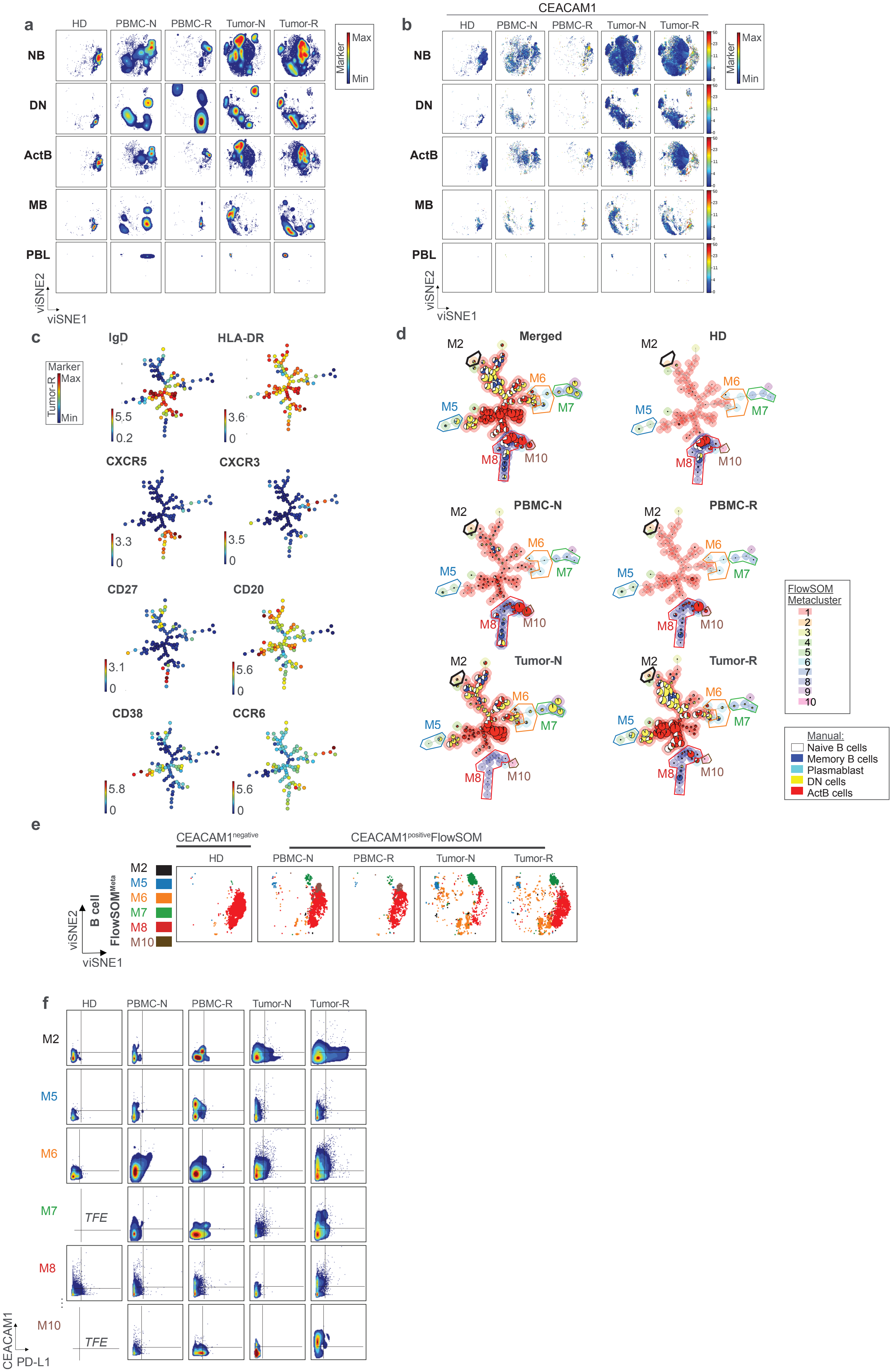

Supplementary Figure 4. Characterization of B cells

a. viSNE localization of manually-gated subsets of B cells within the 5 clinical types of samples. NB, naive B cell. DN, double-negative B cell. ActB, activated B cell. MB, memory B cell. PBL, plasmablast. HD, healthy donor. PBMC-N, naive peripheral blood mononuclear cells. PBMC-R, resistant PBMC. Tumor-N, naive tumor. Tumor-R, resistant tumor. The cellular intensity is proportional to cell number and shown as minimum (blue) and maximum (red);

b. The location in viSNE space of the scaled median expression of CEACAM1 (median value of ^{159}Tb) among the manually-gated clusters and clinical subsets as in (**a**). The color-coded scale bar showing minimum (blue) and maximum (red) is shown on right;

c. Channel-colored median expression of each marker within the minimum spanning tree (MST) associated with treatment-resistant tumor samples (Tumor-R) used in the annotation of each CEACAM1-expressing metacluster. The expression levels are color-coded as indicated by the scale bar (blue, minimum; red, maximum);

d. Overlay of manually-gated cell types as indicated in the colored legend as naive B cells, memory B cells, plasmablasts, double-negative (DN) B cells and Activated (Act) B cells on the FlowSOM defined metaclusters shown within the MST. The color-coded halo defines each metacluster within the MST and the contribution of the manually-gated cell types to each MST-associated node is shown in the individual pie-charts. The clinical samples are as in (**a**) and the location of the CEACAM1-expressing metaclusters (M) are outlined;

e. Visualization of the metaclusters as indicated in the color-coded legend in the viSNE space of the manually-gated B cells within the clinical subtypes as in (**a**);

f. Bivariant, dual marker density plots displaying CEACAM1 and PD-L1 expression in metaclusters (M) associated with the clinical subtypes as in (**a**). TFE, Too Few Events.

Supplementary Figure 5

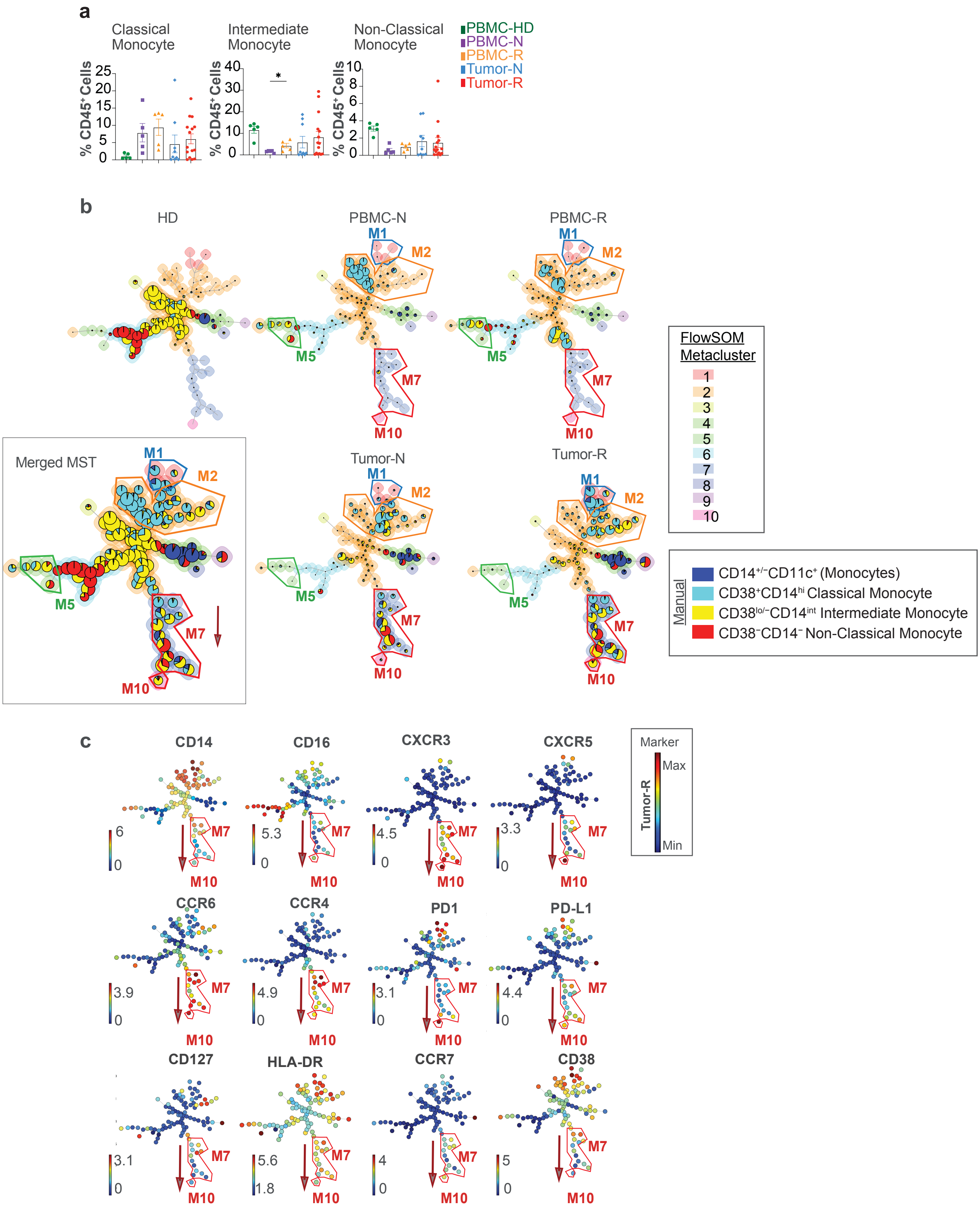

Supplementary Figure 5. Characterization of monocytic cells

a. Quantitation of manually-gated classical, transitional/intermediate and non-classical monocytic cells as a relative proportion of the total $CD45^+$ cells contained within each clinical type of sample analyzed including PBMC of healthy donors (HD, n=5), treatment-naive (PBMC-N, n=7) and treatment-resistant (PBMC-R, n=3) melanoma patients and dissociated tumor cells from treatment-naive (Tumor-N, n=9) and treatment-resistant (Tumor-R, n=10) melanoma patients. *, $p < 0.05$ significance by Kruskal-Wallis test, followed by the Dunn's multiple comparison test, error bar on graphs were plotted with standard error of the mean acquisition;
b. Overlay of manually-gated cell types defined as classical ($CD38^+CD14^{hi}$), transitional/intermediate ($CD38^{lo/-}CD14^{int}$), non-classical ($CD38^-CD14^-$) and unclassified ($CD14^{+/-}CD11c^+$) monocytic cells and metaclusters defined by FlowSOM and visualized as a minimum spanning tree (MST). The color-coded halo defines each metacluster within the MST and the contribution of the manually-gated cell types to each MST-associated node is shown in the individual pie-charts of the 5 clinical sample types as in (**a**). The CEACAM1-expressing metaclusters (M) are outlined and the red arrow shows a proposed direction of differentiation from classical to non-classical monocytic cells. A view of this analysis of the merged samples is shown;
c. Channel-colored scaled median expression of each marker within the MST associated with treatment-resistant tumor samples (Tumor-R) used in the annotation of each CEACAM1-expressing metacluster. The expression levels are color-coded as indicated by the scale bar (blue, minimum; red, maximum) and the arrow indicates a proposed differentiation pathway as suggested by the monocytic cell types in association with each node as in (**b**).

Supplementary Figure 6

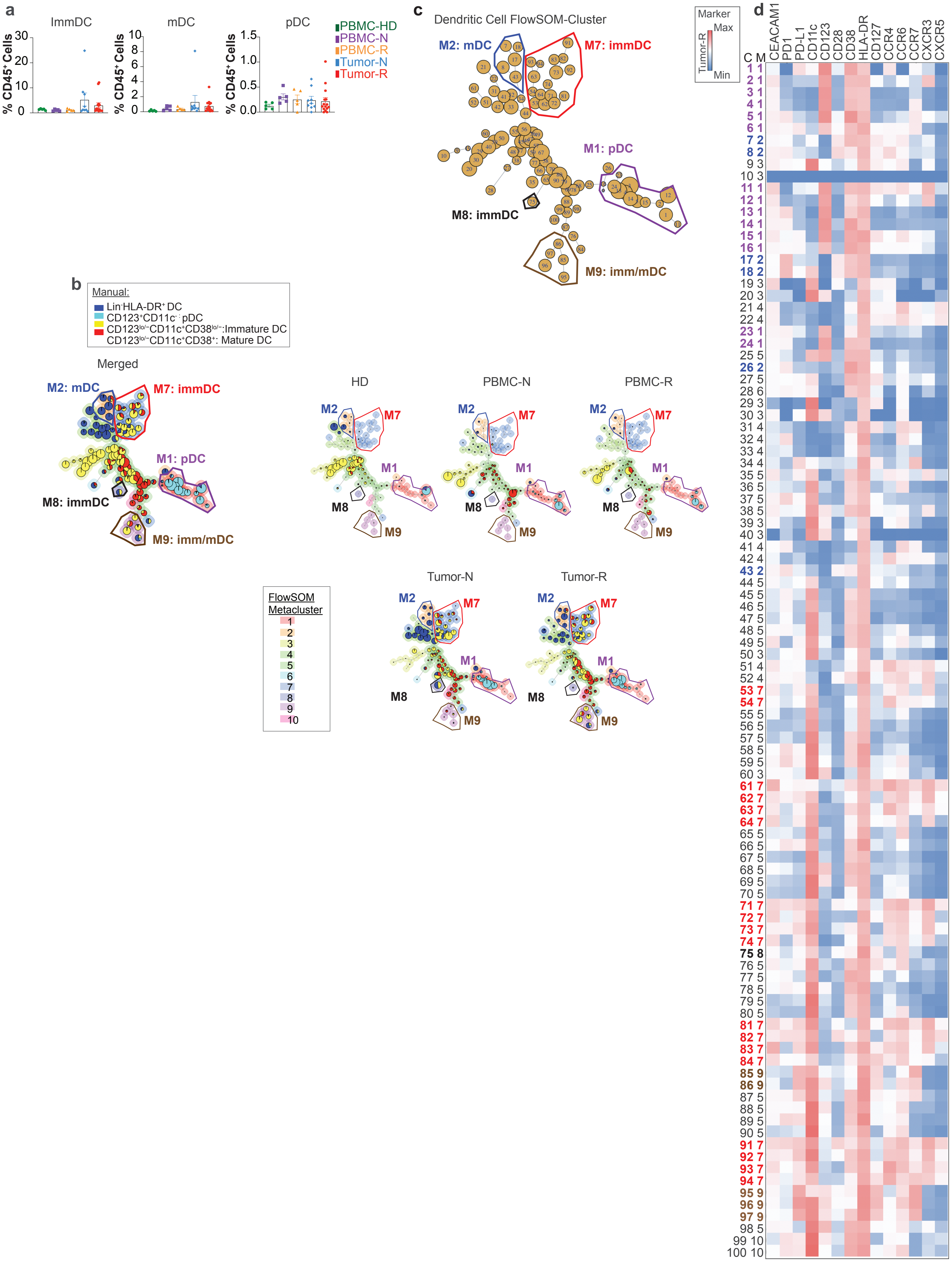

Supplementary Figure 6. Characterization of dendritic cells

a. Quantitation of manually-gated immature (HLA-DR^{+}CD123^{-}CD11c^{+}CD38^{-}), mature (HLA-DR^{+}CD123^{-}CD11c^{+}CD38^{+}) and plasmacytoid (HLA-DR^{+}CD123^{+}CD11c^{+}) dendritic cells (DC) as a relative proportion of the total CD45^{+} cells contained within each clinical type of sample analyzed including peripheral blood mononuclear cells (PBMC) of healthy donors (HD, n=5), treatment-naive (PBMC-N, n=7) and treatment-resistant (PBMC-R, n=3) melanoma patients and dissociated tumor cells from treatment-naive (Tumor-N, n=9) and treatment-resistant (Tumor-R, n=10) melanoma patients, error bar on graphs were plotted with standard error of the mean acquisition;
b. Overlay of manually-gated cell types defined as unassigned dendritic cells (DC) (Lin^{-} HLA-DR^{+}), plasmacytoid (p) DC (CD123^{+}CD11c^{-}), immature DC (CD123$^{lo/-}$CD11c^{+} CD38$^{lo/-}$) and mature DC (CD123$^{lo/-}$CD38^{+}) and metaclusters defined by FlowSOM and visualized as a minimum spanning tree (MST). The color-coded halo defines each metacluster within the MST and the contribution of the manually-gated cell types to each MST-associated node is shown in the individual pie-charts of the 5 clinical sample types as in (**a**). A view of this analysis of the merged samples is shown;
c. Map of the cluster level analysis of dendritic cells shown as an MST with the location of the CEACAM1-expressing metaclusters (M) indicated and color-coded;
d. Heatmap showing scaled expression of markers in treatment-resistant samples (Tumor-R) as indicated on top in identified cluster (C, 1-100) within each identified metacluster (M, 1-10) of monocytic cells as in (**c**). The location of relevant clusters is indicated and annotated according to the color code as in (**c**). The expression levels are color-coded as indicated by the scale bar (blue, minimum; red, maximum). C, cluster. M, metacluster.

Supplementary Figure 7

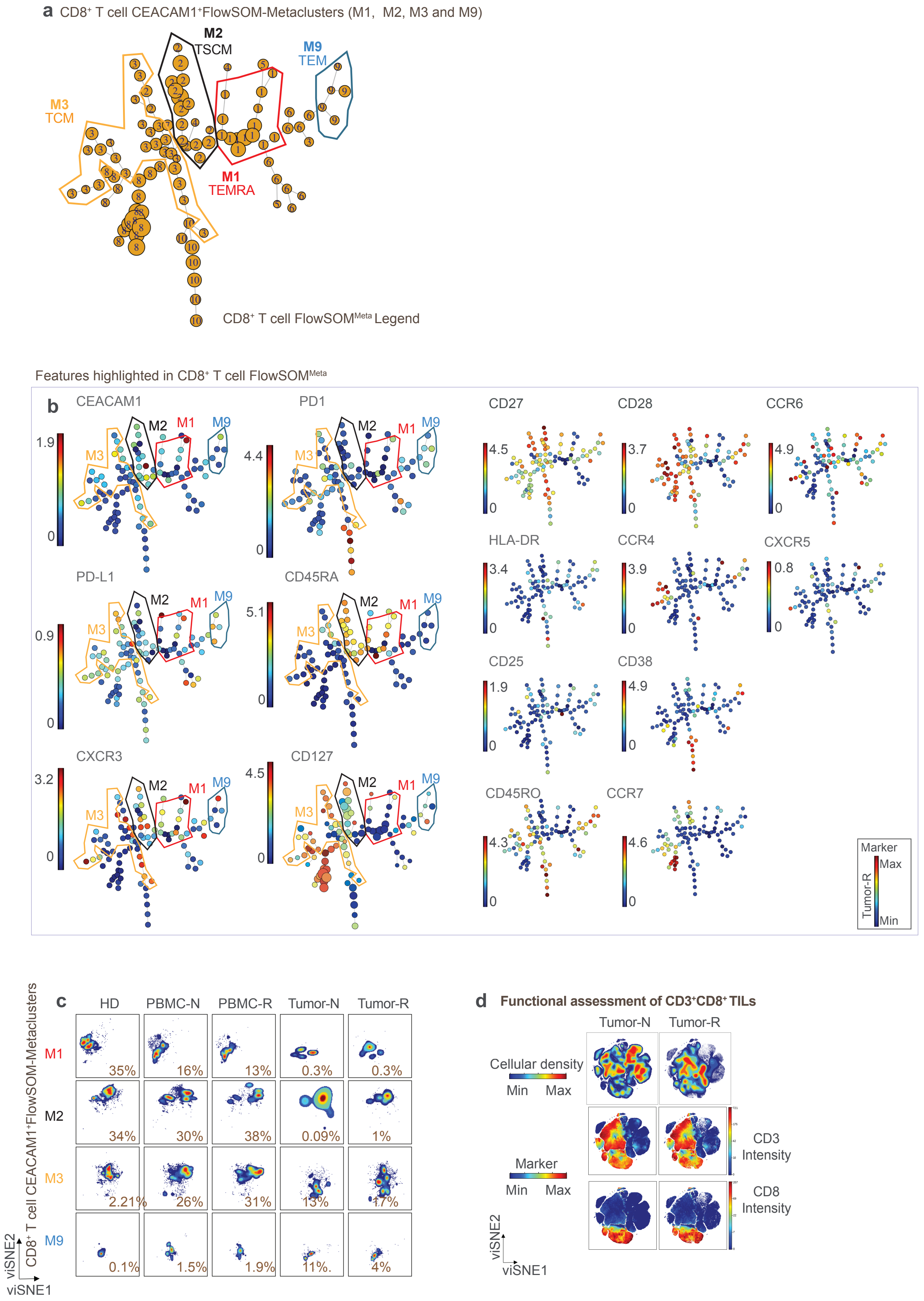

Supplementary Figure 7. Characterization of CD8^{+} T cells

a. Minimum spanning tree (MST) of exported and concatenated CD8^{+} T cells from the 5 patient types defined by a phenotypic panel of markers. The CEACAM1-expressing metaclusters (M1, M2, M3, M9) are indicated;
b. Channel-colored marker expression within the nodes associated with minimum spanning tree (MST) as in (**a**) derived from the FlowSOM analysis of the CD8^{+} T cells associated with treatment-resistant (R) tumor samples. Colored scale bar is shown for each marker (blue, minimum; red, maximum);
c. Visualization of CEACAM1-expressing metaclusters in viSNE space overlaid upon the exported CD8^{+} T cells in each type of patient sample. PBMC, peripheral blood mononuclear cells; HD, healthy donor; N, treatment-naive; R, treatment-resistant. Percentages of each metacluster relative to total CD8 T cells are indicated;
d. viSNE map of tumor-dissociated cells from treatment-naive (Tumor-N, n=9) and -resistant (Tumor-R, n=5) samples after staining with functional panel of markers. Global population is shown on top. Location of CD3- (middle) and CD4-expressing cells (bottom) are indicated. Scale bar indicating the quantity based upon cellular density and level of marker expression are indicated by the scale bars (blue, minimum; red, maximum). TILs, tumor infiltrating lymphocytes.

Patient sample	Prior treatment	Sample site	Primary disease	Stage	Phenotype	Function	Tumor	PBMC	Confocal
CY035.1	Vemurafenib x 12<br>wk, PD-L1 inhibitor x5	Small bowel	Scalp (T2a)	Stage IV	v		v	v	
CY109	Pembrolizumab<br>(R-CHOP for CLL)	L arm soft tissue metastasis	L forearm primary	Stage IIIc	v		v		
CY121.1	Pembrolizumab<br>WDVAX	R neck lymph node metastasis	R lower back primary, T2a	Stage IV	v		v		
CY138.1*	Nivolumab x2<br>TVEC injections<br>Pembrolizumab	L flank ulcerated, bleeding met<br>(persistent disease despite good<br>response at other adjacent sites)	L back/flank primary, T2a+<br>(excised 2013.03)	Stage IIIc	v		v		
CY139	Ipilimumab x4	L axillary LN metastasis	L shoulder primary, T4b	Stage IV	v		v		
CY141	Nivolumab	L axillary LN metastasis	Unknown primary	Stage IV	v		v		
CY143*	Pembrolizumab	R axillary soft tissue metastasis	Unknown primary	Stage IV	v		v		
CY145*	none	L shoulder satellite/in-transit metastasis	Unknown primary	Stage III vs. IV	v		v		
CY146*	Pembrolizumab<br>Ipilimumab + Nivolumab<br>Dabrafenib + Trametinib	R breast soft tissue metastasis	Back primary, T2b	Stage IV	v		v		
CY147P*, **	none	scalp primary<br>(sample is from primary lesion)	scalp primary, T4b	Stage IV	v		v		
CY148*	Nivolumab x11	L axillary LN	L back primary, T2a	Stage IIIc (T2a<br>N2c/N3c)	v		v		
CY149*, ***	none	R axillary LN metastasis	R forearm primary, T1a (1987)	Stage III	v		v		
CY149A*, ***					v		v		
CY150*	none	L flank soft tissue mass	L temple primary, T1a	Stage IV	v		v		
CY152*	Ipilimumab	L femoral LN metastasis	L thigh primary, T2b	Stage IIIb	v		v		
CY153*	none	R femoral LN metastasis	Unknown primary	Stage IIIc vs. IV	v		v		
CY154*	Nivolumab	R femoral LN metastasis	R thigh primary, T2a+	Stage III	v		v		
CY155*	Pembrolizumab	R axillary LN metastasis	R forearm primary, T4b	Stage IIId	v		v		
CY158*	Ipilimumab	Jejunal metastasis	R toe primary, T4a	Stage IV	v		v		
CY129.1*	Nivolumab<br>Pembrolizumab + CMP-<br>001	L axillary LN metastasis	Mid back primary, T4b	Stage IV	v		v		
CY161*	Nivolumab + TVEC<br>Braf + MEK inhibitors	L femoral LN metastasis<br>(including soft tissue)	L lower leg primary, T2a	Stage IIIc	v		v		
CY162*	None	R femoral LN metastasis	R lower leg primary, T4b	Stage IIIc	v		v		
CY163*	Pembrolizumab	R upper arm in-transit metastasis	R forearm primary, T4a	Stage IIIc	v		v		
CY164*	Pembrolizumab<br>TVEC	R femoral LN metastasis	R lower leg primary, T3a	Stage IIIc	v		v		
CY165*	Pembrolizumab +<br>(Epocadostat vs.<br>Placebo) x18<br>On Pembrolizumab	R axillary LN metastasis	Primary – R nasal sinus, T4b	IV	v		v		
CY184	none	R axillary LN metastasis	Primary – R back/unknown, Tx	III-b/c	v	v	v	v	
CY185	Ipilimumab x4<br>anti-KIR + Nivolumab<br>On Nivolumab	L femoral LN metastasis	Primary – vulva, T4b	III-d	v	v	v		
CY186	IFN	Small bowel metastasis	Primary – L 3rd toe, T4b	IV	v	v	v		
CY187	On Pembrolizumab	L back soft tissue metastasis (in-transit)	Primary – L posterior shoulder, T4	III-c	v	v	v		
CY188	none	L femoral LN metastasis	Primary – L lower leg, T4b	III-d	v	v	v	v	
CY189	none	R axillary LN metastasis	Primary – unknown, Tx	III-c	v	v	v	v	
CY190	Ipilimumab + Nivolumab	R femoral LN metastasis	Primary – anorectal, T4b	III-c	v	v	v	v	
CY191	none	R axillary LN metastasis	Primary – R back, T2a	III-b	v	v	v	v	
CY192	none	L external iliac LN metastasis	Primary – L lower leg, T1b	III-b/c	v	v	v	v	
CY193	none	R femoral LN metastasis	Primary – anorectal, T3b	III-c	v	v	v	v	
CY194	none	R axillary LN metastasis	Primary – unknown, Tx	III	v	v	v	v	
CY195	Ipilimumab + Nivolumab :	R femoral LN metastasis	Primary – anorectal, T4b	III-d	v	v	v	v	
CY196	none	L axillary LN metastasis	Primary – L forearm, T2a	III-b	v	v	v		
CY197P**	none	L back primary	Primary – L back, T4b	III-c	v	v	v		
CY119.2	Pembro	Small bowel (jejunum)	Primary: L back (T3a)	IV (T3a N2c M1c)					v
CY214	"Ipi + Nivo x3<br>On Nivo + TVEC"	R femoral LN	Primary: R lower leg (T3b+)	III-C (T3b+ N3c					v

* stands for subjects that were used for training set
** P stands for primary. Any P designation indicates a sample allocated from a thick primary melanoma (not metastasis).
These are rare as we can't allocate from primary samples unless they are maximum T-stage (T4b).
Theoretically, these should have the highest tumor heterogeneity.
*** CY149 and CY149A were obtained from the same surgery but different parts of a very large specimen:
CY149 was the dominant nodule/mass. CY149A was from a smaller separate nodule/mass.

Supplementary Table 1: **Description of clinical samples**
The study subjects (patient samples), their treatment, sample site, source of primary disease and stage of disease at the time of study are indicated for mass cytometry analysis. The tumors were analyzed by the phenotyping panel of antibodies and the functional panel of antibodies as indicated. In addition, a subset of these clinical samples had paired samples of peripheral blood mononuclear cells (PBMC) as indicated for analysis using the phenotyping panel of antibodies. Relevant explanatory notes are indicated at the bottom of the table.

Metal (Fluidigm)	Phenotype (Fluidigm)	Clone
Y89Di	CD45	HI30
In113Di		
In115Di		
La139Di		
Ce140Di		
Pr141Di	CCR6	G034E3
Nd142Di		
Nd143Di	CD123	6H6
Nd144Di	CD19	HIB19
Nd145Di	CD4	RPA-T4
Nd146Di	CD8a	RPA-T8
Sm147Di	CD11c	Bu15
Nd148Di	CD16	3G8
Sm149Di	CD45RO	UCHL1
Nd150Di	CD45RA	HI100
Eu151Di	CD161	HP-3G10
Sm152Di	CCR4	L291H4
Eu153Di	CD25	BC96
Sm154Di	CD27	O323
Gd155Di	CD57	HCD57
Gd156Di	CXCR3	G025H7
Gd157Di		
Gd158Di	CXCR5	J252D4
Tb159Di		
Gd160Di	CD28	CD28.2
Dy161Di	CD38	HB-7
Dy162Di		
Dy163Di	CD56	NCAM16.2
Dy164Di	TCRγδ	B1
Ho165Di		
Er166Di	CD294	BM16
Er167Di	CCR7	G043H7
Er168Di	CD14	63D3
Tm169Di		
Er170Di	CD3	UCHT1
Yb171Di	CD20	2H7
Yb172Di	CD66B	G10F5
Yb173Di	HLA-DR	LN3
Yb174Di	IgD	IA6-2
Lu175Di		
Yb176Di	CD127	A019D5
Ir191Di		
Ir193Di		
Pt195Di		
Bi209Di		

Target	Clone	Metal
CD11a	HI111	142Nd
CD4	RPA-T4	145Nd
CD8a	RPA-T8	146Nd
CD16	3G8	148Nd
CD25	2A3	149Sm
CD45	HI30	154Sm
CCR7	G043H7	159Tb
CD69	FN50	162Dy
CD45RO	UCHL1	165Ho
CD44	BJ18	166Er
CD27	O323	167Er
CD45RA	HI100	169Tm
CD3	UCHT1	170Er
CD57	HCD57	172Yb
HLA-DR	L243	174Yb
CD127	A019D5	176Yb
CD134 [OX40]	ACT35	150Nd
CD95 [Fas]	DX2	152Sm
CD366 [Tim-3]	F38-2E2	153Eu
CD279 [PD-1]	EH12.2H7	155Gd
CD152 [CTLA-4]	14D3	161Dy
CD278 [ICOS]	C398.4A	168Er
CD137 [4-1BB]	4B4-1	173Yb
CD223 [LAG3]	11C3C65	175Lu

Supplementary Table 2

Phenotype and functional panels of heavy-metal labeled antibodies for mass cytometry

Reagent	Cat. No.	Conjugatic	Clone	Company
**Antibodies**				
anti-hCEACAM5	MA5-13714	NA	Col-1	ThermoFisher Scientific
anti-hCEACAM6	MA1-17765	NA	9A6	Thermo Fisher Scientific
anti-mIgG1	555748	FITC	MOPC-21	BD Biosciences
anti-FLAG ab	F7425		polyclonal	Millipore Sigma
anti-h CD16/CD32	564219		Fc1.3216	BD Biosciences
**Confocal study**				
anti-hCD3	130-113-690	FITC	BW264/56	Miltenyi Biotec
anti-hCD4	317415	APC	OKT4	BioLegend
anti-hCEACAM1		PE	26H7	BWH (In-House)
anti-hCD19	302233	BV421	HIB19	BioLegend
anti-hCD21	354905	APC	Bu32	BioLegend
anti-hCD183 (CXCR3)	130-120-591	Biotin	REA232	Miltenyi Biotec
DyLight™ 488 Streptavidin	405218			Biolegend
**Machines**				
CytoFLEX flow cytometer				Beckman Coulter
Helios™ CyTOF2 mass cytometer				Fluidigm
BD FACSAriaTM II sorter				BD biosciences
Fluoview FV3000R resonant scanning confocal microscope				
gentleMACS™ Octo Dissociator with Heaters				Miltenyi Biotec
**Mutagenesis**				
Vector pcDNA				origene
QuikChange II Site-Directed Mutagenesis Kit				Agilent 200523

Supplementary Table 3: Study materials
Antibodies and reagents that were used in this study

B Cells: $CD45^{+}CD66b^{-}CD56^{-}CD14^{-}CD19^{+}CD3^{-}$

Naïve B Cells: $CD45^{+}CD66b^{-}CD56^{-}CD14^{-}CD19^{+}CD3^{-}CD27^{-}$

Memory B Cells: $CD45^{+}CD66b^{-}CD56^{-}CD14^{-}CD19^{+}CD3^{-}CD27^{+}$

Plasma Cells: $CD45^{+}CD66b^{-}CD56^{-}CD14^{-}CD19^{+}CD3^{-}CD27^{+}CD38^{+}CD20^{-}$

Double-Negative B Cells: $CD45^{+}CD66b^{-}CD56^{-}CD14^{-}CD19^{+}CD3^{-}CD27^{-}IgD^{-}$

Activated B Cells: $CD45^{+}CD66b^{-}CD56^{-}CD14^{-}CD19^{+}CD3^{-}CD38^{+}IgD^{+}$

Total Monocytes: $CD45^{+}CD66b^{-}CD19^{-}CD20^{-}CD3^{-}CD56^{-}HLA\text{-}DR^{+}CD123^{+}CD11c^{-}$

Classical Monocytes: $CD45^{+}CD66b^{-}CD19^{-}CD20^{-}CD3^{-}CD56^{-}HLA\text{-}DR^{+}CD123^{+}CD11c^{-}CD14^{hi}HLA\text{-}DR^{+}CD11c^{+}CD38^{+}$

Int Monocytes: $CD45^{+}CD66b^{-}CD19^{-}CD20^{-}CD3^{-}CD56^{-}HLA\text{-}DR^{+}CD14^{int}CD11c^{+}CD38^{lo\text{-}/\text{-}}$

Non-Classical Monocytes: $CD45^{+}CD66b^{-}CD19^{-}CD20^{-}CD3^{-}CD56^{-}HLA\text{-}DR^{+}CD14^{+/-}CD11c^{+}CD38^{-}$

Dendritic Cells: $CD45^{+}CD66b^{-}CD19^{-}CD20^{-}CD3^{-}CD14^{-}HLA\text{-}DR^{+}$

Immature Dendritic Cells: $CD45^{+}CD66b^{-}CD19^{-}CD20^{-}CD3^{-}CD14^{-}HLA\text{-}DR^{+}CD123^{-}CD11c^{+}CD38^{-}$

Mature Dendritic Cells: $CD45^{+}CD66b^{-}CD19^{-}CD20^{-}CD3^{-}CD14^{-}HLA\text{-}DR^{+}CD123^{-}CD11c^{+}CD38^{+}$

Plasmacytoid Dendritic Cells: $CD45^{+}CD66b^{-}CD19^{-}CD20^{-}CD3^{-}CD14^{-}HLA\text{-}DR^{+}CD123^{+}CD11c^{-}$

$CD4^{+}$ T Cells: $CD45^{+}CD66b^{-}CD19^{-}CD20^{-}CD14^{-}CD11c^{-}CD3^{+}TCR\gamma\delta^{-}CD4^{+}CD8^{-}$

Naïve $CD4^{+}$ T Cells: $CD45^{+}CD66b^{-}CD19^{-}CD20^{-}CD14^{-}CD11c^{-}CD3^{+}TCR\gamma\delta^{-}$ $CD4^{+}CD8^{-}CCR7^{hi}$ $CD45RA^{+}CD45RO^{-}$

Central-Memory $CD4^{+}$ T Cells: $CD45^{+}CD66b^{-}CD19^{-}CD20^{-}CD14^{-}CD11c^{-}CD3^{+}TCR\gamma\delta^{-}$ $CD4^{+}CD8^{-}CCR7^{hi}$ $CD45RA^{-}CD45RO^{+}$

Effector-Memory $CD4^{+}$ T Cells: $CD45^{+}CD66b^{-}CD19^{-}CD20^{-}CD14^{-}CD11c^{-}CD3^{+}TCR\gamma\delta^{-}$ $CD4^{+}CD8^{-}CCR7^{hi}$ $CD45RA^{-}CD45RO^{+}CD27^{+}$

Terminal-Effector $CD4^{+}$ T Cells: $CD45^{+}CD66b^{-}CD19^{-}CD20^{-}CD14^{-}CD11c^{-}CD3^{+}TCR\gamma\delta^{-}$ $CD4^{+}CD8^{-}CCR7^{hi}$ $CD45RA^{-}CD45RO^{+}CD27^{-}$

Regulatory $CD4^{+}$ T Cells:$CD45^{+}CD66b^{-}CD19^{-}CD20^{-}CD14^{-}CD11c^{-}CD3^{+}TCR\gamma\delta^{-}$ $CD4^{+}CD8^{-}CCR4^{+}CD45RA^{-}CD45RO^{+}CD25^{high}CD127^{lo/-}$

Follicular Helper $CD4^{+}$ T Cells:$CD45^{+}CD66b^{-}CD19^{-}CD20^{-}CD14^{-}CD11c^{-}CD3^{+}TCR\gamma\delta^{-}$ $CD4^{+}CD8^{-}CD45RA^{-}CD45RO^{+}PD1^{high}CXCR5^{+}$

Th1-like $CD4^{+}$ T Cells: $CD45^{+}CD66b^{-}CD19^{-}CD20^{-}CD14^{-}CD11c^{-}CD3^{+}TCR\gamma\delta^{-}$ $CD4^{+}CD8^{-}CD45RO^{+}CXCR5^{-}CCR4^{-}CXCR3^{+}CCR6^{-}$

Th2-like $CD4^{+}$ T Cells: $CD45^{+}CD66b^{-}CD19^{-}CD20^{-}CD14^{-}CD11c^{-}CD3^{+}TCR\gamma\delta^{-}$ $CD4^{+}CD8^{-}CD45RO^{+}CXCR5^{-}CCR4^{+}CXCR3^{-}CCR6^{-}$

Th17-like $CD4^{+}$ T Cells: $CD45^{+}CD66b^{-}CD19^{-}CD20^{-}CD14^{-}CD11c^{-}CD3^{+}TCR\gamma\delta^{-}$ $CD4^{+}CD8^{-}CD45RO^{+}CXCR5^{-}CCR4^{-}CXCR3^{+}CCR6^{+}$

$CD8^{+}$ T Cells: $CD45^{+}CD66b^{-}CD19^{-}CD20^{-}CD14^{-}CD11c^{-}CD3^{+}TCR\gamma\delta^{-}CD4^{-}CD8^{+}CD161^{lo/-}$

Naïve $CD8^{+}$ T Cells $CD45^{+}CD66b^{-}CD19^{-}CD20^{-}CD14^{-}CD11c^{-}CD3^{+}TCR\gamma\delta^{-}CD4^{-}CD8^{+}CD161^{lo/-}CCR7^{hi}$ $CD45RA^{+}CD45RO^{-}$

Central-Memory $CD8^{+}$ T Cells : $CD45^{+}CD66b^{-}CD19^{-}CD20^{-}CD14^{-}CD11c^{-}CD3^{+}TCR\gamma\delta^{-}CD4^{-}CD8^{+}CD161^{lo/-}CCR7^{hi}$ $CD45RA^{-}CD45RO^{+}$

Effector-Memory $CD8^{+}$ T Cells :$CD45^{+}CD66b^{-}CD19^{-}CD20^{-}CD14^{-}CD11c^{-}CD3^{+}TCR\gamma\delta^{-}CD4^{-}CD8^{+}CD161^{lo/-}CCR7^{lo/-}CD45RA^{-}CD45RO^{+}CD27^{+}$

Terminal-Effector $CD8^{+}$ T Cells :$CD45^{+}CD66b^{-}CD19^{-}CD20^{-}CD14^{-}CD11c^{-}CD3^{+}TCR\gamma\delta^{-}CD4^{-}CD8^{+}CD161^{lo/-}CCR7^{lo/-}CD45RA^{-}CD45RO^{+}CD27^{-}$

$CD8^{+}$ TSCM Cells: $CD45^{+}CD66b^{-}CD19^{-}CD20^{-}CD14^{-}CD11c^{-}CD3^{+}TCR\gamma\delta^{-}CD4^{-}CD8^{+}CD161^{lo/-}$-$CD57^{-}CD127^{high}CCR7^{+}CD27^{+}CXCR3^{+}CXCR5^{+}PD1^{high}$

$CD8^{+}$ TEMRA Cells: $CD45^{+}CD66b^{-}CD19^{-}CD20^{-}CD14^{-}CD11c^{-}CD3^{+}TCR\gamma\delta^{-}CD4^{-}CD8^{+}CD161^{lo/-}$-$CD57^{+}CD127^{low}CCR7^{-}CD27^{-}CXCR3^{+}CXCR5^{+}PD1^{high}$

NK: $CD45^{+}CD66b^{-}CD19^{-}CD20^{-}CD14^{-}CD45RA^{+}CD123^{-}CD56^{+}$

MAIT/ NKT: $CD45^{+}CD66b^{-}CD19^{-}CD20^{-}CD14^{-}CD11c^{-}CD3^{+}CD4^{-}CD28^{+}CD161^{hi}$

Supplementary Table 4: Markers used for cellular annotation

The markers used for annotation of cellular subsets are indicated. Each cell subset based upon a series of shared markers (blue) with the addition of other markers to parse out specific subtypes (red).
