# Supplementary material for: High-dimensional mapping of human CEACAM1 expression on immune cells and association with melanoma drug resistance
Source: Commun Med (Lond). 2024 Jul 2;4:128. doi: 10.1038/s43856-024-00525-8 (PMC11219841; doi:10.1038/s43856-024-00525-8)
Supplement: Supplementary file 2 — Description of Additional Supplementary Files [file 43856_2024_525_MOESM2_ESM.pdf]

Description of Additional Supplementary Files

File Name: Supplementary Data 1

Description: Numerical source data used to create Fig. 1f, 1h in the manuscript.

File Name: Supplementary Data 2

Description: Numerical source data used to create Fig. 2c, 2g in the manuscript.

File Name: Supplementary Data 3

Description: Numerical source data used to create Fig. 3c, 3e, 3f, 3i, 3m and parameters used to create FLOWSOM outputs as in Fig. 3g in the manuscript.

File Name: Supplementary Data 4

Description: Numerical source data used to create Fig. 4c, 4d, 4e, 4g, 4h, 4i, 4l, 4n, 4s in the manuscript.

File Name: Supplementary Data 5

Description: Numerical source data used to create Fig. 5c, 5d, 5e, 5g, 5h, 5i, 5l, 5n, 5s and parameters used to create FLOWSOM outputs as in Fig. 5j in the manuscript.

File Name: Supplementary Data 6

Description: Numerical source data used to create Fig. 6c, 6d, 6e, 6g, 6i, 6j, 6k, 6o, 6p in the manuscript.

File Name: Supplementary Data 7

Description: Numerical source data used to create Fig. 7e, and 7h; Parameters used to create FLOWSOM outputs as in Fig. 7b, 7c, 7d, 7e, 7f, 7g in the manuscript.

File Name: Supplementary Data 8

Description: Numerical source data used to create Fig. 8c, 8d, 8e, 8h, 8j, 8n, 8p; Parameters used to create FLOWSOM outputs as in Fig. 8f-k in the manuscript.
