# Supplementary material for: High-dimensional mapping of human CEACAM1 expression on immune cells and association with melanoma drug resistance
Source: Commun Med (Lond). 2024 Jul 2;4:128. doi: 10.1038/s43856-024-00525-8 (PMC11219841; doi:10.1038/s43856-024-00525-8)
Supplement: Supplementary file 11 — reporting summary [file 43856_2024_525_MOESM11_ESM.pdf]

nature portfolio

Corresponding author(s): Yu-Hwa Huang & Richard S. Blumberg

Last updated by author(s): 2023/10/03

Reporting Summary

Nature Portfolio wishes to improve the reproducibility of the work that we publish. This form provides structure for consistency and transparency in reporting. For further information on Nature Portfolio policies, see our Editorial Policies and the Editorial Policy Checklist.

Statistics

For all statistical analyses, confirm that the following items are present in the figure legend, table legend, main text, or Methods section.

n/a	Confirmed	
☐	☒	The exact sample size (*n*) for each experimental group/condition, given as a discrete number and unit of measurement
☐	☒	A statement on whether measurements were taken from distinct samples or whether the same sample was measured repeatedly
☐	☒	The statistical test(s) used AND whether they are one- or two-sided<br>*Only common tests should be described solely by name; describe more complex techniques in the Methods section.*
☐	☒	A description of all covariates tested
☐	☒	A description of any assumptions or corrections, such as tests of normality and adjustment for multiple comparisons
☐	☒	A full description of the statistical parameters including central tendency (e.g. means) or other basic estimates (e.g. regression coefficient) AND variation (e.g. standard deviation) or associated estimates of uncertainty (e.g. confidence intervals)
☐	☒	For null hypothesis testing, the test statistic (e.g. *F*, *t*, *r*) with confidence intervals, effect sizes, degrees of freedom and *P* value noted<br>*Give P values as exact values whenever suitable.*
☒	☐	For Bayesian analysis, information on the choice of priors and Markov chain Monte Carlo settings
☐	☒	For hierarchical and complex designs, identification of the appropriate level for tests and full reporting of outcomes
☒	☐	Estimates of effect sizes (e.g. Cohen's *d*, Pearson's *r*), indicating how they were calculated

Our web collection on statistics for biologists contains articles on many of the points above.

Software and code

Policy information about availability of computer code

Data collection	All software used for data collection are publicly or commercially available: For isolation of the tumor-dissociated cells, tumor biopsies were subjected to mechanical and enzymatic dissociation system using gentleMACS™ Octo Dissociator with Heaters (Miltenyi Biotec); for cell number determination, Countess automated cell counter (Invitrogen) was used for cell counting; for immunofluorescence images, Fluoview FV3000R resonant scanning confocal microscope equipped with 4 laser lines (405, 488, 514, and 633 nm) was used for immunofluorescence images; for mutagenesis, thermal cycler, Veriti 96 well thermal cycler (Applied Biosystems) was applied and qPCR was run on a CFX96 Real-Time System (Bio-Rad) and analyzed with Bio-Rad CFX Maestro; for flow cytometry, data was acquired with a Cytoflex flow cytometer (Beckman Coulter); for immunoblotting, Gel Doc XR+ System (Bio-Rad) Image Lab Image Capture and Analysis Software (Bio-Rad) and unsaturated films were digitally scanned and band intensities (densitometric analysis) quantified using ImageJ (NIH); for mass cytometry, data was acquired with a CyTOF-Helios (Fluidigm).
Data analysis	All software used for data analysis are publicly or commercially available: For mass cytometry, cells were deconvolved using FlowJo software (TreeStar, V10 for MacOS), then the data were randomized with the Fluidigm acquisition software and normalized with the FlowJo normalizer. Transformed data files were concatenated into randomly sampled cells using R (v.3.3.2) for each study group. For dimensionality reduction analyses of multiplexed single-cell mass cytometry data, the viSNE tool was firstly used to apply the Barnes-Hut implementation of the t-Distribution Stochastic Neighbor Embedding (tSNE) algorithm. After the data global structures were visualized using viSNE and their structure dissected into distinct immune populations by re-embedding the manual-gates that define them, markers of interest were further exported from the specific immune populations which then were interrogated with appropriate statistical approaches to validate the data variance and significance between groups. Further, the data were then integrated by manual gating or with high-dimensional analysis algorithms. Algorithms and software kits were applied using Cytobank as a platform to facilitate analysis of CyTOF datasets. For biomarker discovery of which immune population or markers were significantly different between the five groups of this study, we performed analysis to stratify signatures from clustered data features that explain differences between the 5 clinical sample groups by Citrus (cluster identification, characterization, and regression) analysis by employing Significance Analysis of Microarrays (SAM) as a correlative model to identify features that correlate with an endpoint at different false discovery rates, Nearest Shrunken Centroid (PAMR) and L1-Penalized Regression (LASSO/

GLMNET) as predictive models. We also used the SPADE clustering algorithm to pinpoint the correlations between marker expression.

For manuscripts utilizing custom algorithms or software that are central to the research but not yet described in published literature, software must be made available to editors and reviewers. We strongly encourage code deposition in a community repository (e.g. GitHub). See the Nature Portfolio guidelines for submitting code & software for further information.

Data

Policy information about availability of data

All manuscripts must include a data availability statement. This statement should provide the following information, where applicable:

- Accession codes, unique identifiers, or web links for publicly available datasets
- A description of any restrictions on data availability
- For clinical datasets or third party data, please ensure that the statement adheres to our policy

Data available upon request

Field-specific reporting

Please select the one below that is the best fit for your research. If you are not sure, read the appropriate sections before making your selection.

☒ Life sciences ☐ Behavioural & social sciences ☐ Ecological, evolutionary & environmental sciences

For a reference copy of the document with all sections, see nature.com/documents/nr-reporting-summary-flat.pdf

Life sciences study design

All studies must disclose on these points even when the disclosure is negative.

Sample size	The study consisted of healthy donor peripheral blood mononuclear cells or PBMC (n=5), treatment-naïve PBMC (n=7), treatment-naïve PBMC (n=3), treatment-naïve tumors (n=9), treatment-resistant tumors (n=10).
Data exclusions	Not applicable
Replication	We used a training cohort of treatment-naive (n=7) and -resistant (n=13) tumor dissociated cells to establish the parameters for the mass cytometry settings.
Randomization	Samples were collected from study subjects based on being naïve or resistant to immunotherapy.
Blinding	Once samples were binned according to treatment status, they were analyzed as a group by data driven algorithms based upon clinical phenotype (healthy, naïve, resistant) and sample source (peripheral blood mononuclear cells or tumors).

Reporting for specific materials, systems and methods

We require information from authors about some types of materials, experimental systems and methods used in many studies. Here, indicate whether each material, system or method listed is relevant to your study. If you are not sure if a list item applies to your research, read the appropriate section before selecting a response.

Materials & experimental systems

n/a	Involved in the study	
☐	☒	Antibodies
☐	☒	Eukaryotic cell lines
☒	☐	Palaeontology and archaeology
☒	☐	Animals and other organisms
☐	☒	Human research participants
☒	☐	Clinical data
☒	☐	Dual use research of concern

Methods

n/a	Involved in the study	
☒	☐	ChIP-seq
☐	☒	Flow cytometry
☒	☐	MRI-based neuroimaging

Antibodies

Antibodies used	Antibodies used against CD45 (HI30), CCR6 (G034E3), CD123 (6H6), CD19 (HIB19), CD4 (RPA-T4), CD8a (RPA-T8), CD11c (Bu15), CD16 (3G8), CD45RO (UCHL1), CD45RA (HI100), CD161 (HP-3G10), CCR4 (L291H4), CD25 (BC96), CD27 (O323), CD57 (HCD57), CXCR3 (G025H7), CXCR5 (J252D4), CEACAM1 (26H7), CD28 (CD28.2), CD38 (HB-7), CD56 (NCAM16.2), TCRγδ (B1), CD294 (BM16), CCR7 (G043H7), CD14 (63D3), PD1 (EH12.2H7), CD3 (UCHT1), CD20 (2H7), CD66B (G10F5), HLA-DR (LN3), IgD (IA6-2), PD-L1 (B7H1), CD127 (A019D5), CD11a (HI111), CD25 (2A3), CD69 (FN50), CD44 (BJ18), HLA-DR (L243), CD134 [OX40] (ACT35), CD95 [Fas] (DX2), CD366 [Tim-3] (F38-2E2), CD152 [CTLA-4] (14D3), CD278 [ICOS] (C398.4A), CD137 [4-1BB] (4B4-1), and CD223 [LAG3] (11C3C65) were

	obtained from Fluidigm. Antibodies used against Ceacam5 (MA5-13714, Col-1) and Ceacam6 (MA1-17765, 9A6) were obtained from ThermoFisher Scientific. Antibodies used against mIgG1 (555748, MOPC-21) and CD16/CD32 (564219, Fc1.3216) were obtained from BD biosciences. Antibody used against FLAG (F7425, polyclonal) was obtained from Millipore Sigma. Antibodies used against CD4 (317415, OKT4), CD19 (302233, HIB19) and CD21 (354905, Bu32) were obtained from Biolegend. Antibodies used against CD3 (130-113-690, BW264/56) and CD183 (CXCR3) (130-120-591, REA232) were obtained from Miltenyi Biotech.
Validation	Antibodies used in this study are commercially available and have been validated by the manufacturer, previous studies by other groups, or studies previously published by our lab and cited in the text. For 26H7 antibody, we have previously published on its specificity and further assessed it internally with our human CEACAM family transfectants as described in the current report.

Eukaryotic cell lines

Policy information about cell lines

Cell line source(s)	HeLa<br>HEK293T
Authentication	Obtained and authenticated by ATCC
Mycoplasma contamination	PCR analysis confirming the absence of mycoplasma contamination is performed routinely in the laboratory
Commonly misidentified lines<br>(See ICLAC register)	There are no commonly misidentified lines used in the study

Human research participants

Policy information about studies involving human research participants

Population characteristics	The surgeon (C.H.Y.) identified operative patients with metastatic melanoma who were appropriate for sampling of fresh tissue during curative or palliative metastasectomies. The surgeon allocated tissue samples at the end of the operation from regions most likely to harbor viable tumor without interfering with diagnosis or clinical staging.
Recruitment	There was no selection bias as the patients were recruited based upon the likelihood that viable tumor could be obtained. The patients were also categorized based upon whether they were treatment naive or treatment failures and an attempt was made to include equal numbers of these patent categories in the experimental study.
Ethics oversight	Approval received from the Institutional Review Board (IRB) of the Brigham and Women's Hospital (BWH) and the Dana Farber Cancer Institute.

Note that full information on the approval of the study protocol must also be provided in the manuscript.

Flow Cytometry

Plots

Confirm that:

- [x] The axis labels state the marker and fluorochrome used (e.g. CD4-FITC).
- [x] The axis scales are clearly visible. Include numbers along axes only for bottom left plot of group (a 'group' is an analysis of identical markers).
- [x] All plots are contour plots with outliers or pseudocolor plots.
- [x] A numerical value for number of cells or percentage (with statistics) is provided.

Methodology

Sample preparation	Sample preparations of individual cell types are described for each in detail in the Methods section.
Instrument	Flow cytometry was performed using the CytoFlex Flow Cytometer.
Software	Data were analyzed using FlowJo software (TreeStar, V10 for MacOS)
Cell population abundance	Cell population abundance was determined using Flow cytometer (Beckman Coulter) and Helios CyTOF (Fludigm)
Gating strategy	Detailed markers used for defining immune cell populations by manual gating were listed in Extended Data Table 3.

- [x] Tick this box to confirm that a figure exemplifying the gating strategy is provided in the Supplementary Information.
